# Retrospective Analysis of NIST Standard Reference Material 1450, Fibrous Glass Board, for Thermal Insulation Measurements

**DOI:** 10.6028/jres.119.012

**Published:** 2014-08-25

**Authors:** Robert R Zarr, N Alan Heckert, Stefan D Leigh

**Affiliations:** National Institute of Standards and Technology, Gaithersburg, MD 20899

**Keywords:** bulk density, certified reference material, fit, guarded hot plate, high density molded fibrous glass board, model, regression analysis, standard reference material, thermal conductivity, thermal insulation

## Abstract

Thermal conductivity data acquired previously for the establishment of Standard Reference Material (SRM) 1450, Fibrous Glass Board, as well as subsequent renewals 1450a, 1450b, 1450c, and 1450d, are re-analyzed collectively and as individual data sets. Additional data sets for proto-1450 material lots are also included in the analysis. The data cover 36 years of activity by the National Institute of Standards and Technology (NIST) in developing and providing thermal insulation SRMs, specifically high-density molded fibrous-glass board, to the public. Collectively, the data sets cover two nominal thicknesses of 13 mm and 25 mm, bulk densities from 60 kg·m^−3^ to 180 kg·m^−3^, and mean temperatures from 100 K to 340 K. The analysis repetitively fits six models to the individual data sets. The most general form of the nested set of multilinear models used is given in the following equation:

$$\lambda (\rho ,T)=a_{0}+a_{1}\rho +a_{2}T+a_{3}T^{3}+a_{4}e^{-{\left(\frac{T-a_{5}}{a_{6}}\right)}^{2}}$$

where λ(ρ,*T*) is the predicted thermal conductivity (W·m^−1^·K^−1^), ρ is the bulk density (kg·m^−3^), *T* is the mean temperature (K) and *a_i_* (for *i* = 1, 2, … 6) are the regression coefficients. The least squares fit results for each model across all data sets are analyzed using both graphical and analytic techniques. The prevailing generic model for the majority of data sets is the bilinear model in ρ and *T*.

$$\lambda (\rho ,T)=a_{0}+a_{1}\rho +a_{2}T$$

One data set supports the inclusion of a cubic temperature term and two data sets with low-temperature data support the inclusion of an exponential term in *T* to improve the model predictions. Physical interpretations of the model function terms are described. Recommendations for future renewals of SRM 1450 are provided. An Addendum provides historical background on the origin of this SRM and the influence of the SRM on external measurement programs.

## 1. Introduction

During the past 36 years, the National Institute of Standards and Technology (NIST^1^) has issued five production lots of Standard Reference Material^®2^ (SRM) 1450, Fibrous Glass Board, for thermal insulation measurements. The lots, designated 1450, 1450a, 1450b, 1450c, and most recently 1450d, have been issued with certified value assignments for thermal resistance, thermal conductivity, and, for 1450d, bulk density as well. These thermal insulation SRMs have been, and currently are, utilized by industry, academia, and government in standard test methods for the purposes of checking guarded-hot-plate apparatus [1], calibrating heat-flow-meter apparatus [2], and, when necessary, for checking or calibrating hot-box apparatus [3].

Over time, it has come to the attention of the authors that, although the above lots are considered to be essentially the same material in terms of composition and macroscopic properties, the resulting certified formulaic thermal characterizations of the lots are different. It is natural to question why there are differences, and to what extent these differences are significant and real. This paper re-examines, from the advantage of a retrospective viewpoint, SRMs 1450 through 1450d (as well as proto-production material lots) and re-evaluates models used for the thermal characterizations of the individual lots.

It is important to emphasize that the results of this retrospective analysis, specifically the regression equations for the individual data sets considered herein, are based on the original (not new) data for SRMs 1450 through 1450d. The regression equations in this paper are not intended to be, and cannot be, used to “re-certify” any of these previous SRMs. Customers are advised to retain the original certificate equations for their intended purposes. The results of this analysis, instead, aim to enhance our understanding of the original certificate equations derived by previous NBS researchers as well as to improve the development and modeling of future thermal insulation SRMs.

This paper documents the historical development of the thermal insulation SRM program at NIST and discusses the evolution of the technical production and certification of SRMs 1450–1450d. The collective SRM data are reviewed graphically and the original certificate regression equations with (expanded) uncertainties are described. Individual data sets, including 1450 through 1450d and proto-1450 materials, are re-analyzed by analytical and graphical approaches. Physical mechanisms for the regression terms are suggested. Recommendations for the development of future thermal insulation SRMs are given. Supplementary information on the data and analyses, in the form of zipped files, is provided online^3^.

## 2. Historical Development

As part of the centennial commemoration of the institution [4], Zarr chronicled an account of the thermal insulation and building materials testing program at NBS/NIST from 1912 to 2001 [5]. The publication describes the early and continued development of the guarded-hot-plate apparatus at NBS/NIST and the subsequent standardization of the test method in 1945 [6]. The initiation of thermal insulation reference materials in the 1970s, as part of the NIST SRM Program, was a major advance in the effort to improve the accuracy of the test method. It should be noted, however, that the thermal insulation SRM program owes its success, in part, to a preceding calibration program.

Prior to 1958, customers would submit their own test specimens to NBS for accurate determinations of thermal resistance. In 1958, the Heat Transfer Section, under H. E. Robinson, of the NBS Building Research Division responded to increasing requests for “thermal conductivity reference specimens” by stockpiling two materials having satisfactory characteristics of homogeneity and stability – fibrous-glass board and gum rubber. From 1958 to 1978, NBS provided over 300 pairs of “calibrated thermal conductivity reference specimens” [7], commonly known to industry and government as “NBS Fibrous Glass Board.” The specimens were selected from one of four lots of fibrous-glass board which were identified internally at NBS by the year of their acquisition (1958, 1959, 1961, and 1970).

In the early 1970s, the ASTM Sub-Committee C16.30 on Thermal Properties (now Thermal Measurements) established a working task group to undertake a comprehensive review of candidate reference materials for low thermal conductivity [8]. The findings of the working group were formally published in a 1978 position paper advocating an SRM approach for thermal insulation reference materials [9]. The main reason was to make available “a common set of uniform and reproducible materials (SRMs)” in order to launch “a cooperative measurements program … to improve all measurements as well as to correct unreliable apparatus, inadequate techniques, and to standardize procedures” [9]. The proposed SRM program was intended to complement a “realistic” thermal insulation accreditation program^4^ that was under development during the same period.

The position paper [9] recommended a comprehensive plan entailing five phases for establishing a thermal insulation SRM program with the National Bureau of Standards having a central role in the overall effort. In response, NBS through the Office of Standard Reference Materials immediately agreed to collaborate on the first two phases. In phase one, NBS calibration data that had been acquired over twenty years from 1958 to 1978 (as part of the former calibration program) were to be systematically analyzed and used to certify the remaining stock of fibrous-glass board over a limited temperature range of 260 K to 325 K. For phase two, new stock was to be procured and characterized over an extended temperature range. The production lots for SRMs 1450–1450b that were established for phase one and phase two, as well as subsequent renewals, are described in Sec. 3.

Subsequent phases of the ASTM C16.30 plan proposed both short- and long-term studies of several low thermal conductivity candidate materials for development as potential reference materials. Based on the recommended plan, NBS/NIST developed the following thermal insulation SRMs:

- Fibrous-glass blanket: SRMs 1451 (now obsolete) and 1452; and,
- Fumed-silica board: SRMs 1449 and 1459 (dimensionally smaller unit).

In 1996, after receiving a separate request from the National Fenestration Rating Council (NFRC), NIST issued SRM 1453, Expanded Polystyrene Board, for use in the calibration procedure for testing windows in a hot box. A description of the other thermal insulation standard reference materials (1451, 1452, 1449, 1459, and 1453) has been presented elsewhere [15].

## 3. SRM 1450 Production Lots

**Fig. 1:**
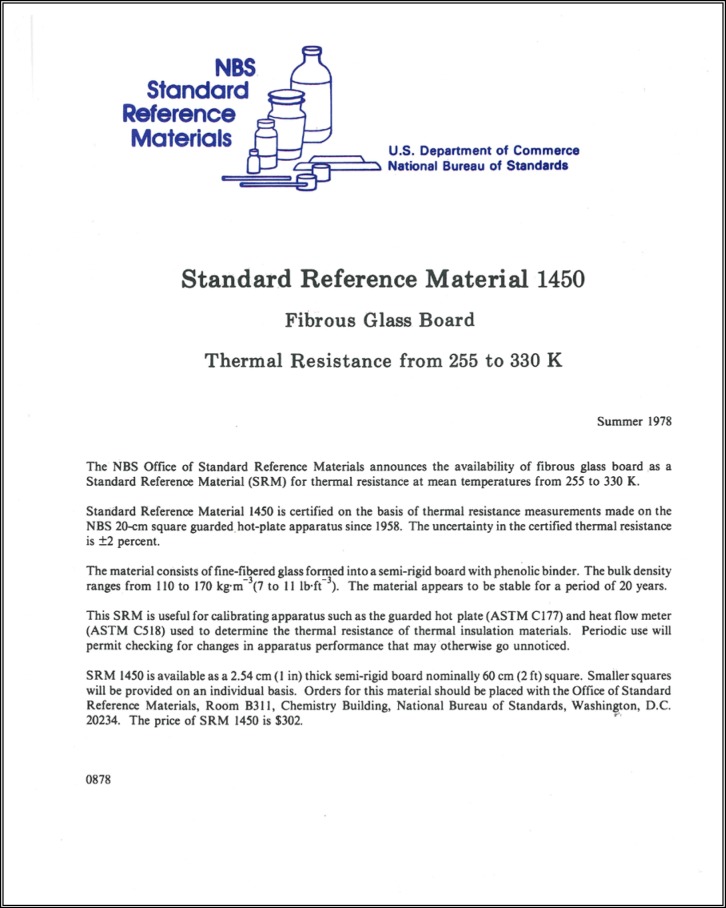
Copy of 1978 announcement for NBS SRM 1450, Fibrous Glass Board.

Standard Reference Material 1450 was issued to the public in 1978. A copy of the original announcement is available in Fig. 1. Table 1 summarizes the chronology of SRM 1450 and includes information for year acquired, year issued, references on the technical development of each SRM, where available, and laboratory facility. When a batch-certified SRM lot is exhausted, the renewal (i.e., replacement lot) retains the original number designation and a lower case letter (a, b, c, etc.) is appended to denote the new lot. Revisions to the certificates due to modifications, corrections, or other changes are noted on the Certificate Revision History and, in this paper, are denoted by a Roman numeral (I, II, etc.).

There have been four guarded-hot-plate laboratory facilities utilized at NBS/NIST for the thermal characterization of 1450 and renewals, indicated in Table 1 with superscripts (b, c, d, and e). One unique designation, 1450b, was jointly characterized by aggregation of data from the Center for Chemical Engineering (CCE) in Boulder, Colorado and the Center for Building Technology (CBT) in Gaithersburg, Maryland. In 1982, 1450b(I) was issued with certified values over a moderate temperature range and informational values below 255 K. After conducting additional low-temperature measurements at Boulder, Colorado, NBS re-issued 1450b(II) with certified values from 100 K to 330 K. Standard Reference Material 1450c(I) was initially issued in 1997 and was re-issued in 2010 with revised certification values for thermal resistance (1450c(II)).

### 3.1 Material

The 1450 production lots have been stocked with commercial materials obtained from various U.S. thermal insulation manufacturers. Generally speaking, the material is a semi-rigid or rigid board consisting of discontinuous glass fibers that are bonded by a thermosetting resin, typically a phenolic binder formulation. The high-density boards are formed by molding, under heat and pressure, individual layers of glass-fiber pelts treated with uncured binder. The thickness and bulk density of a board are controlled by the construction of the pelts and by the number of pelts in a board. After curing of the binder at an elevated temperature and subsequent removal from the mold, the board is cooled and cut to final lateral dimensions. In the fabrication process, the glass fibers are arranged arbitrarily in layers parallel to the board faces and perpendicular to the direction of heat flow used in thermal resistance measurements across the thickness of the board. For testing purposes, the organic binder limits the upper temperature of the material to 423 K [9], although the 1450 Certificates limit the conditioning temperature to a precautionary 380 K. The nominal dimensions of an SRM unit are 25 mm in thickness by 610 mm by 610 mm.

Over the past 56 years, the suppliers of the commercial products obtained for the SRM program have changed, as well as the manufacturing process itself. In general, the material has changed due to improvements in technology including different machines, settings, and formulations, among other factors. Although the fabrication process has not been documented by NIST, primarily because the technical details are proprietary, an abbreviated historical account of the production of glass wool and glass fiber (from 1958 to 2010) can be found in the literature [19–23]. Additional information on the effect of the material factor is discussed in Sec. 4.2.2.

### 3.2 Certification Procedure

**Fig. 2:**
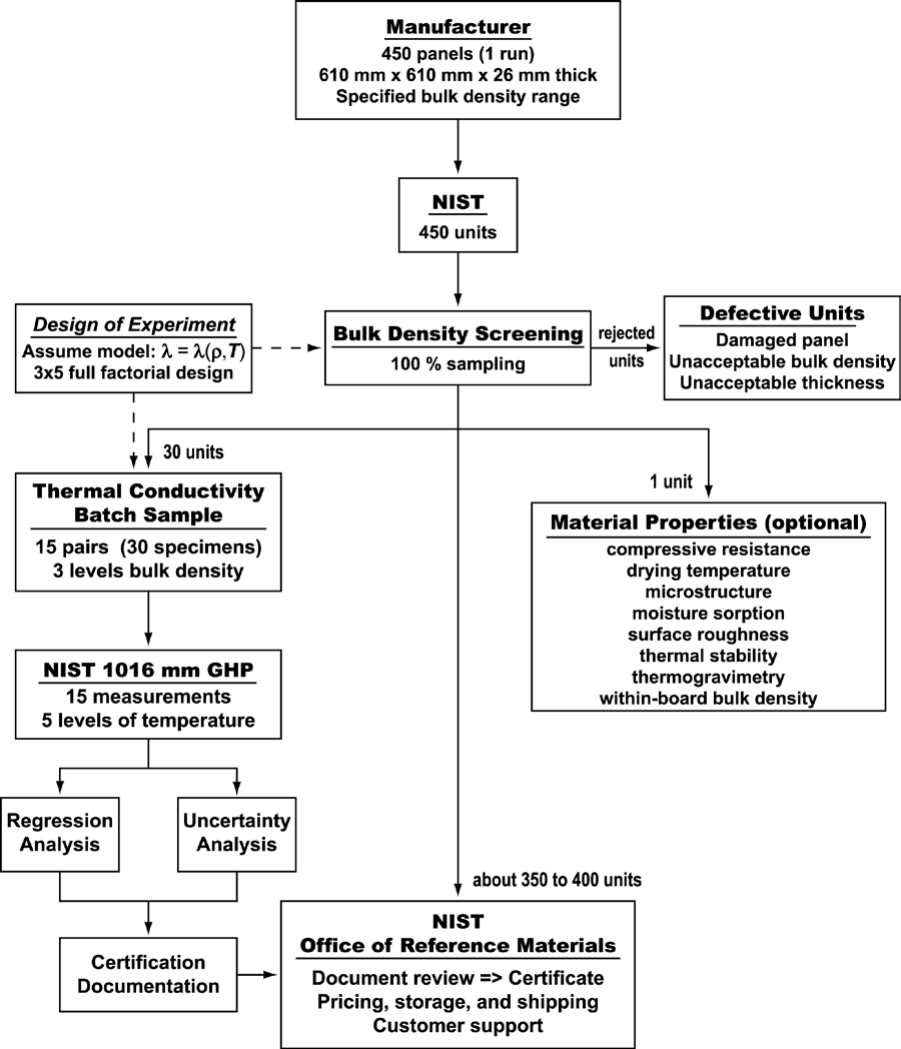
Certification project plan for SRM 1450d renewal.

The three major sequential stages for establishing a NIST SRM [24] are 1) planning and research; 2) production and certification, and 3) distribution. The first stage, planning and research, involves gathering information based on industry needs (Sec. 2), assessing priorities, and includes several additional steps that can require years to examine and evaluate candidate materials. The second and third stages are shown schematically in Fig. 2. Figure 2 outlines the process for the fabrication, (batch) certification, and distribution of NIST SRM 1450d, which includes the following steps:

1. procurement of material per NIST requirements (based on industry needs);
2. development of a statistically justified sampling and measurement plan;
3. bulk density measurements (including homogeneity testing) of material lot (currently 100 % sampling);
4. stratified sampling (15 pairs of specimens containing low, mid, and high bulk density strata);
5. thermal conductivity measurements of a statistical sample using the NIST 1016 mm guarded-hotplate apparatus; and,
6. analysis of data leading to (batch) certification.

Since it is impractical to measure the thermal conductivity of every specimen, a statistically justifiable sampling scheme is used to select specific specimens from the material lot for testing in the guarded-hot-plate apparatus. The analysis of the thermal conductivity data of the sample is subsequently used for certification of the entire SRM lot. The batch approach allows the simultaneous characterization and certification of a large quantity of comparable units that are economically produced and available on demand. In contrast to a calibration measurement, a thermal insulation SRM unit issued to a customer, prepared under batch certification, has not been measured directly in a NIST guarded-hot-plate apparatus. Consequently, the uncertainty statement for a thermal insulation SRM usually contains a component of uncertainty (typically small) attributable to the material lot variability.

The third stage of SRM production, administrative functions, is handled by the NIST Office of Reference Materials (ORM) and includes customer support, document review, approval and printing of certificates, pricing, packaging, storage, and distribution of artifacts (Fig. 2). In practice, thermal insulation SRM lots are prepared with a sufficient number of units to meet anticipated demand for 10 years. With the exception of the original 1450 lot and 1450a renewal, each lot was stocked with approximately 350 to 400 units. The sample group of 15 specimen pairs used for thermal conductivity measurements (Fig. 2) is usually retained and archived for future reference.

### 3.3 Supplemental Material Properties

Figure 2 also illustrates (an optional set of) supplemental of material properties that, over time, have been investigated by NBS/NIST researchers for selected material lots. The primary purpose of the investigations was not to certify additional properties but, rather, to determine what, if any, are the effects of other (secondary) factors on the certified properties of thermal resistance and thermal conductivity. The data obtained for the supplemental properties are considered informational in nature and are noted as such when included in the certificate. Table 2 summarizes the supplementary properties determined by NBS/NIST researchers for various SRM designations.

### 3.4 Graphical Overview of SRM 1450 Data

**Fig 3:**
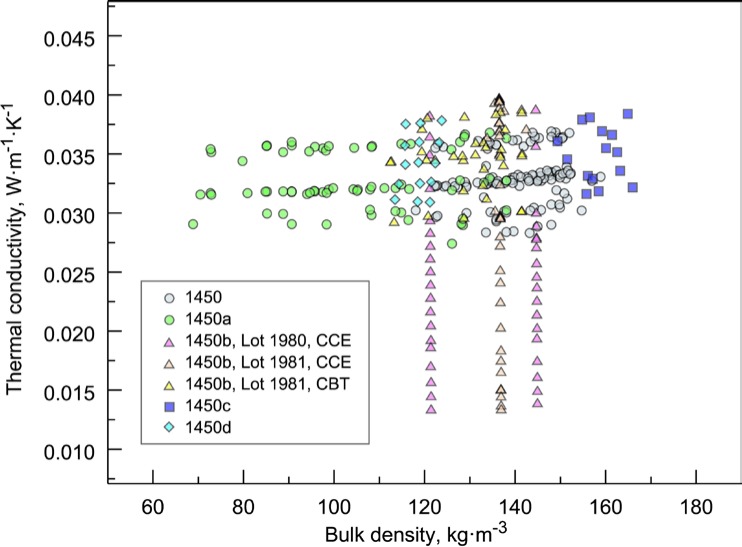
Thermal conductivity as a function of bulk density for NIST SRMs 1450, 1450a, 1450b, 1450c, and 1450d Fibrous Glass Board (CCE, Center for Chemical Engineering in Boulder, Colorado; CBT, Center for Building Technology in Gaithersburg, Maryland).

**Fig. 4:**
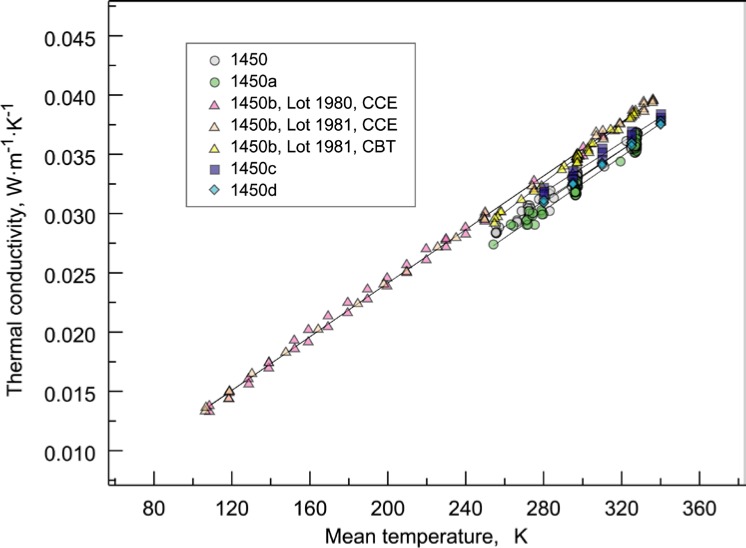
Thermal conductivity as a function of mean temperature for NIST SRMs 1450, 1450a, 1450b, 1450c, and 1450d Fibrous Glass Board (CCE, Center for Chemical Engineering in Boulder, Colorado; CBT, Center for Building Technology in Gaithersburg, Maryland).

The thermal conductivity data for SRMs 1450, 1450a, 1450b(II), 1450c(II), and 1450d are plotted as a function of bulk density and mean temperature in Figs. 3 and 4, respectively. These data have been reassembled from internal sources in possession of the first author or from previous publications [16–18]. It is plainly visible from the data displayed in Figs. 3 and 4 that thermal conductivity is a strong linear function of mean temperature (Fig. 4) and a weak linear function of bulk density (Fig. 3). The distinct levels in thermal conductivity observable in Fig. 3 for a particular SRM data set are principally due to the temperature dependency displayed in Fig. 4. For a given SRM data set, an upward shift corresponds to data at higher mean temperatures and, conversely, a shift down corresponds to data at lower mean temperatures.

For presentation purposes here, the data sets in Fig. 4 include least square linear fits. The fits for the data sets are generally parallel but slightly shifted reflecting linear density dependence and ordered, from low to high, as follows: 1450a, 1450, 1450d (which are nearly identical), 1450c, and 1450b. The differences in the fits correspond, for the most part, to the bulk density range for each SRM material lot (Fig. 3). For example, the bulk densities of 1450a (60 kg·m^−3^ to 140 kg·m^−3^) and 1450c (150 kg·m^−3^ to 165 kg·m^−3^) are at the low and high ends, respectively, of the density range illustrated in Fig. 3.

Whereas, the small differences in fits for 1450a, 1450d, 1450, and 1450c(II) can be attributed to changes in bulk density, the upward shift in 1450b(II) cannot be attributed entirely to density (nominal value about 130 kg·m^−3^ in Fig. 3). Hust [16, p. 16] also notes that “the reason lot 80/81 [i.e., 1450b] differs from the other lots is not clearly understood.” He does note, however, that the “phenolic resin content of lot 80/81 is lower than other SRM lots: about 14 % by weight compared to 20 % by weight.” These differences are explored further in Sec. 4.2.2.

### 3.5 Certificate Equations

Analysis of the thermal conductivity measurements for each SRM for final reporting purposes requires regression fitting of a model. For SRMs 1450–1450d, the certified properties of interest are thermal resistance, thermal conductivity, and also, for 1450d, bulk density. For a given material lot, the first two properties are characterized as explicit functions of bulk density and mean temperature. Over the past 36 years, different models have been developed for each lot depending on the ranges of bulk density and measured temperature used for each lot. The model for the thermal conductivity measurement data for 1450b [16], given in Eq. (1), represents the most general certification model used for thermal conductivity (λ), in W·m^−1^·K^−1^.

$$\lambda (\rho ,T)=a_{0}+a_{1}\rho +a_{2}T+a_{3}T^{3}+a_{4}e^{-{\left(\frac{T-180}{75}\right)}^{2}}$$ (1)

The parameters ρ and *T* represent bulk density (kg·m^−3^) and temperature (K), respectively, and *a_i_* (*i* = 0, 1, 2, 3, 4) represent the regression coefficients. The Gaussian function (associated with *a*_4_) has constant coefficients 180 and 75 representing the symmetric peak center and width, respectively. The analyses of the other SRMs (1450, 1450a, 1450c, and 1450d) have all used some variation of Eq. (1), with terms included or omitted.

Table 3 summarizes the number of measurements and specimen pairs, major physical variables, and best-fitting model functional form for the thermal characterization of SRMs 1450–1450d. It is immediately evident from Table 3 that the number of measurements and specimen pairs, the ranges of ρ and *T*, and the corresponding model functional forms across lots can be quite different. These differences are due mostly to the historical development and progression of the thermal insulation SRM program.

**Fig. 5:**
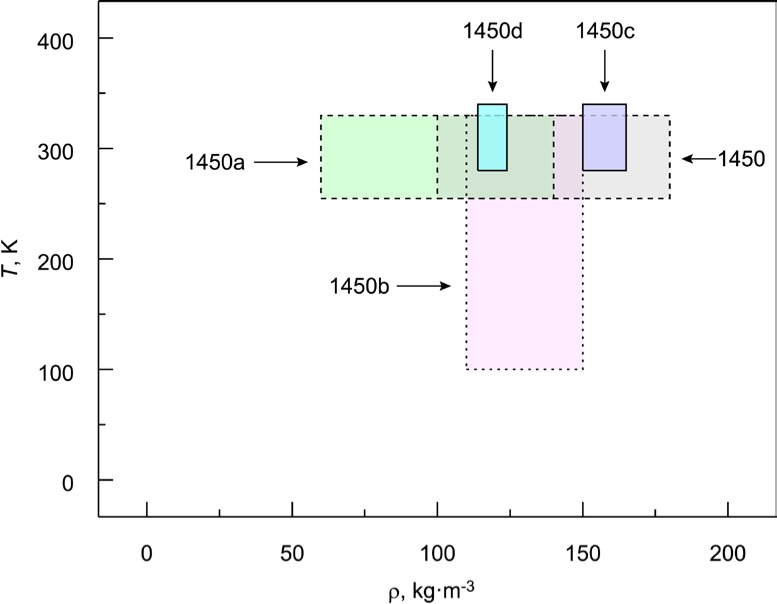
Domain of bulk density (ρ) and temperature (*T*) for SRM 1450 and renewals (data from Table 3).

The cumulative totals for the number of measurements and specimen pairs are 386 and 195, respectively, which is indicative of the significance of this SRM measurement program covering the past 35 years. For SRMs 1450–1450a, the relatively high numbers of measurements and specimen pairs in Table 3 are due to the requirement of an individual measurement for each specimen as part of the preceding calibration program. The measurement number for SRM 1450b(II) is large due to the low-temperature characterization (down to 100 K). Of interest is the introduction of an experimental design plan for 1450c and 1450d that required a balanced number of measurements for each specimen pair. The experimental design for these SRMs was optimized for the efficient consideration of independent sets of measurements over the given ranges of ρ and *T*. Although there is a large variation in ρ and *T* from lot to lot (Table 3), the ranges for the five lots have considerable overlap as shown in Fig. 5.

**Fig. 6:**
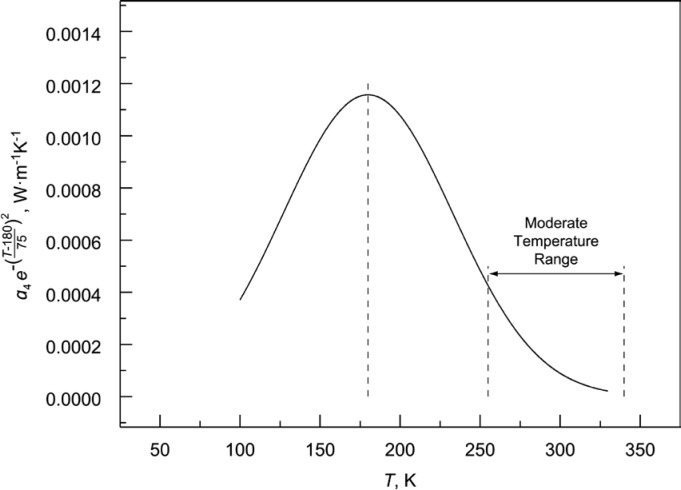
Effect of exponential term in Eq. (1) from 100 K to 330 K, centered on 180 K.

Table 4 summarizes the values for the regression coefficients *a_i_* from Eq. (1) taken directly from the SRM Certificates^5^ for each 1450 lot. Careful inspection of Table 4 reveals the following observations and trends. Values of *a*_1_, which represent the bulk density effect, tend to be smaller for lower range values of ρ and larger for higher range values of ρ given in Table 3. For 1450d, *a*_1_ is zero because most data conform to a particular nominal value of ρ with a small variation in range. Values of *a*_2_, across lots 1450b, 1450c, and 1450d, represent similar slopes of approximately 0.0001 W·m^−1^·K^−1^ per K, reflecting the universal strongly dominant fixed linear relationship between thermal conductivity and temperature for this class of materials, and *T*, ρ ranges (Fig. 4). As might be expected, the effect of *a*_3_ is smaller when *a*_2_ is non-zero (1450b). In the case of 1450b(II), the product of *a*_4_ and the exponential temperature term is a Gaussian-type model that is intended to fit a peak in the thermal conductivity data. The Gaussian model in Table 4 is centered on 180 K and diminishes substantially (due to the peak width parameter value of 75 K) as *T* approaches 100 K or 300 K as illustrated in Fig. 6. This effect of this function is described further in Sec. 7.5.

### 3.6 Certificate Uncertainties

Table 5 summarizes the certified quantity, certification format, stated uncertainties, and coverage factors given in the Certificates for SRMs 1450–1450d. For 1450, 1450a, 1450b and 1450c, certified values of thermal resistance (*R*_0_) were provided in tabular format for a nominal 25.4 mm thick specimen as a function of ρ and *T*. After discussions with thermal insulation SRM customers, the format and certified quantities for the 1450d Certificate were changed. In 2009, the table of values for *R*_0_ was replaced by a thermal conductivity equation λ(*T*) and individual, SRM unit-specific, certified values for ρ.

It is important to state that the uncertainties provided for 1450, 1450a, and 1450b preceded adoption of the NIST Uncertainty Policy in 1992 (Sec. 4.1.2). Reasonable estimates for their respective coverage factors were deduced by the authors based on information provided for the regression analyses in their respective certificates. It is difficult to compare the uncertainties across all 1450 designations because of changes in the uncertainty policy in 1992. For example, it is almost certainly not the case that all the same uncertainty sources were considered across all SRM designations. However, it is interesting to note that the expanded uncertainties have decreased from about 2 % for the early SRMs to 1 % for the most recent designation, 1450d. Additional comments on the uncertainties appear in Sec. 8.

## 4. Evolution of SRM 1450 and Renewals

Section 4 describes the general factors that have affected the development of SRM 1450 and subsequent renewals during the preceding 36 years. These factors involve external issues, institutional policies (described briefly), and specific factors related to the technical information documented in Tables 2 through 4. The discussion on the technical factors addresses the following questions:

1. What are the major technical factors that have affected the 1450 renewals?
2. How have changes in these factors, if any, affected the 1450 renewals?

### 4.1 External Influences and Institutional Policies

In response to specific international agreements and standards, as well as internal policy changes, the SRM certification process at NIST has become more formalized and standardized. These trends have significantly affected the development of subsequent renewals 1450c and 1450d. A brief timeline of these events is given.

#### 4.1.1 Committee on Reference Materials (1975)

In 1975, the Committee on Reference Materials (REMCO) was established [28] by the International Organization of Standardization (ISO). As part of its mission, the committee developed a series of ISO Guides including terminology [29], certificate contents [30], general requirements for the competence of reference material producers [31], and statistical approaches [32].

#### 4.1.2 NIST Uncertainty Policy (1992)

In October 1992, NIST adopted a new policy on the expression of measurement uncertainty [33] consistent with international guidelines given in the Guide to the Expression of Uncertainty in Measurement [34], commonly known as the “GUM”. The Statistical Engineering Division at NIST was tasked with the implementation of the NIST Policy with respect to the uncertainty assessment for SRMs.

#### 4.1.3 NIST SRM Terms and Practices (2000)

In January 2000, the NIST Analytical Chemistry Division and the Standard Reference Materials Program jointly published [35] “Definitions of Terms and Modes Used at NIST for Value-Assignment of Reference Materials for Chemical Measurements.” These terms and modes for value assignment and/or certification currently apply to all SRMs developed at NIST.

#### 4.1.4 NIST Quality System (2003)

In October 2003, NIST implemented an institutional quality system for measurement services and reference materials in response to the International Committee for Weights and Measures (CIPM) Mutual Recognition Arrangement (MRA) [36]. The NIST Quality System [37] commits to ensuring that the internal quality system shall, to the extent possible, conform to the international standard ISO/IEC 17025 [38] and the relevant requirements of ISO Guide 34 [31] as they apply to Standard Reference Materials.

### 4.2 Significant Technical Factors

The significant technical factors that affect the determination of the experimental thermal conductivity (λ_exp_) involve the following: 1) laboratory facility (includes operator); 2) material factor (primarily the bulk density effect); 3) experimental and statistical analytic procedures; 4) equipment; 5) measurement equation; and, 6) environment. Historical changes in these technical factors are discussed in Sec. 4.2.1–4.2.6.

#### 4.2.1 Facilities

Over the past 56 years (20 years for the calibration program, 36 years for SRM 1450 and subsequent renewals), the NBS/NIST laboratory facilities have evolved and the researcher staff involved in the work has undergone transition. During this time period, four different guarded-hot-plate apparatus, operated by different personnel, at NBS/NIST were utilized. Their diverse built-in ranges of operation, in part, are responsible for the different temperature ranges utilized for the thermal characterizations of the particular SRM lots (Table 3). Table 6 summarizes the main equipment characteristics of the guarded-hot-plate apparatus used in the production of 1450 and renewals.

The measurement data for Lots 1959, 1970, and SRMs 1450, 1450a, 1450b (Gaithersburg) were manually collected and hand recorded. During tests, a precision potentiometer was used for accurate measurement of low direct-current (DC) voltages. In general, the potentiometer provided three ranges for measurement of voltage levels. The low range (0 V to 0.016 V) was measured with a resolution of 0.01 μV. Thermocouple voltages were referenced to a cold junction – ice bottle. Later facilities were modernized so that automated data collection was used for SRMs 1450b (Boulder), 1450c, and 1450d. The main benefit was the calculation of final results from observed data by means of a desk-top computer that resulted in increased precision and reduced measurement time. The most recently constructed 1016 mm guarded-hot-plate apparatus utilized precision resistance thermometers in place of thermocouples for temperature measurements.

#### 4.2.2 Material Factor

The key material factor that has been documented by NIST for each SRM lot – in fact, for each test specimen (Table 3) and, for 1450d, each SRM unit (approximately 400 total) – is the macroscopic property bulk density. The high density characteristic of the SRM material is achieved by molding the raw material under heat and compression into board form (Sec. 3.1). The bulk density (ρ), which includes the glass fibers, binder, and interstitial void volume, is defined in Eq. (2) simply as the specimen mass (*m*) divided by the total volume (*V*) of the test specimen.

$$\rho =\frac{m}{V}=\frac{m}{L_{1}\times L_{2}\times L_{3}}$$ (2)

For specimens having a rectangular prism geometry, *V* is equal to the product of the overall dimensions, *L_i_* (*i* = 1, 2, 3). The specimen mass, *m*, is determined gravimetrically generally after oven drying near 100 °C and the specimen dimensions, *L_i_*, by a precision scale and/or digital height gages [17 and 18, respectively].

It is observed in the summary data of Table 3 that the regions and ranges for ρ have changed across successive lots (Sec. 3.5). For the most recent renewals, the nominal target values of 160 kg·m^−3^ and 128 kg·m^−3^ for 1450c [17] and 1450d [18], respectively, were based on industry guidance (Sec. 4.2.3.3–4.2.3.4). The range reductions in ρ for these lots are attributed to the fact that, in the procurement process, NIST purposely specified: 1) the material shall be obtained from one fabrication run; and, 2) the acceptance limit for bulk density shall be no more than 10 % (for 1450d). In contrast, the materials for the early SRM lots, notably 1450, 1450a, and 1450b, were obtained by procurement or donation, presumably without specific requirements for bulk density imposed by NIST. It should be added, that the first two lots (1450 and 1450a) were initially obtained as part of a calibration program and, thus, specific range requirements for the bulk density were not anticipated for later use in the SRM program.

The graphical overview of the data (Sec. 3.4) revealed: 1) the weak dependency of λ on ρ (Fig. 3) for a given value of *T*; and, 2) that differences in λ from lot to lot cannot be explained entirely by a bulk density effect (Fig. 4). Thus, it is somewhat unfortunate that other material parameters explored for specific lots (Sec. 3.3) have not been more systematically investigated. Even so, auxiliary data and facts documented for SRMs 1450–1450c [7, 16, 17] are useful as descriptors of these materials. Micrographs [7, 17] show the complexity of the fiber arrangement, variability of fiber diameters and fiber contacts, and application of binder. For SRM 1450c, the range of glass fiber diameters was expected to be between 6 μm to 8 μm [17]. (The micrographs showed slightly larger diameters due to the presence of binder.) The glass fibers for 1450c were documented [17] as an alkali-alkaline alumino-borosilicate glass bonded with a phenyl-formaldehyde binder. Reference [22] records a commercial glass composition for “E-glass,” commonly used for commercial applications of fibrous glass (by mass): SiO_2_, 52.9 %; Al_2_O_3_, 14.5 %; B_2_O_3_, 9.2 %; CaO, 17.4%; MgO, 4.4 %; and K_2_O, 1.0 %.

The upper temperature limit of the binder content was investigated for multiple small specimens by thermogravimetric analysis (TGA) [17, 27]. At temperatures above 200 °C, the mass loss was appreciable [17] most likely due to chemical breakdown of the binder (and other organics). Above 600 °C, all organic matter had burned away, and only the glass fibers remained [17]. The fractional mass loss (in %) for the binder ranged from 17.8 to 18.7 for SRM 1450b [27] (3 specimens) and from 19.5 to 30.7 for SRM 1450c [17] (6 specimens). The burn out for larger specimens of 1450b was 14 % [16] and 16.4 % [27]. The TGA data for SRMs 1450b and 1450c support observations by Hust [16] that the phenolic resin content was lower for the 1450b lot.

#### 4.2.3 SRM Production Procedure

Perhaps more than any other documented technical factor, the process for the production of the 1450 renewals has undergone considerable progress. As part of the institutional changes in SRM policies (Sec. 4.1), the process for statistical characterization of the 1450 lots has matured substantially. A major improvement was introduced for 1450c with the development of a certification test plan (Fig. 2) that included a careful experimental design approach for measurement and associated analyses. The test plans for 1450 and each SRM renewal are briefly summarized.

##### 4.2.3.1 1450 and 1450a

As mentioned in Sec. 2, the thermal conductivity data for 1450 and 1450a were originally acquired over several years as part of a calibration program established in the 1950s. The data, which were recorded in NBS logbooks, were compiled and subsequently transcribed for computer analysis (see Addendum in Appendix J for full historical background). The certification of SRMs 1450 and 1450a, which were issued in 1978 and 1979, respectively, were based on the statistical analyses of data from material lots that were originally obtained in 1961 and 1958, respectively. After-the-fact statistical analyses [7] were used to demonstrate “that the material is sufficiently homogeneous and possesses the necessary thermal stability for characterization as a Standard Reference Material.”

##### 4.2.3.2 1450b

The depletion of the remaining material in SRMs 1450 and 1450a due to limited stockpiles was fairly rapid and two new lots, designated 1980 and 1981, were acquired for the 1450b renewal [16]. The sampling plan is not explicitly described in the available literature but was presumably based on a random sampling of Lots 1980 and 1981 for the selection of specimens. Among the thermal insulation standard reference materials, 1450b(II) is uniquely heterogeneous in origin for two reasons. The SRM was based on data obtained from the aggregation of two lots of material that were considered to be “indistinguishable,” [16] with the addition of three sets of data obtained from NBS guarded-hot-plate laboratory facilities in Gaithersburg and Boulder (Appendix G).

##### 4.2.3.3 1450c

At the onset of the renewal process for 1450c, the NIST Standard Reference Materials Program distributed a questionnaire to participants of the U.S. thermal insulation industry to confirm interest in continuing the renewal and to request input for renewal material parameters [17]. The results of the questionnaire corroborated continued interest in the SRM and also provided information on desired size (610 mm by 610 mm), thickness (25.4 mm), bulk density, and temperature range, among other parameters, of the SRM unit. The development of 1450c also introduced a carefully structured plan centered on a full factorial 3×5 experimental design for two variables (ρ and *T*, respectively) based on an assumed underlying bilinear model from the previous renewals. The plan required the following items:

1. 100 % sampling of material lot for bulk density (130 large boards later cut to final dimensions by the NIST Standard Reference Materials Program);
2. ordered sampling at three levels of the material lot for the balanced selection of 15 pairs of test specimens: five low-, five mid-, and five high-ρ pairs; and,
3. guarded-hot-plate tests of each specimen pair using a randomized test sequence conducted over 58 days.

In 2010, the Certificate for SRM 1450c(II) was revised with the following notice: “This revision includes a change in regression parameters for the thermal conductivity model, correction of certified values, and updates of the certificate to current NIST standards.”

##### 4.2.3.4 1450d

For 1450d, the certification plan originally developed for 1450c was refined and formalized as illustrated in Fig. 2. During the planning stage, NIST collaborated with industry and the ASTM International Committee C16 on Thermal Insulation to define, once again, the parameters of interest to the user communities. As a result, three significant modifications were initiated for the material acquisition process.

1. Industry members requested a bulk density for the renewal more closely aligned with the 1450b lot. As a result, a target density of 128 kg·m^−3^, in contrast to the nominal 160 kg·m^−3^ for 1450c, was approved [43].
2. Industry members also requested that the thermal conductivity measurements be conducted at a temperature difference (Δ*T*) of 25 K, instead of 20 K as was the case for 1450c. The value of 25 K was considered to be more congruent with current industry practices [44].
3. Under the procurement process, NIST stipulated that the material lot was to be manufactured in one run from the same batch of raw material with final acceptance tolerances of 10 % for bulk density and thickness. In addition, NIST requested that the manufacturer provide the SRM units in their final size (nominally 610 mm by 610 mm). This change was designed to mitigate the effect of any post-analysis dimensional resizing of the artifacts. The manufacturers of previous 1450 material lots provided large boards (1200 mm by 1200 mm or other size) that were subsequently cut to final size by NIST. Finally, NIST requested and received quality control charts for the entire three day fabrication process [18] as well as acceptance test results for the raw materials used in fabrication.

The research stage of 1450d was extended by about two years because of the need to identify a new material source. In order to locate a material source, NIST conducted an investigation that examined the thickness, bulk density, and thermal conductivity (not included in Appendix I) variations of two candidate materials [43]. After assessing the technical qualifications of the candidate materials and selecting one, the production of 1450d proceeded as shown in Fig. 2. The thermal conductivity measurements of the 1450d test specimens were completed in 44 days.

#### 4.2.4 Measurement Technique and Equipment

**Fig. 7:**
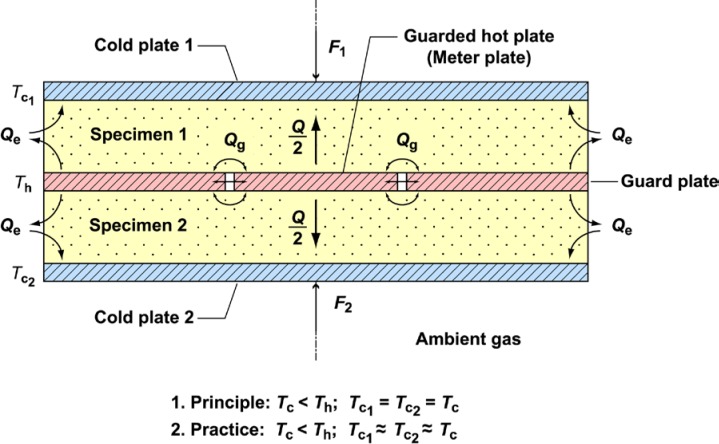
Guarded-hot-plate schematic, double-sided mode of operation – vertical heat flow.

Figure 7 shows the essential features of a guarded-hot-plate apparatus designed for operation near ambient temperature conditions in the double sided mode. The guarded-hot-plate apparatus used at Boulder is similar in principle, although more complex in design and control due to low-temperature operation [41]. The apparatus illustrated in Fig. 7 is cylindrically symmetric about the axis indicated. The plates are horizontal and heat flow (*Q*) is vertical (up/down) through the pair of specimens. The specimen pair, each of which has nearly the same density, size, and thickness, are placed on each surface of the guarded hot plate and clamped securely by the cold plates. The guarded hot plate and the cold plates provide constant-temperature boundary conditions (*T_h_*, and *T_c_*, respectively) to the specimen surfaces. The subscripts “h” and “c” refer to hot and cold surfaces, respectively, and the subscript numbers “1” and “2” are associated with each cold plate.

With proper guarding, lateral heat flows (*Q_g_* and *Q_e_*) are reduced to negligible proportions and, under steady-state conditions, the apparatus effectively provides one-dimensional heat flow (*Q*) normal to the meter area of the specimen pair. For apparatus operating near room temperature, a secondary guard was provided by an enclosed chamber that conditions the ambient air surrounding the plates to a temperature near to the mean specimen temperature, *T_m_* (i.e., average of the surface temperatures of the hot and cold plates in contact with the specimens). The low-temperature guarded-hot-plate apparatus at Boulder utilized an isothermal heated copper shell as a secondary guard [41]. Additional details for low-temperature operation of the apparatus are provided in Ref. [41].

#### 4.2.5 Measurement Equation

Under steady-state conditions, Eq. (3) is the operational definition [45] for the experimental thermal conductivity of the specimen pair (λ_exp_)

$$\lambda_{\exp}=\frac{Q}{A[{\left(\Delta T/L\right)}_{1}+{\left(\Delta T/L\right)}_{2}]}$$ (3)

where *Q* and *A* are the specimen heat flow rate and area through which *Q* passes, respectively. The ratio (Δ*T*/*L*)_1_ is equal to the surface-to-surface temperature difference (*T_h_* − *T_c_*_1_) to the thickness (*L*) for Specimen 1 (Fig. 7). A similar expression is used for Specimen 2.

The thermal transmission properties of heat insulators determined from standard test methods typically include several mechanisms of heat transfer, including conduction, radiation, and possibly convection. For that reason, some experimentalists will include the adjective “apparent” or “experimental” when describing thermal conductivity of thermal insulation. However, for brevity, the term thermal conductivity is used in this paper.

When the temperature differences and the specimen thicknesses are nearly the same, respectively, Eq. (3), reduces to

$$\lambda_{\exp}=\frac{QL_{\mathrm{avg}}}{2A\Delta T_{\mathrm{avg}}}$$ (4)

In the double-sided mode of operation (Fig. 7), the thermal transmission properties correspond to a mean temperature *T_m_* given by Eq. (5).

$$T_{m}=\frac{T_{h}+T_{c}}{2}=\frac{T_{h}+(T_{c1}+T_{c2})/2}{2}$$ (5)

Specific values for *L_avg_* and Δ*T_avg_* for the guarded-hot-plate data are discussed in Sec. 5.2. As noted in Table 3, the values for *T_m_* ranged collectively from 100 K to 340 K (Sec. 3.5).

The determination and expression of measurement uncertainty has evolved (Sec. 4.1.2) along with changes in the laboratory facilities (Sec. 4.2.1). The first documentation of uncertainty propagation for the NBS/NIST guarded-hot-plate apparatus was prepared by Siu [40], followed by Smith [41], Rennex [46], and most recently by Zarr [17–18] under the current NIST uncertainty policy [33]. For the multiplicative expression given in Eq. (4), the relative combined standard uncertainty in λ_exp_ can be expressed as the relative uncertainties associated with each factor combined in quadrature.

$$u_{c,\mathrm{rel}}(\lambda_{\exp})=\frac{u_{c}(\lambda_{\exp})}{\lambda_{\exp}}=\sqrt{{\left(\frac{u(Q)}{Q}\right)}^{2}+{\left(\frac{u(\Delta T_{\mathrm{avg}})}{\Delta T_{\mathrm{avg}}}\right)}^{2}+{\left(\frac{u(L_{\mathrm{avg}})}{L_{\mathrm{avg}}}\right)}^{2}+{\left(\frac{u(A)}{A}\right)}^{2}}$$ (6)

The relative expanded uncertainty, *U_rel_*, is defined in Eq. (7) for a coverage factor of *k* equal to 2. Relative uncertainty values for SRMs 1450–1450d, as specified in their respective certificates, are summarized in Table 5. However, as stated before, it is likely that the same uncertainty sources were not considered in the evaluation of *uc,rel* across all 1450 renewals.

$$U_{\mathrm{rel}}(\lambda_{\exp})=ku_{c,\mathrm{rel}}(\lambda_{\exp})=2u_{c,\mathrm{rel}}(\lambda_{\exp})$$ (7)

#### 4.2.6 Environmental Factors

Environmental factors, which are either controlled or recorded during a measurement, include ambient temperature, *T_a_*, pressure, *p_a_*, and relative humidity. The ambient temperature is controlled, as described in Sec. 4.2.4, and the effect of relative humidity is mitigated by application of either a dehumidification coil [40], dry-air purge [17–18], or dry back-fill gas such as nitrogen [16, 41]. However, due to different elevations above sea level of approximately 152 m and 1629 m [47], local atmospheric pressures at Gaithersburg and Boulder are approximately 100 kPa and 82 kPa, respectively. This difference in ambient pressure, however, has an extremely small effect on the thermal conductivity of fibrous glass board due to its relatively large pore size [7, 16–18, 26]. Briefly, the gas conductivity of a porous solid is dependent on gas pressure when the ratio of the characteristic system length (i.e., pore size) and the mean free path for the gas molecules are dimensionally similar (which is not the case for fibrous glass board). The mean free path length is the average distance a gas molecule travels before collision with another gas molecule.

### 4.3 Technical Factor Summary

A qualitative assessment summary of the technical factors that have affected the development of the 1450 renewals over the past 36 years is given. Factors are ranked by order of effect.

Factor 1) *Procedure*: The procedure for the production of 1450 renewals (Sec. 4.2.3) has progressed substantially since 1978, mostly due to the implementation of a statistically based design plan, resulting in significant improvement in the thermal characterization of the SRM. This improvement is believed to be partly responsible for the uncertainty reduction in the certification values of each SRM lot detailed in Table 5 (see also Factor 3, below). The procedure factor has also been modified, in part, due to external influences (i.e. ISO standardization) and administrative changes in the SRM program at NIST.
Factor 2) *Material*: The key material factor determined for each specimen is the bulk density. In recent years, however, NIST has taken a more proactive approach in specification of the material macro-properties, specifically bulk density and board thickness for the material lot.
Factor 3) *Facilities*: The underlying trend in uncertainty reduction in the certification values of each SRM lot (Table 5) is attributed, in part, to the long-term modernization of the laboratories (see also Factor 1, above).
Factor 4) *Measurement Technique and Environment*: Although the standardized test methods and practices [1, 45] have been periodically updated over the past 36 years, the underlying physics and the resulting measurement technique and measurement equation are unchanged. Environmental factors have been controlled in accordance with standardized test methods.

## 5. Overview of Data Sets

Section 5 gives an overview of the data sets for this analysis. The data sets include not only the SRM data described in Sec. 3 and 4 but also data for similar materials, designated as proto-1450 data and identified by lot numbers originally assigned by the year of acquisition. Individual data sets are represented graphically as a function of bulk density (ρ) or (mean) temperature (*T*). Summary comments are provided for each data set.

### 5.1 Description of Data Sets

Table 7 summarizes the data sets, designated 1 through 11, that are re-examined in this study, including not only the 1450 data sets (4–11, excluding 6) but also proto-1450 material lots (1–3). (Note that data sets 1, 2, 3, and 6 are not included in the graphical overview shown in Figs. 3 and 4.) For this investigation, the data sets are identified by laboratory facility, although in one case, 1450b(II), the data were combined across laboratories in the original analysis. The data for 1450b(I) in Table 1 are not explicitly included in this analysis as a separate data set. These data, however, are included as part of data sets 7 through 9 for 1450b(II). It should also be noted that data sets 3 and 6 were re-measured by Boulder several years after the initial Gaithersburg measurements. The data sets of Table 7 are reproduced in their entirety (with numerical precision as originally presented or as inherited in computer printouts) in Appendix A through Appendix I.

### 5.2 Graphical Presentation of Data Sets

**Fig. 8:**
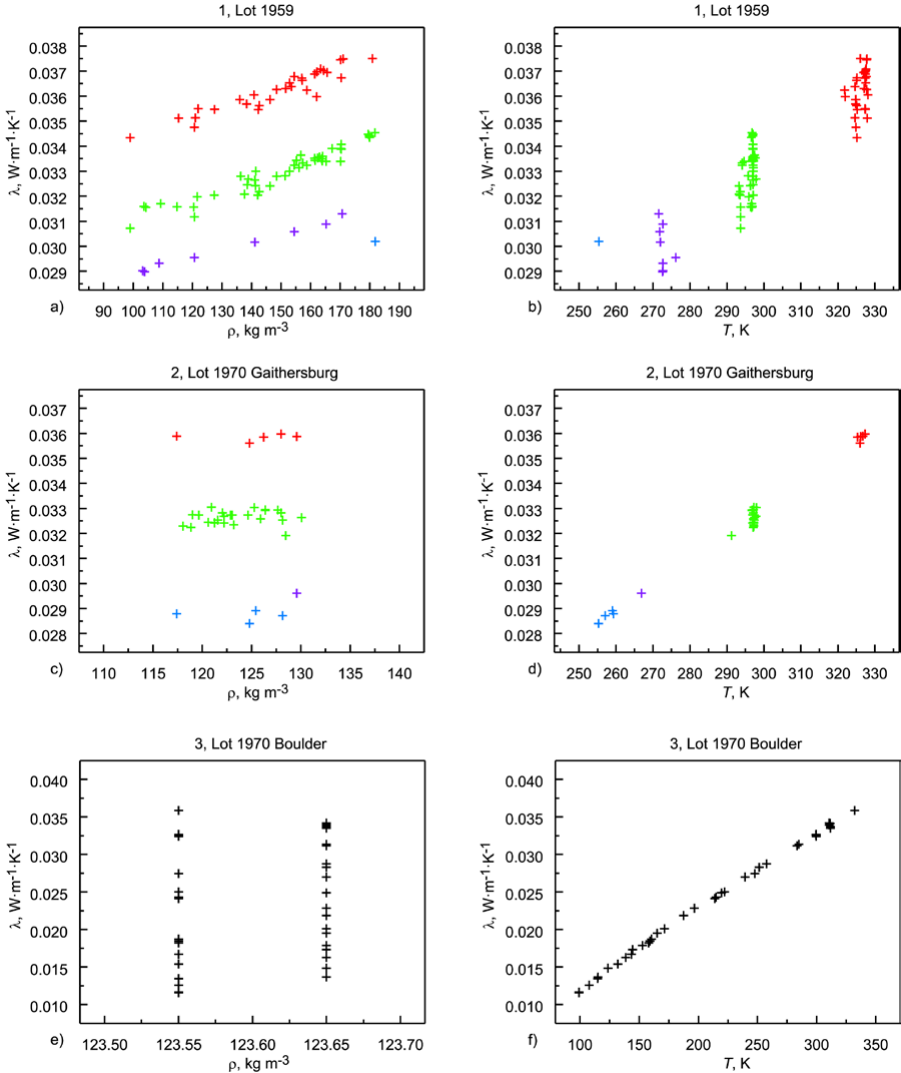

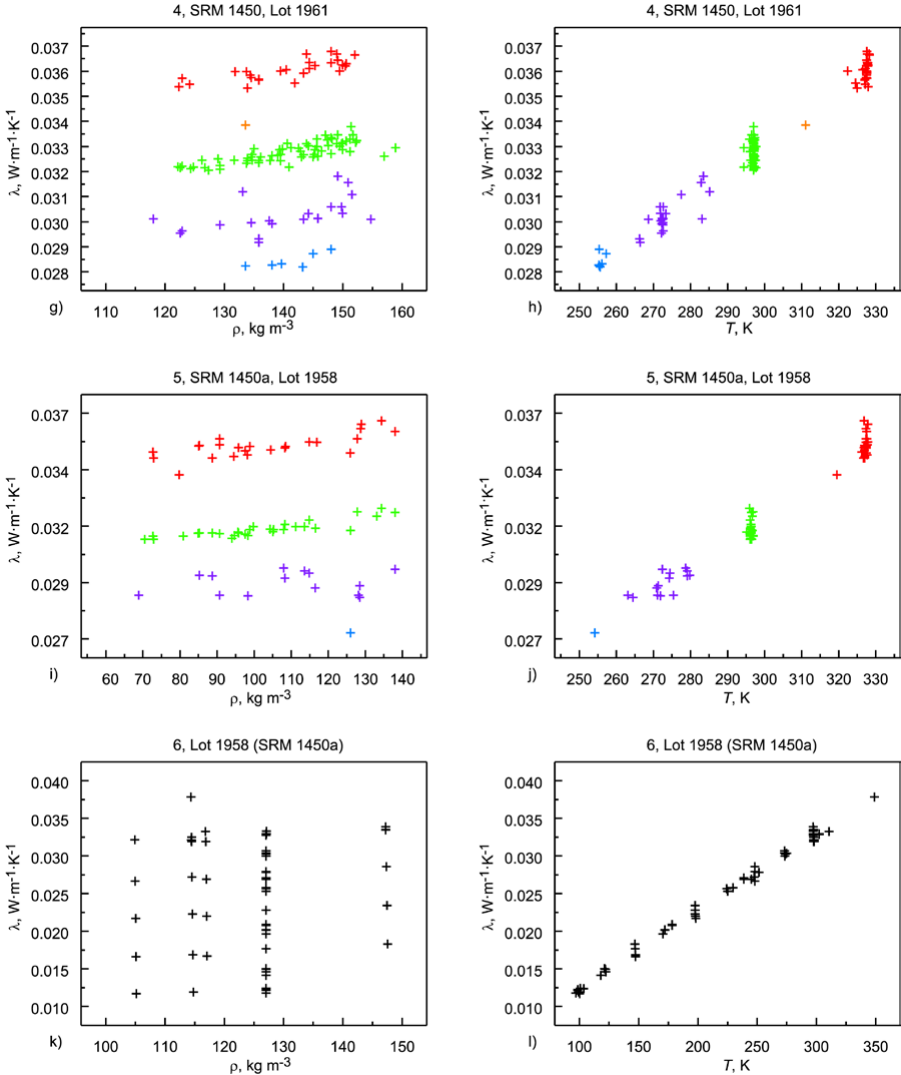

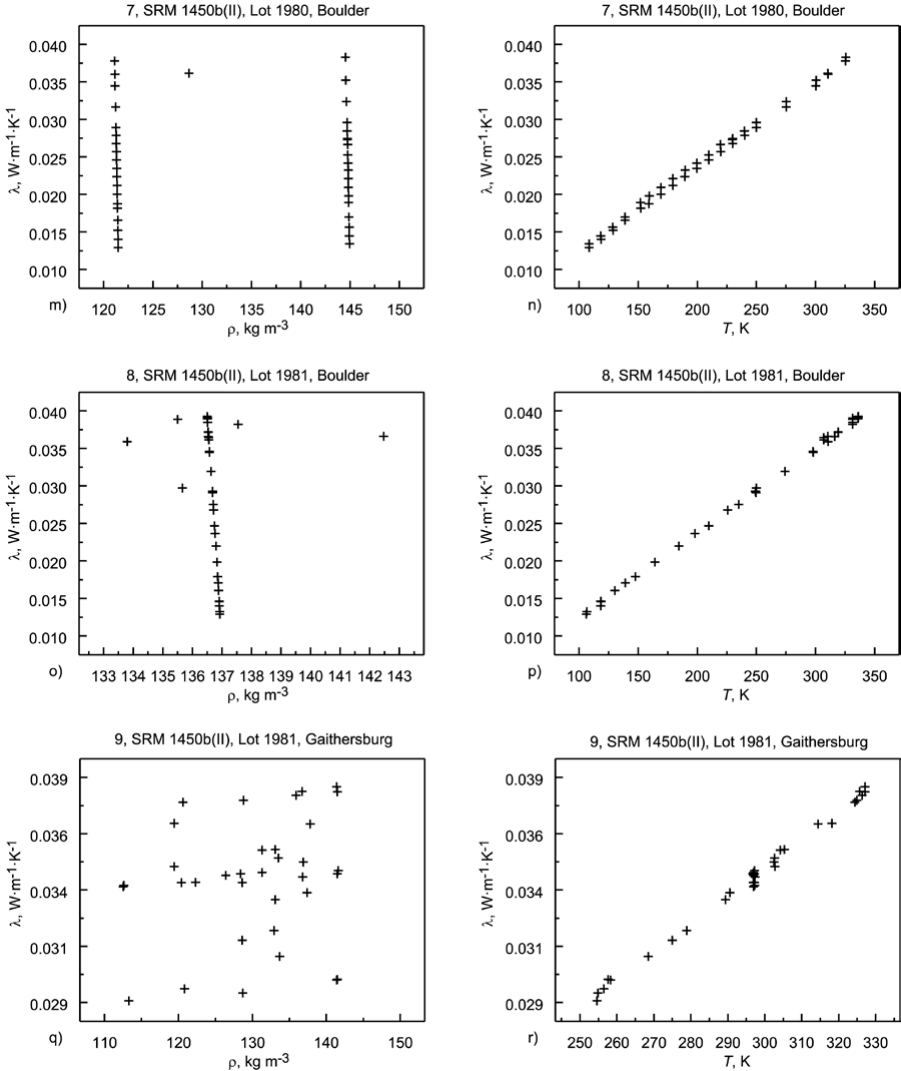

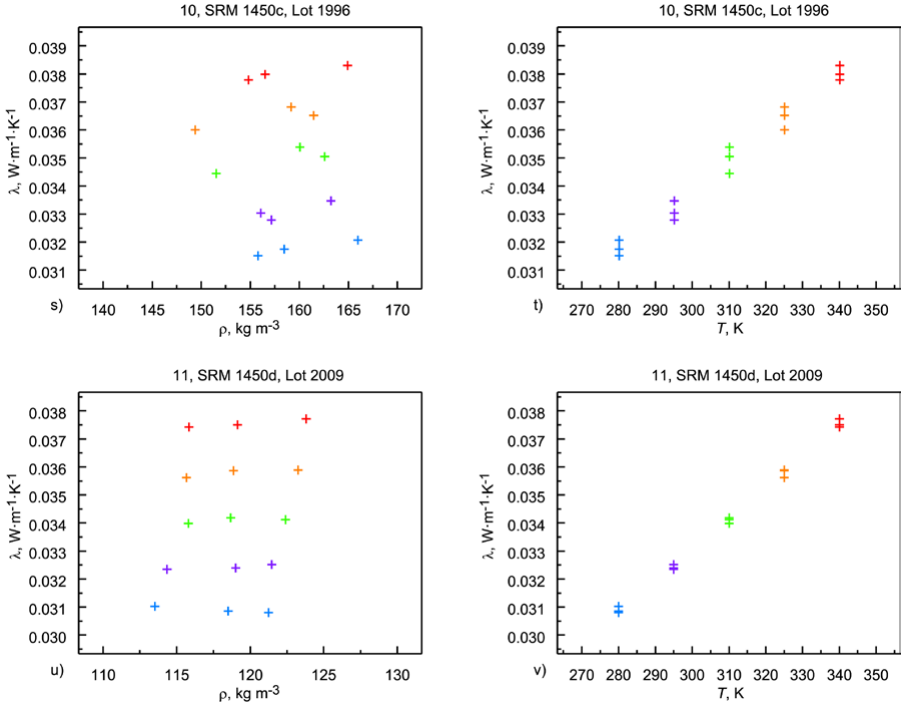
a–f. Graphical presentation of data sets 1, 2, and 3. Color encodes temperature level for plots having discretized temperature data. g–l. Graphical presentation of data sets 4, 5, and 6. Color encodes temperature level for plots having discretized temperature data. m–r. Graphical presentation of data sets 7, 8, and 9. s–v. Graphical presentation of data sets 10 and 11. Color encodes temperature level for plots having discretized temperature data.

The individual data sets in Table 7 are presented graphically in a sequence of multi-plots in Figs. 8a through 8v. Data sets that cluster naturally as a function of temperature are color coded. Data that are continuously distributed across temperature are presented without color coding. Observations from Figs. 8a through 8v are summarized.

1. Data set 1 (Appendix A): Lot 1959 is the only material lot with a nominal board thickness of 13 mm (Ref. [7] and Table A1). It is not known why this particular thickness (13 mm) was not continued in subsequent SRM development. The data are derived from multiple calibration runs across several years. As shown in Fig. 8a, Lot 1959 has one of the widest ranges of bulk density (100 kg·m^−3^ to 180 kg·m^−3^) and the temperature values cluster into three distinct groups and one data point (Fig. 8b).
2. Data set 2 (Appendix B): As was the case for data set 1, the data for Lot 1970 Gaithersburg are derived from multiple calibration runs across several years. The data are relatively restricted in bulk density with a nominal value near 125 kg·m^−3^ (Fig. 8c). There is one temperature cluster near 297 K and two smaller sets at 255 K and 325 K (Fig. 8d).
3. Data set 3 (Appendix C): Lot 1970 Boulder comes from the same material lot as data set 2 but was measured several years afterward. There is essentially one nominal bulk density, with two subclusters at 123.55 kg·m^−3^ and 123.65 kg·m^−3^ (Fig. 8e). The data are spread continuously over a wide temperature range from 100 K to 330 K (Fig. 8f). Careful inspection of the temperature plot (Fig. 8f) reveals that the data are linear at higher temperatures but exhibit mild departure from linearity near 160 K. The temperature differences (Δ*T*) range from 12.5 K to 124 K and the median value is 26 K (Table C1, Appendix C).
4. Data set 4 (Appendix D): Standard Reference Material 1450 (Lot 1961) has a wide range of bulk densities (115 kg·m^−3^ to 160 kg·m^−3^) with most above the 120 kg·m^−3^ region (Fig. 8g). There are multiple temperature clusters, with principal clusters near 270 K, 297 K, and 330 K (Fig. 8h). As is the case for data sets 1 and 2, the data are derived from multiple calibration runs across several years.
5. Data set 5 (Appendix E): Standard Reference Material 1450a (Lot 1958) also exhibits a wide range of bulk densities (70 kg·m^−3^ to 140 kg·m^−3^) with most data points less than 120 kg·m^−3^ (Fig. 8i). There are three clusters in the temperature data near 270 K, 297 K, and 330 K (Fig. 8j). The data are derived from multiple calibration runs across several years.
6. It should be noted that the temperature clusters observed in data sets 1, 2, 4, and 5 are the result of the temperature conditions requested by the customer participants under the NBS Calibration Program. The particular test temperatures for customers are specified in the Addendum (Appendix J).
7. Data set 6 (Appendix F): Lot 1958 Boulder is from the same material lot as data set 5 but was measured several years afterward. As shown in Fig. 8k, the bulk density is clustered at four levels and covers a more restricted range than data set 5 (105 kg·m^−3^ to 147 kg·m^−3^). The thermal conductivity data are essentially continuous in temperature over a range of 100 K to 330 K (Fig. 8l). The Δ*T* ranges from 10.5 K to 38.5 K and the average value is 23 K (Table F1, Appendix F).
8. Data set 7 (Appendix G, Subset 1): This data set for Lot 1980 Boulder is incorporated as part of 1450b(II) and has three levels of bulk density (121 kg·m^−3^ to 145 kg·m^−3^), with one level represented by only one data point (Fig. 8m). The data are essentially continuous over a temperature range of 100 K to 330 K (Fig. 8n). Careful inspection of the temperature data indicates a gentle undulation in the data that peaks near 180 K (Fig. 8n). The Δ*T* ranges from 24.2 K to 31.8 K and the average value is 25 K (Table G1).
9. Data set 8 (Appendix G, Subset 2): This data set from Lot 1981 Boulder has a limited density representation, centered on 137 kg·m^−3^ (Fig. 8o). The data are continuously represented over a temperature range of 100 K to 330 K (Fig. 8p). The Δ*T* ranges from 20.9 K to 38.9 K with average value of 25 K (Table G1).
10. Data set 9 (Appendix G, Subset 3): This data set from Lot 1981 Gaithersburg has a continuous distribution of ρ (112 kg·m^−3^ to 142 kg·m^−3^) as shown in Fig. 8q. The data are essentially continuous across the temperature range of 255 K to 330 K (Fig. 8r). The Δ*T* ranges from 19.9 K to 24.4 K with average value of 23 K (Table G1).
11. Data set 10 (Appendix H): Standard Reference Material 1450c(II) (from Lot 1996) represents a discretized (by design) underlying continuous distribution of bulk density from 150 kg·m^−3^ to 165 kg·m^−3^ (Fig. 8s). The temperature is uniformly distributed across 280 K to 340 K in five cluster levels (by design) (Fig. 8t). Each data point represents a different pair of specimens (by design).
12. Data set 11 (Appendix I): Standard Reference Material 1450d (from Lot 2009) has three levels bulk density (by design) distributed tightly across a range of 114 kg·m^−3^ to 124 kg·m^−3^ (Fig. 8u). The temperature is uniformly distributed across 280 K to 340 K with measurements concentrated at five cluster levels (by design) (Fig. 8v). Each data point represents a pair of specimens (again by design).

## 6. Analysis

Using the most general model for λ (ρ, *T*) of Eq. (1) as an end point, the following nested hierarchy of models, identified as Models 0 through 6, were systematically tested against data sets 1 through 11 (Table 7). With the exception of Models 0 and 1, all models are two-parameter models in ρ and *T* and include a constant intercept term, *a*_0_. The reference Model 0, fitting to the mean value of λ, is only included to provide baseline values for certain diagnostic statistics described later. For two sets of data (3, 7), Models 5a and 6a were used to examine the utility of the exponential term coefficients *b* and *c*. Note that Model 6 is Eq. (1).

$$\mathrm{Model}\;0:\;\lambda =\bar{\lambda }$$ (8)

$$\mathrm{Model}\;1:\;\lambda =a_{2}T$$ (9)

$$\mathrm{Model}\;2:\;\lambda =a_{0}+a_{2}T$$ (10)

$$\mathrm{Model}\;3:\;\lambda =a_{0}+a_{1}\rho +a_{2}T$$ (11)

$$\mathrm{Model}\;4:\;\lambda =a_{0}+a_{1}\rho +a_{2}T+a_{3}T^{3}$$ (12)

$$\mathrm{Model}\;5:\;\lambda =a_{0}+a_{1}\rho +a_{2}T+a_{4}e^{-{\left(\frac{T-180}{75}\right)}^{2}}$$ (13)

$$\mathrm{Model}\;5a:\;\lambda =a_{0}+a_{1}\rho +a_{2}T+a_{4}e^{-{\left(\frac{T-b}{c}\right)}^{2}}$$ (14)

$$\mathrm{Model}\;6:\;\lambda =a_{0}+a_{1}\rho +a_{2}T+a_{3}T^{3}+a_{4}e^{-{\left(\frac{T-180}{75}\right)}^{2}}$$ (15)

$$\mathrm{Model}\;6a:\;\lambda =a_{0}+a_{1}\rho +a_{2}T+a_{3}T^{3}+a_{4}e^{-{\left(\frac{T-b}{c}\right)}^{2}}$$ (16)

It should also be noted that all models used are multilinear, that is, linear and/or nonlinear component terms are always combined additively. The hierarchy of successively more complex models, from linear in *T* to the comprehensive model of Eq. (16), represents a nested set of models. That is, each model is a linear submodel of the next successively more complex submodel. This observation permits meaningful goodness-of-fit comparisons of the models on the basis of model bias as well as variance.

### 6.1 Graphical Techniques

When a model is fit to a given data set, the quality of the fit can judged graphically as well as analytically. The graphs described in this section are demonstrated with data sets 10 and 11 from 1450c and 1450d, respectively, which were specifically selected because both were the result of similar experimental designs (Sec. 4.2.3). These particular data sets also highlight a recurrent issue for the data studied here, that is, the inclusion (or non-inclusion) of a (linear) term in bulk density (ρ). Data set 7 (1450b, Boulder) is also included in Sec. 6.1.3 to demonstrate the necessity of higher order *T* terms.

#### 6.1.1 Data Layout Plots

**Fig. 9:**
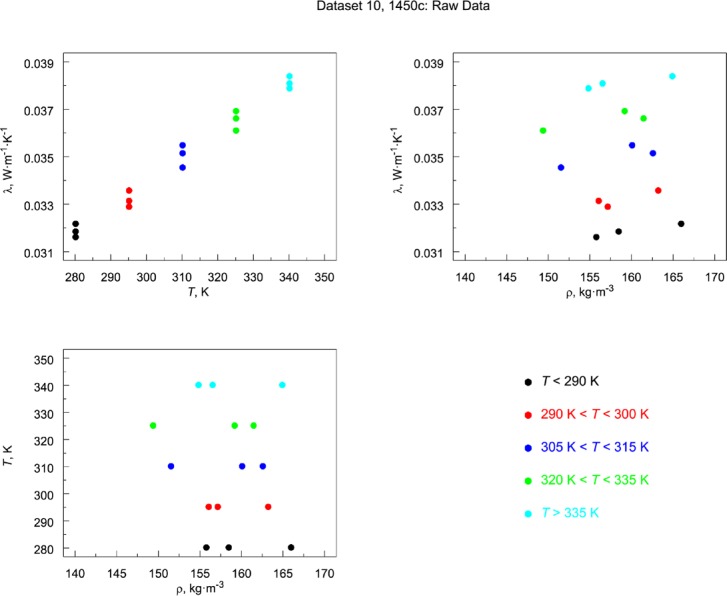
Data layout plot for data set 10, 1450c.

**Fig. 10:**
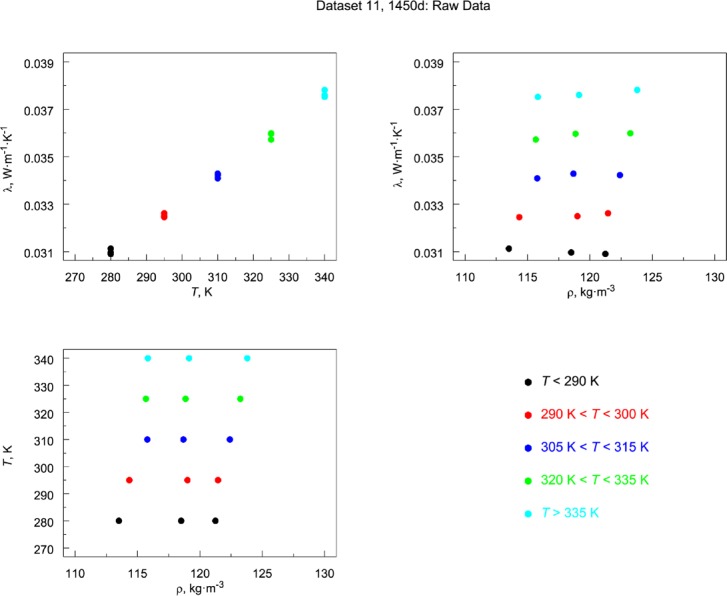
Data layout plot for data set 11, 1450d.

A first step in almost any modeling is to visualize the data as illustrated primarily in Figs. 3 and 4. If the data being studied are multi-dimensional and highly complex, they can often be broken down into component pieces and graphed. For the typical equation λ = *f* (ρ, *T*), with some higher order temperature terms being considered, appropriate layout plots are graphics that explore the dependency of λ on ρ and *T*, making use of multiple plots, multiple frames in a single plot, coloring, etc. The plots shown in Figs. 9 and 10 for 1450c and 1450d, respectively, illustrate clearly the expected strong linear dependence of λ on *T*. Thermal conductivity is plotted versus *T* and ρ, respectively, with the data points color coded by temperature range. In the third frame, independent variables (*T* and ρ) are plotted. Neither plot, however, makes an immediately apparent argument for inclusion of ρ in the model.

#### 6.1.2 Partial Residual Plots

When there is a single independent variable, we can graphically assess the nature of the relationship by plotting the response variable against the independent variable. When there is more than one independent variable, we can plot the response variable against each of the independent variables. However, this approach has the limitation that the plot of the response variable against a specific independent variable does not take into account the effect of the other independent variables in the model.

The partial residual plot [48] attempts to show whether there is a relationship between the response variable and a specific independent variable, taking into account other potential independent variables in the model. One limitation of the partial residual plot is that if the independent variable being plotted is highly correlated with any of the other independent variables being tested, the resulting plot can be misleading. For that reason, we restrict the partial residual plots to the temperature and density terms since the cubic temperature and exponential temperature terms correlate with the linear temperature term. For a given independent variable, *x*(*i*), the partial residual plot is formed as

$$\mathrm{RES}+\hat{\beta }(i)\times x(i)\;\text{versus}\;x(i)$$ (17)

where:

RES = the residuals from the full model;
$\hat{\beta }(i)$ = the regression coefficient from the *i*_th_ variable in the full model
*x*(*i*) = the *i*_th_ independent variable

**Fig. 11:**
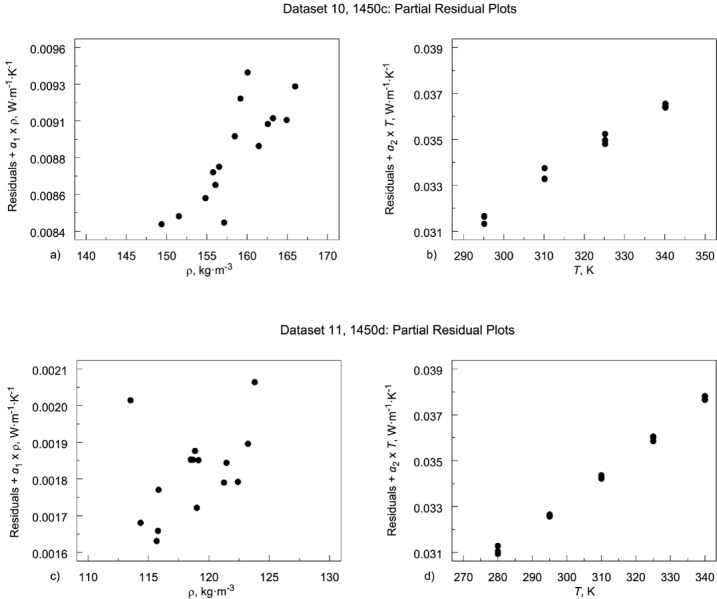
Partial residual plots for data sets 10 and 11, 1450c and 1450d, respectively.

Any reasonably clear structure (e.g., linear, exponential, oscillatory) in a variable’s partial plot is indicative of the need to include that variable in the model. The partial residual plots for the 1450c data set show a clear case for including variables for both ρ (Fig. 11a) and *T* (Fig. 11b). On the other hand, the partial residual plots for the 1450d data set show no clear linear relationship for ρ (Fig. 11c), only *T* (Fig. 11d).

#### 6.1.3 Residual Factor Plots for Assessment of Model Adequacy

A standard approach to assessing model adequacy is to plot residuals from the fitted model against model variables and/or factors that could influence response variable behavior. Residuals are typically plotted against each variable that enters, or could potentially enter, into the modeling. In examining the plots, one checks for structure: clumping, discretization, linearity, sinusoidicity, exponentiality, or any locally parameterizable structure. Residuals are model-fitted predicted values subtracted from corresponding empirical response values. As such, they present a detailed picture of the inadequacies of a fitted model. As a diagnostic tool, the plots also serve to confirm the adequacy of a given fit to a given model. Plotting these measures of model inadequacy against variables and factors, however, does represent a potentially constructive step in that it may suggest approaches to improving the model being tested with factors or terms that decrease or eliminate such structural inadequacies.

**Fig. 12:**
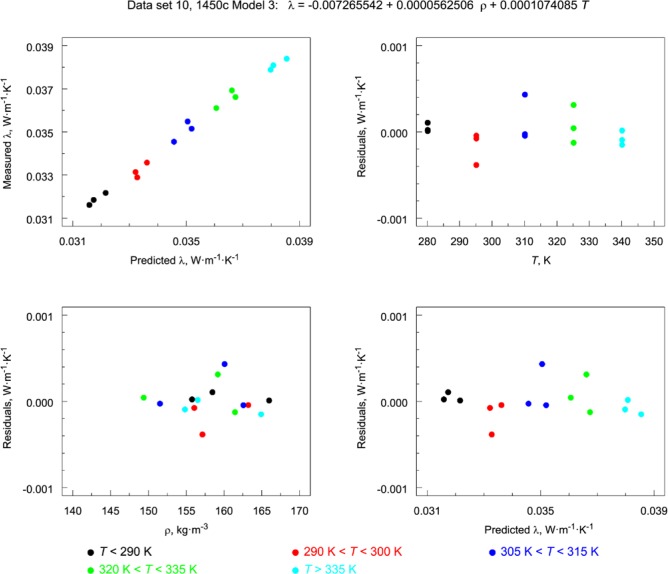
Residual factor plot for data set 10 (1450c).

**Fig. 13:**
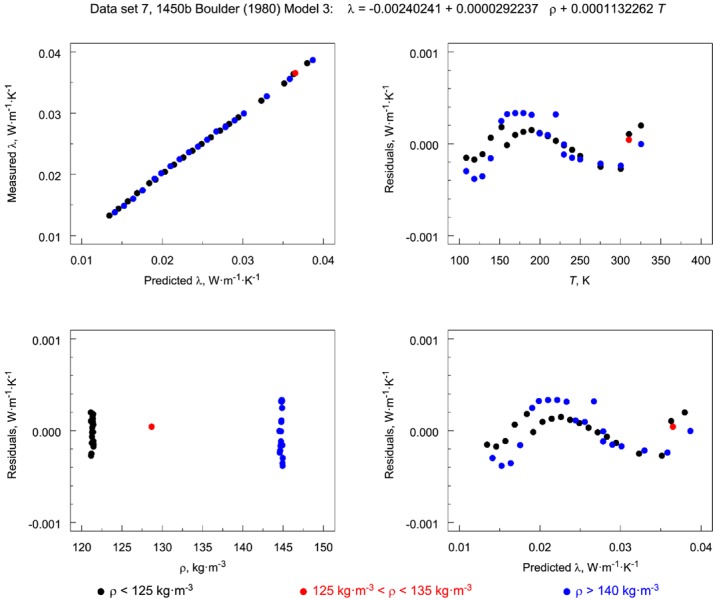
Residual factor plot for data set 7 (1450b, Boulder Lot 1980).

Figures 12 and 13 illustrate the residual factor plots for data sets 10 (1450c) and 7 (1450b, Boulder, Lot 1980) fit to Model 3. Note that, for 1450c (Fig. 12), the residuals show no structure. However, for 1450b (Fig. 13) the structure in the residuals for bilinear Model 3 is inadequate for the data set as evidenced by the clear peaking or oscillatory pattern in the frames plotting residuals versus *T* and predicted λ.

#### 6.1.4 Residual Plots for Assessment of Statistical Model Adequacy

The fundamental assumptions of any least squares fit regression model are that the residuals behave like random drawings from a fixed distribution having fixed location and fixed variation. That is, the residuals are independent, identically distributed, and conform to a normal distribution. The 4-plot [49] is a graphical tool designed to assess these assumptions. It consists of:

1. A run sequence plot [50] of the ordered (either directionally, e.g., along a fitted line or profile in a fitted surface, or temporally, i.e., in the order in which the data were taken) residuals. This plot can be used to assess the assumption of fixed location and variation. That is, one can use it to ascertain whether there appears to be a trend or whether the residual variance appears to be increasing or decreasing.
2. A lag plot [51] of the residuals. The lag plot is used to assess a weaker, testable surrogate for independence, specifically, first-order autocorrelation.
3. A histogram [52] of the residuals. The histogram can help assess the shape and characteristics of the underlying distribution such as symmetry, skewness, multimodality.
4. A normal probability plot [53] of the residuals. This plot is used specifically to assess whether the residuals follow an approximately normal distribution.

**Fig. 14:**
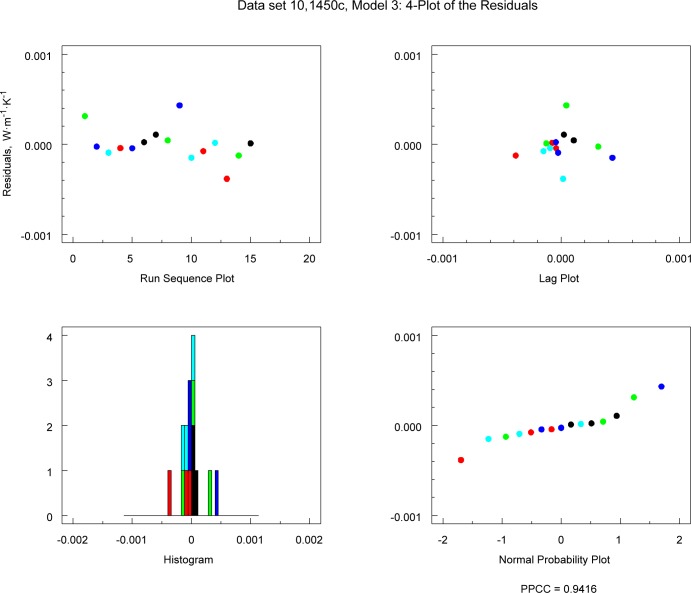
4-plot of the model residuals for data set 10 (1450c).

**Fig. 15:**
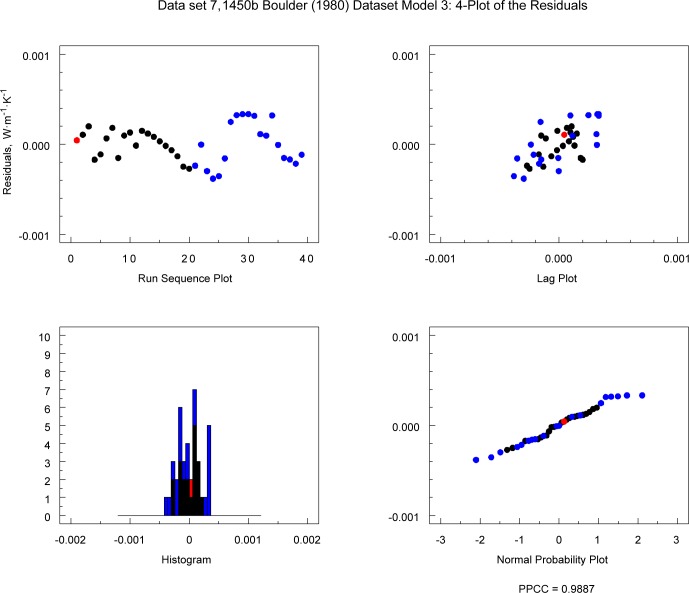
4-plot of the model residuals for data set 7 (1450b, Boulder Lot 1980).

If the 4-plot shows that the underlying assumptions are not satisfied, this finding may indicate that the model can be improved. Figures 14 and 15 show the 4-plots for the Model 3 for the 1450c and 1450b (Boulder, Lot 1980), respectively. The plot for 1450c (Fig. 14) does not indicate any serious problems with the underlying model assumptions. The run sequence plot for 1450b (Boulder, Lot 1980), however, indicates that Model 3 is inadequate because there is significant structure in the residuals (Fig. 15).

#### 6.1.5 Model Predicted Response versus Empirical Response Plots

**Fig. 16:**
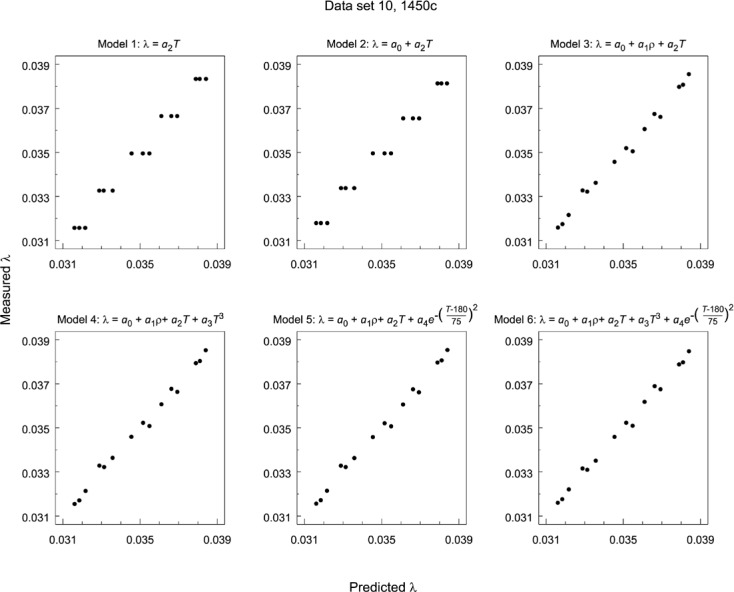
Model predicted response versus empirical response plots for data set 10 (1450c).

Irrespective of whether a model is linear, nonlinear, univariate, multivariate, goodness-of-fit can always be assessed by simply plotting model predictions of response (of λ) versus the corresponding empirical response (λ) values. The better the predictive power of the model, the more closely the prediction versus empirical profile should resemble a straight line with a 45 ° slope. In a series of such plots, Fig. 16 shows for data set 10, successively more complex models in temperature predictions plotted against the raw response (λ) values. None of the models of increasing complexity show any significant improvement over the simplest model linear in *T*.

### 6.2. Analytic Techniques

The primary analytic techniques used for model (goodness-of-fit) assessment and comparison include residual standard deviation (RESSD), *t*-statistics for coefficients, and Bayesian Information Criteria (BIC).

#### 6.2.1 Residual Standard Deviation

As already discussed, the residuals are the empirical response data points minus the modeled response data points and thus represent “what is left over” in the raw data after the model has been fit. (Note that the term residual has been referred to as “deviation” in earlier SRM analyses [16–18]). The residual standard deviation (RESSD), defined as the square root of the sum of squares of the residuals divided by the sample size minus the number of parameters being estimated, is the principal measure of how much variability in the data remains unexplained after the model has been fit. Least squares fitting is defined by minimization of the RESSD over the parameter estimates. The units of RESSD are the same as the data.

**Fig. 17:**
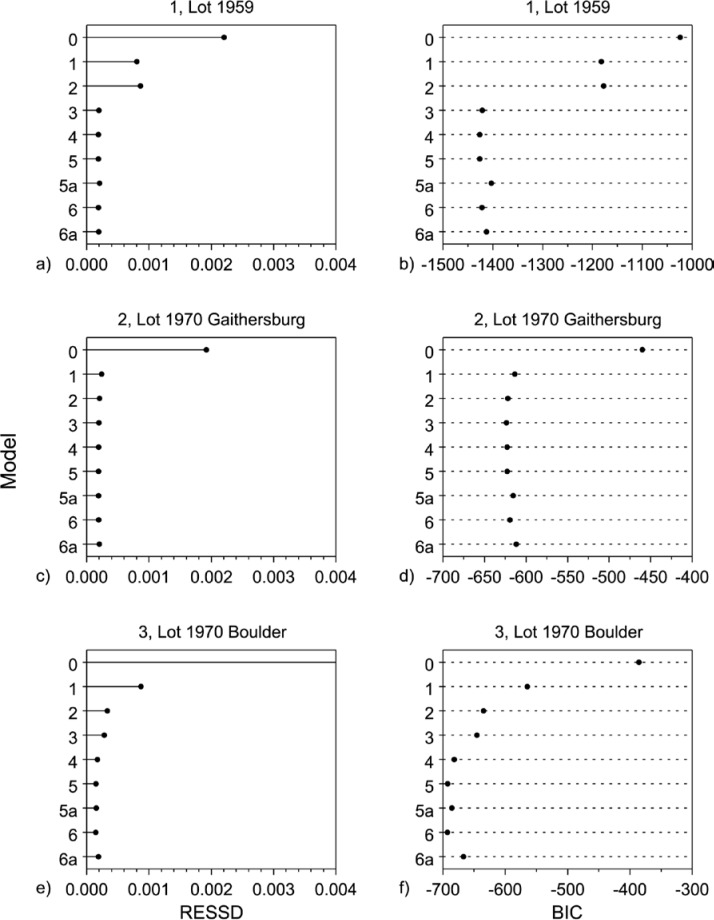

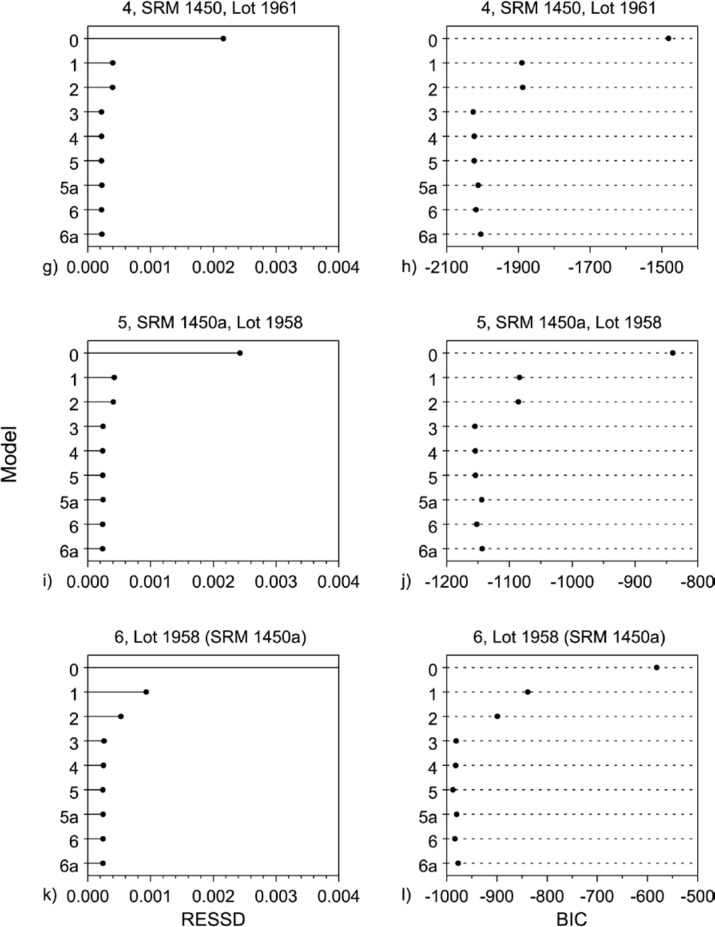

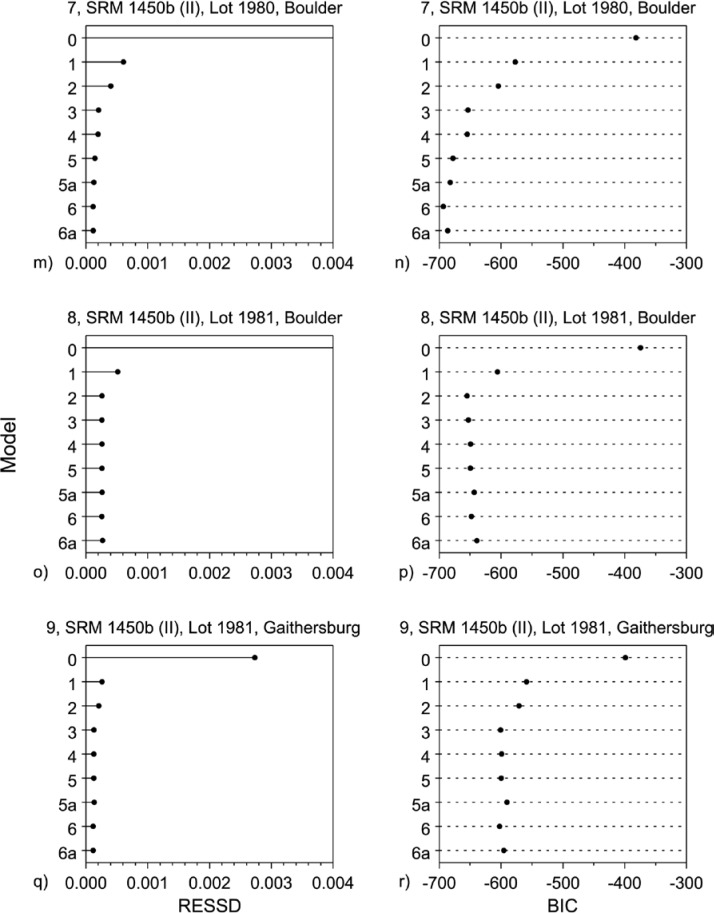

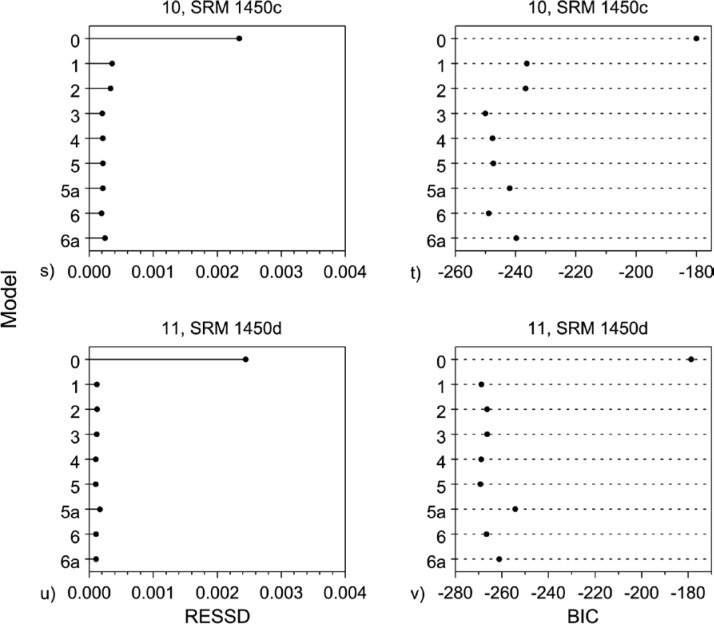
a–f. Graphical summary of RESSD and BIC values for data sets 1, 2, and 3. g–l. Graphical summary of RESSD and BIC values for data sets 4, 5, and 6. m–r. Graphical summary of RESSD and BIC values for data sets 7, 8, and 9. s–v. Graphical summary of RESSD and BIC values for data sets 10 and 11.

In comparing models’ goodness-of-fit, smaller RESSD is better. However, it is possible to “over fit” by introducing un-needed, physically irrelevant parameters. Such over-parameterization may lead to a smaller RESSD, but can actually bias the model through the introduction of physically irrelevant parameters. Over-fitted models tend to be unstable in the sense that small changes in the data can result in large changes in the parameter estimates. Part of the art of model selection is adjudicating the tradeoff between minimizing variance (RESSD squared) and introducing undesirable bias by the introduction of too many variables or parameters. Summary RESSD values for each model for each of the data sets are displayed graphically in Fig. 17a through 17v and in tabular form in Sec. 7.

#### 6.2.2 *t*-Statistics for Fitted Coefficients

The *t*-statistics for least squares fitted model coefficient(s) are designed to test the necessity or significance of the terms of the model represented by the coefficient(s). Each value of the *t*-statistic enables a formal test of the hypothesis that the fitted coefficient is zero or non-zero (i.e., “statistically indistinguishable from “zero”) with some pre-specified degree of confidence (e.g., 95 %). A widely used rule of thumb (for sample sizes greater than 7 or 8) compares the absolute value of the *t*-statistic for a given coefficient with the value of 2.

- |*t*| ≥ 2 suggests that the coefficient is statistically distinguishable from zero with 95 % confidence, and hence should be included in the model.
- |*t*| < 2 signifies that the coefficient is not statistically distinguishable from zero with 95 % confidence, and hence should not be included in the model.

Clearly, coefficients and the variables to which they attach should not be included in the model if their contributions to predicting response are essentially indistinguishable from zero. So, for example, for the fit of the bilinear (ρ, *T*) model illustrated in Table 8, the low value of the *t*-statistic associated with *a*_0_ indicates that it should be deleted from the model. Note may be taken of the fact that whereas the coefficient for temperature is unambiguously non-zero (*t* = 58.1), the call with regard to the inclusion of density in the model is more marginal (*t* = 2.3).

#### 6.2.3 Bayesian Information Criteria

The Bayesian Information Criteria (BIC) is one of a number of “information” criteria designed to provide objective assessment of the tradeoff between the number of parameters incorporated in a model and the goodness-of-fit of the model. Adding parameters to a model will often reduce the RESSD, nominally improving the fit. While inspection of the values of *t* can lead to the non-inclusion of certain parameters in a model, *t*-statistic inspection cannot always be counted upon to reject spurious (nonphysical) variables. Information criteria attempt to assess the penalty incurred in model bias terms for enhanced RESSD (goodness-of-fit) resulting from the inclusion of possibly irrelevant variables or terms in the model. A simple form of the BIC statistic for regression model comparison is a function of the sample size (*n*), the number of parameters (*p*) included in the model, and the residual variance for the *p*-parameter model, *RESSD*^2^, with a denominator *n* (instead of *n* − *p*) as given in Eq. (18) [54–55].

$$\mathrm{BIC}=n\times \ln\left(RESSD^{2}\right)+p\times \ln(n)$$ (18)

If two models are compared, all other considerations (RESSD, *t*, diagnostic graphics) being equal, the model with the minimum BIC value would be selected as the most appropriate model. It is clear from Eq. (18) that increasing the RESSD and/or the increasing number of explanatory parameters (*p*) will increase the value of the BIC statistic. So, in particular, lower BIC values arise from enhanced fit in model residual terms (RESSD), or fewer parameters (*p*), or both. Information-type criteria have advantages over analysis of variance (ANOVA) model comparison approaches in that the models being evaluated need not be linear, and the models being compared need not be nested. Summary BIC values for each of the models for each of the data sets are provided in graphical form in Figs. 17a–17v and tabular form in Sec. 7.

### 6.3 Parsimony

Another extremely important model discrimination tool is the simplest one of all: parsimony. The principle of parsimony instructs us, confronted with a choice of competitive models, to select the model that is simplest. In the case of nested multilinear models that we are dealing with here, that means – again, all other choice factors being equal – the model with the fewest terms and simplest parameterization.

## 7. Model Selection

Summary results in terms of the residual standard deviation (RESSD) and Bayesian information criteria (BIC) of the retrospective analysis for data sets 1–11 across all models are represented graphically in the sequence of multi-plots in Figs. 17a–17v. For each frame, the model number (0, 1, 2, 3, 4, 5, 5a, 6, 6a) is plotted on the *y*-axis and the RESSD or BIC is plotted on the *x*-axis in column 1 or column 2, respectively. The Model $0(\bar{\lambda })$ provides a baseline value for the RESSD and the BIC. The minimum value for the RESSD or BIC is indicative of the optimum model for a particular data set. The dominance of the temperature term is shown by the large drop in RESSD or BIC when this term is added. The effect of the density term on the RESSD or BIC is by contrast much smaller.

The values given in Figs. 17a–17v are tabulated in Table 9. Models 5 and 6 apply primarily to low-temperature data; Models 5a and 6a, which offered no improvement, are omitted. The first portion of Table 9 provides the residual standard deviation (RESSD), in milliwatts per meter per kelvin, for data sets 1–11 across all models. Because the RESSD is computed with the same units as the data, the values can be compared not only within but also across data sets. For each data set, the optimum model was selected on the basis of the graphical and analytical criteria discussed in Section 6 with special emphasis on parsimony (in particular for *T* versus *T*^3^ models). Optimum model values for RESSD are identified in boldface and range from 0.122 mW·m^−1^·K^−1^ (data set 11) to 0.261 mW·m^−1^·K^−1^ (data sets 6 and 8). Relative values of RESSD, based on a nominal value of λ of 32 mW·m^−1^·K^−1^ near 300 K, are approximately 0.4 % to 0.8 %.

The second portion of Table 9 provides the Bayesian information criteria (BIC) for each data set across all models. Comparison of BIC values is valid within a data set, not valid across data sets. The BIC value for the optimum model for each data set, is identified in boldface.

**Fig. 18:**
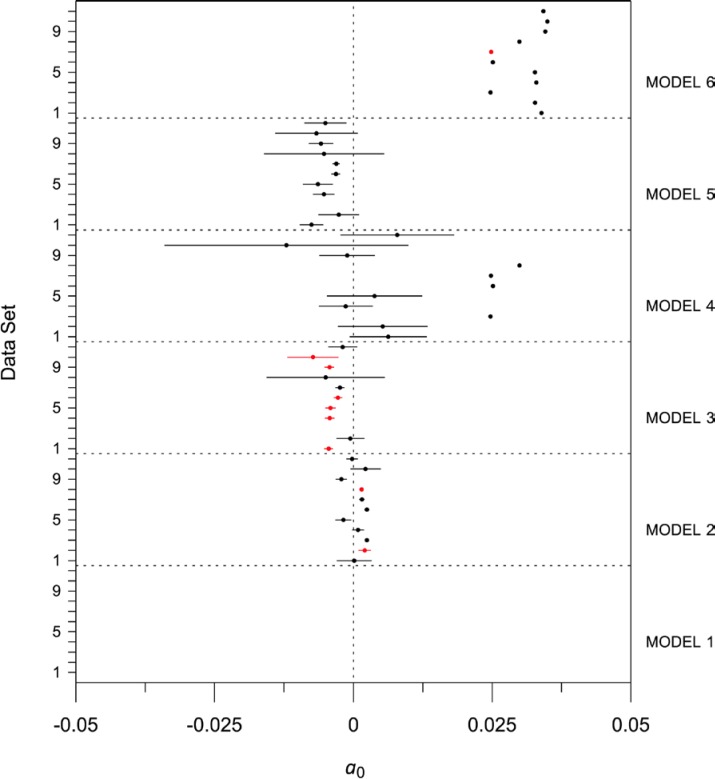

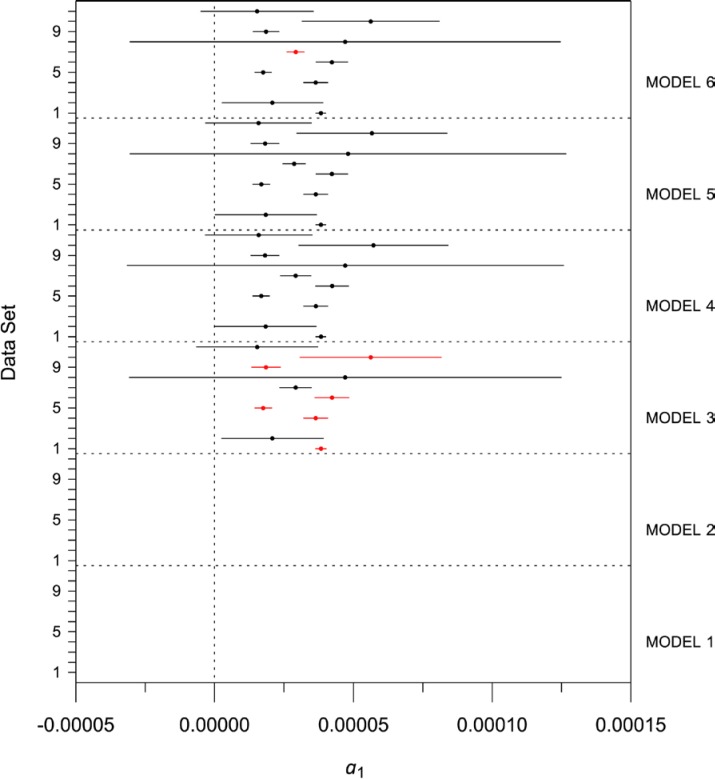

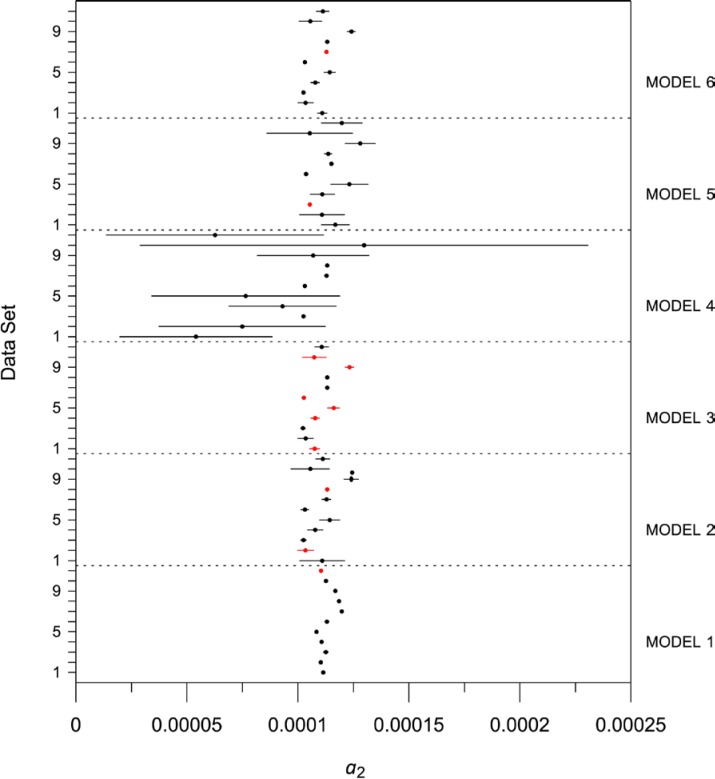

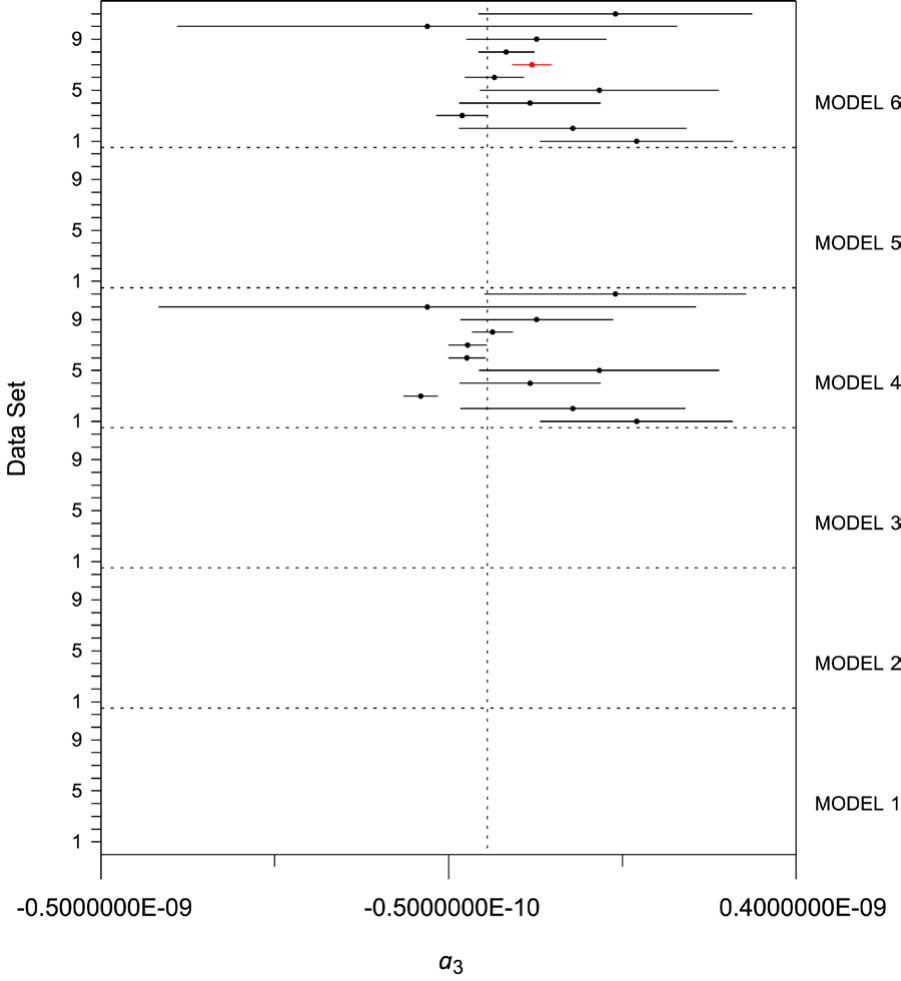

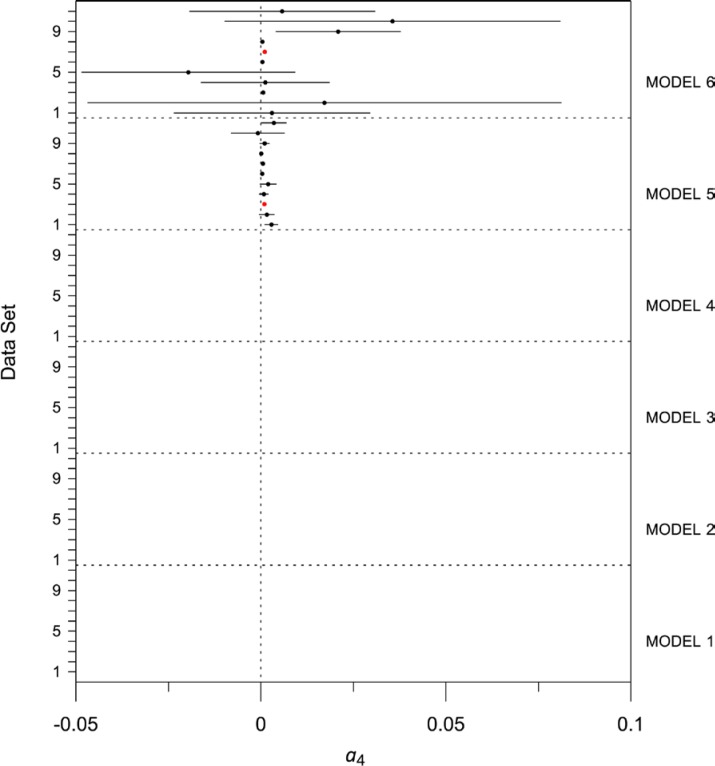
**a**. Graphical summary of regression coefficient *a*_0_ (intercept) shown as individual data points with associated values of the *t*-statistic at 95 % confidence intervals shown as horizontal line (restricted range). Color code (red) represents selected model for a particular dataset. **b**. Graphical summary of regression coefficient *a*_1_ (bulk density [ρ] parameter) shown as individual data points with associated values of the *t*-statistic at 95 % confidence intervals shown as horizontal line (restricted range). Color code (red) represents selected model for a particular dataset. **c**. Graphical summary of regression coefficient *a*_2_ (temperature [*T*] parameter) shown as individual data points with associated values of the *t*-statistic at 95 % confidence intervals shown as horizontal line (restricted range). Color code (red) represents selected model for a particular dataset. **d**. Graphical summary of regression coefficient *a*_3_ (cubic temperature [*T*^3^] parameter) shown as individual data points with associated values of the *t*-statistic at 95 % confidence intervals shown as horizontal line (restricted range). Color code (red) represents selected model for a particular dataset. **e**. Graphical summary of regression coefficient *a*_4_ (exponential temperature [*e ^f^*^(^*^T^*^)^] parameter) shown as individual data points with associated values of the *t*-statistic at 95 % confidence intervals shown as horizontal line (restricted range). Color code (red) represents selected model for a particular dataset.

For all the models selected in Table 9, all of the relevant *t*-statistics confirm the hypothesis that the coefficients are statistically different than zero at 95 % confidence. The values of the *t*-statistic are displayed, along with all fits of all models to all data sets, in Fig. 18. Figure 18 graphs *t*-statistics based 95 % confidence intervals, on a dataset within model basis, across all models, for one specified parameter (*a_i_*) at a time. Horizontal confidence lines crossing zero are indicative of the specified regression coefficient being statistically indistinguishable from zero for the model/dataset combination chosen. For the *a*_2_ (temperature coefficient) parameter, for example, all fitted coefficients are significant, and it is noticeable that the introduction of the *T*^3^ term in Model 4 considerably broadens the uncertainty associated with the coefficient of *T*, probably a result of multicollinearity of *T* and *T*^3^. Additional figures for all of the modeling parameters are given online^3^.

The optimal model choices for data sets 1–11 are summarized in Table 10. The dominant generic model for 6 of the 11 data sets is the bilinear Model 3. For data set 11, the additive constant, *a*_0_, is not required. Data set 7 supports the inclusion of a cubic temperature term. Data sets 3 and 7 incorporate an exponential temperature term.

Table 11 summarizes the regression coefficients for data sets 1–11. As noted in Fig. 18, the coefficients for *a*_2_ are extremely consistent across models, ranging from 1.1×10^−4^ W·m^−1^·K^−2^ to 1.2×10^−4^ W·m^−1^·K^−2^. The physical meanings for the coefficients are discussed in Sec. 7.1–7.5.

### 7.1 Heat Transfer in Fibrous Insulations

The contributions of the different heat transfer mechanisms for fibrous insulating materials have been investigated by Bankvall [56] and Pelanne [57–58]. The total heat transfer in a porous material, such as fibrous-glass board, can be considered a combination of the following individual mechanisms:

- gas conduction for the interstitial nitrogen and oxygen molecules restrained in the insulation that increases linearly with *T*;
- radiation that decreases with increasing ρ and increases as a function of *T*^3^, and,
- solid conduction along the meandering, discontinuous network of fibrous glass paths that increases (linearly) as a function of ρ.

**Fig. 19:**
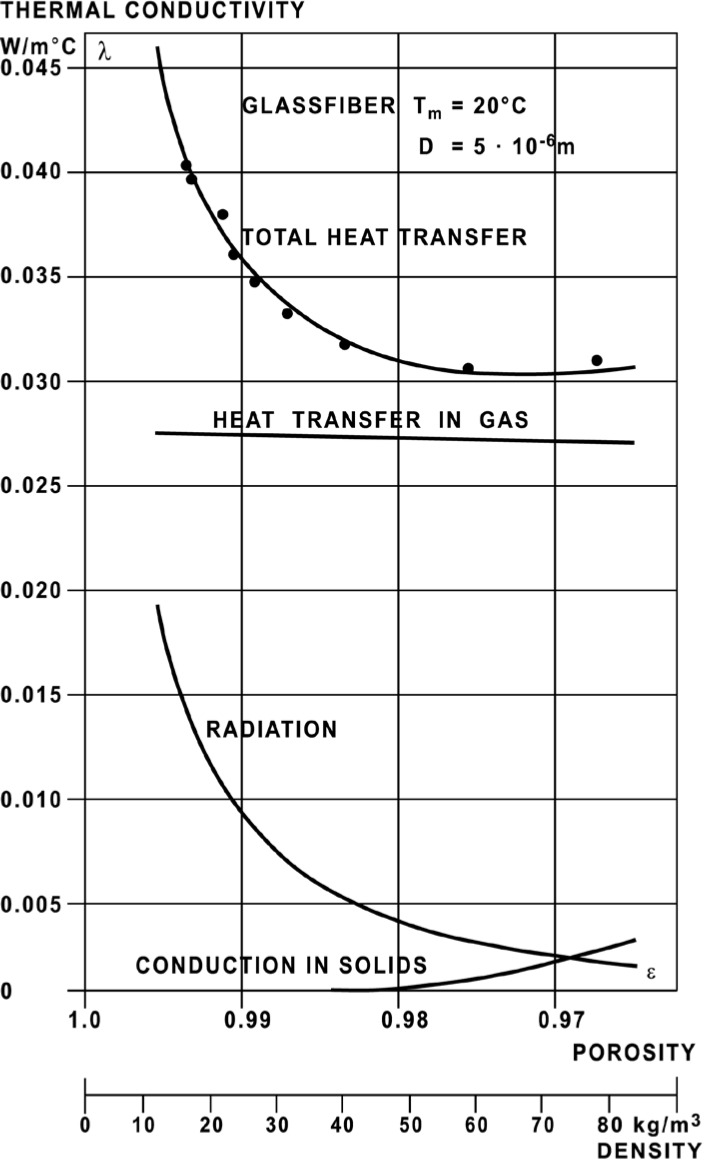
Mechanisms of heat transfer in glass fiber insulation, • measured;—calculated values [56]. (Reprinted, with permission, from ASTM STP 544 - Heat Transmission Measurements in Thermal Insulations, copyright ASTM International, 100 Barr Harbor Drive, West Conshohocken, PA 19428.)

Although additional mechanisms such as natural convection can also be present, Bankvall [56] found no indications of natural convection in a low-density glass fiber insulation and air. The contributions of the above mechanisms to the (total) effective thermal conductivity (in W·m^−1^·° C^−1^) for a fibrous-glass insulation at *T_m_* of 20 °C are illustrated in Fig. 19 (reproduced from Ref. [56]). The dual *x*-axis plots porosity (dimensionless) and bulk density (kg·m^−3^). Figure 19 clearly shows that the contribution due to gas conduction is dominant and the radiation contribution is significant at low densities, and decreases with increasing bulk density. The contribution due to solid conduction is significant at bulk densities greater than 70 kg·m^−3^.

### 7.2 Validity of the *T* Term

The dominant analytic feature, present in all data sets, is the clear, strong linearity of λ in terms of *T*. Recall from Table 4 that values for the *T* regression coefficient *a*_2_, across lots 1450b, 1450c, and 1450d have similar slopes of approximately 0.0001 W·m^−1^·K^−1^ per K, reflecting the strongly linear relationship between λ and *T* for this class of materials, and *T*, ρ ranges (Fig. 4). Using the thermodynamic properties and state equations in the NIST Reference Fluid Thermodynamic and Transport Properties Database (REFPROP) [59], thermal conductivity values for air were calculated at atmospheric pressure (1.01 MPa). At 250 K and 350 K, the thermal conductivities of air computed by REFPROP are 0.022654 W·m^−1^·K^−1^ and 0.029846 W·m^−1^·K^−1^, respectively. These values give a slope of 7.19×10^−5^ W·m^−1^·K^−1^ per K, or about 72 % of the total contribution. The balance (28 %) is due to the solid conduction contribution and some radiation contribution.

### 7.3 Validity of the ρ Term

The general relationship between apparent thermal conductivity and bulk density exhibited in Fig. 19 is useful in explaining the validity of the ρ term in the analysis of the SRM data where it occurs. At low densities, the apparent thermal conductivity exhibits a high degree of curvature due to the significant mechanism of radiative heat transfer. At high densities, the radiative contribution decreases and the resulting curve is linear due primarily to conductive heat transfer (Fig. 19). The transition region, which forms a relative minimum from 60 kg·m^−3^ to 80 kg·m^−3^ (Fig. 19), is moderately flat.

As observed in Fig. 3, thermal conductivity is a weak linear function of bulk density, signifying that thermal conductivity data for the 1450 lots are representative of the conductive (right) side of the general (λ-ρ) curve shown in Fig. 19. Careful inspection of Fig. 3 reveals that, for a particular mean temperature, the slopes of the thermal conductivity data increase at high bulk densities and decrease at low bulk densities. Recall that the same effect was observed previously for the bulk density regression coefficients, *a*_1_, in Table 4.

There are three data sets (2, 8, and 11) in Table 11 that do not include a bulk density term (i.e., *a*_1_ = 0). A valid question is when does the regression coefficient (*a*_1_) for bulk density occur and under what conditions? To answer this question, Table 12 re-sorts the information by model number and includes minimum and maximum ρ values (from Fig. 8), density ranges (Δρ), regression coefficients (*a*_1_) sub-sorted within the model number, and values of *t*, where appropriate. Estimates for *a*_1_ and values of *t* for data sets 2, 8, and 11 are included in Table 12 for comparison purposes

Values of *a*_1_ for the bilinear model in ρ and *T* are indicative of the bulk density inclusion region for a particular data set. Low values for *a*_1_ are reflective of material lots having low values of bulk density and, conversely, high values of *a*_1_ are indicative of material lots having high values of bulk density. In some data sets, large ranges encompassing low and high values of bulk density tend to average out in the resulting value for *a*_1_.

The data in Table 12 suggest that, for low regions of bulk density (ρ ≤ 140 kg·m^−3^) coupled with a restrictive density range (Δρ ≤ 13 kg·m^−3^), *a*_1_ is not statistically significant. Under these conditions, the bulk density from a material lot is representative of a very short section of the “flat” part of the λ-ρ curve (Fig. 19). Consequently, it is not surprising that the bulk density regression term is not significant in the resulting model. The results of Table 12 would suggest that developers of future material lots might consider employing bulk densities in the region less than 140 kg·m^−3^ coupled with a restrictive range (on the order of Δρ ≤ 13 kg·m^−3^).

### 7.4 Validity of the *T*^3^ Term

Radiation transmission, when expressed as a thermal conductivity, includes the following temperature difference ratio.

$$\frac{T_{h}^{4}-T_{c}^{4}}{T_{h}-T_{c}}$$ (19)

An explanation for the validity of the *T*^3^ approximation for the relationship given in Eq. (19) can be derived (from unpublished notes by B. A. Peavy) as follows.

$$\frac{T_{h}^{4}-T_{c}^{4}}{T_{h}-T_{c}}=(T_{h}+T_{c})\times (T_{h}^{2}+T_{c}^{2})$$ (20)

Letting *T_h_* = 2*T_m_* − *T_c_* (Eq. (5)) and substituting in Eq. (20) yields

$$\begin{array}{l} \frac{T_{h}^{4}-T_{c}^{4}}{T_{h}-T_{c}}=2T_{m}\times \left({\left(2T_{m}-T_{c}\right)}^{2}+T_{c}^{2}\right)\\ \;=4T_{m}\times (2T_{m}^{2}-2T_{m}T_{c}+T_{c}^{2})\\ \;=4T_{m}^{3}\times \left(2-2\frac{T_{c}}{T_{m}}+\frac{T_{c}^{2}}{T_{m}^{2}}\right)\\ \;=4T_{m}^{3}\times \left(1+\left(1-2\frac{T_{c}}{T_{m}}+\frac{T_{c}^{2}}{T_{m}^{2}}\right)\right)\\ \;=4T_{m}^{3}\times \left(1+{\left(1-\frac{T_{c}}{T_{m}}\right)}^{2}\right) \end{array}$$

Final substitution for *T_m_* in the denominator and simplifying yields Eq. (21).

$$\frac{T_{h}^{4}-T_{c}^{4}}{T_{h}-T_{c}}=4T_{m}^{3}\times \left(1+{\left(\frac{T_{h}-T_{c}}{T_{h}+T_{c}}\right)}^{2}\right)$$ (21)

Let

$$\alpha ={\left(\frac{T_{h}-T_{c}}{T_{h}+T_{c}}\right)}^{2}$$ (22)

The goodness of the *T*^3^ approximation therefore depends on the magnitude of the ratio α. For typical temperature differences (20 K to 25 K) and temperature ranges (100 K to 340 K) of interest, the following values of α are computed. As can be seen, the values of α are quite small (less than 0.01).

- For *T_h_* = 120 K and *T_c_* = 100 K, α = 0.00826
- For *T_h_* = 320 K and *T_c_* = 300 K, α = 0.00104

Thus,

$$\frac{T_{h}^{4}-T_{c}^{4}}{T_{h}-T_{c}}\approx 4T_{m}^{3}$$ (23)

### 7.5 Validity of the Exponential Temperature Term

The effect of the multiplicative product *a*_4_ (Table 4) and the exponential function for *T* is illustrated in Fig. 6. The product adds about 1.2 mW·m^−1^·K^−1^ to the fitted function given in Eq. (1) at 180 K and diminishes considerably at the temperature extremes of 100 K and 330 K. The effect is small (less than 0.5 mW·m^−1^·K^−1^) at 255 K to negligible (less than 0.1 mW·m^−1^·K^−1^) at 300 K and above. The scientific reason for the necessity of the exponential term for certain low-temperature data sets is not understood. The inclusion seems to have been motivated empirically.

Models 5 and 6 include an exponential temperature term originally used in the published certification of 1450b (Table 4), primarily for inclusion of the low-temperature data. Models 5a and 6a are modifications where the additive and multiplicative parameters, *b* and *c*, are allowed to float and self-select for optimum values in the least squares fitting process. Interestingly, these more general forms of the model did not prevail in the cases (Figs. 17f, 17n, and 17p) where the addition of an exponential term in *T* was considered beneficial to the overall fit, suggesting that the constants selected for the SRMs were optimal.

## 8. Discussion

The main results of the fit analyses can be summarized as follows:

1. The dominant analytic feature, present in all data sets, is the clear, strong linearity of thermal conductivity in terms of temperature.
2. The second prominent feature is the subsidiary linearity in terms of material bulk density (ρ). Conditions for inclusion in the model are discussed in Sec. 7.3.
3. The dominant generic model for six of the eleven data sets is, therefore, the bilinear Model 3:
  $$\lambda (\rho ,T)=a_{0}+a_{1}\rho +a_{2}T$$ (24)
  For one data set, the additive constant, *a*_0_, is not required.
4. Previous researchers at NIST have suggested the incorporation of a cubic term in *T* in the model [7]. The scientific rational for this effect is discussed in Sec. 7.4. One data set analyzed here does support the inclusion of a cubic temperature term.
5. Other researchers have suggested the incorporation of an exponential term in *T*, centered on 180 K, into the model. While there appears to be no scientific rationale for inclusion of this term, empirically it is found to improve predictions for two low temperature data sets (3, 7) studied here. However, low temperature data set 8 did not require this term. In fact, this data set was found to be a linear function of *T* (Table 12).
6. For the certification of SRM 1450b(II), data sets 7–9 were combined by consensus (established as an acceptable mode later in Ref. [35]). That is, the data from two NBS laboratories and 3 different apparatus were aggregated and Model 6 was successfully applied to the aggregated data. The consensus process could be considered unusual, however, because a more detailed assessment shows that regression fits for the individual data sets were different.
7. With respect to uncertainties, standard statistical practices can serve to generate (simultaneous) confidence, tolerance, or prediction limits about any form of the model from among the set of models examined here. However, NIST currently maintains, in ongoing electronic format, a set of computational algorithms based on the GUM for the careful determination of uncertainties in parallel with NIST thermal conductivity measurements [18]. The current practice is to cite the more conservative uncertainties derived from calculations based on the GUM. The reduction in expanded uncertainty over time (Table 5) is attributed primarily to changes in two factors: improvement in the production procedure due to the introduction of a formal statistical design in the planning of the measurements and modernization of the measurement facilities.

## 9. Summary and Recommendations

Data sets representing Standard Reference Material (SRM) 1450, Fibrous Glass Board, subsequent renewals 1450a, 1450b, 1450c, and 1450d, as well as undeveloped 1450 SRMs have been re-analyzed in this investigation. The data examined in this study cover 56 years of activity by the National Institute of Standards and Technology (NIST) in providing calibration services and subsequently developing and providing thermal insulation SRMs, specifically molded fibrous-glass board nominally 25 mm thick to the public. As a group, the eleven data sets cover two thicknesses (13 mm and 25 mm), a range of bulk densities from 60 kg·m^−3^ to 180 kg·m^−3^, and mean temperatures from 100 K to 340 K.

The major findings are that the dominant analytic feature, present in all data sets, is the clear, strong linearity of thermal conductivity (λ) in terms of (mean) temperature (*T*), and a more modest linearity in terms of material bulk density (ρ). The prevailing generic model for six of the eleven data sets is therefore the bilinear model in ρ and *T*:

$$\lambda (\rho ,T)=a_{0}+a_{1}\rho +a_{2}T$$

In specific cases, one data set supported the inclusion of a cubic temperature term probably as a result of radiative heat transfer processes. It was found that for two data sets with low-temperature data support the inclusion of an exponential term in *T* improved the model predictions.

The final models for three of the eleven data sets having moderate temperature ranges did not include a term for bulk density (i.e., *a*_1_ was equal to zero). The results of this retrospective analysis revealed that the term *a*_1_ is not necessary for regions of bulk density less than 140 kg·m^−3^ coupled with a restricted range less than 13 kg·m^−3^ for the material lot. Physically, the bulk density region less than 140 kg·m^−3^ corresponds to a fairly flat portion of the curve representing the relationship for bulk density and thermal conductivity near ambient conditions. It is therefore recommended that future renewals of 1450 consider only material lots having bulk densities less than 140 kg·m^−3^ and, preferably, near a nominal value of 128 kg·m^−3^ in order to meet customer applications. Ideally, the upper limit for the bulk density range for the material lot should be no more than 10 kg·m^−3^, or less.

This investigation also strongly reinforced the benefits of using a detailed certification test plan focused on a careful design approach for measurement and subsequent analyses. An acceptable statistically designed experiment yields optimal unambiguous information obtained from a minimum number of tests. The two of the most recent renewals, 1450c and 1450d, required only 15 independent tests by using designs that specified three levels for bulk density and five temperature settings. The density levels were, however, appropriately determined by 100 % sampling of the material lot.

## Figures and Tables

**Table 1:** Chronology of SRMs 1450–1450d, Fibrous Glass Board

Designation	Year acquired	Year issued	Reference	Laboratory facility
1450	1961	1978^a^	[7]	b
1450a	1958	1979^a^	[7]	b
1450b(I)	1981	1982	—	b
1450b(II)	1980, 1981	1985	[16]	b, c, d
1450c(I)	1996	1997	[17]	e
1450c(II)	1996	2010	—	e
1450d	2009	2011	[18]	e

a Issued initially under the preceding NBS calibration program.

b NBS 200 mm square guarded-hot-plate apparatus, Gaithersburg, Maryland.

c NBS 300 mm diameter guarded-hot-plate apparatus, Gaithersburg, Maryland.

d NBS 200 mm diameter guarded-hot-plate apparatus, Boulder, Colorado.

e NIST 1016 mm diameter guarded-hot-plate apparatus, Gaithersburg, Maryland.

**Table 2:** Supplementary properties for SRMs 1450–1450d, Fibrous Glass Board

Measured property		SRM designation	Reference
1) binder content by mass		1450b	[16], p. 16
2) compressive strength		1450c	[17], pp. 13, 16
3) effect of compression on thermal conductivity		1450 1450a	[7], pp. 350–351
4) effect of drying temperature on thermal resistance		1450 1450a	[7], p. 350
5) effect of gas pressure on thermal conductivity		1450a 1450c	[25] [26]
6) microstructure^a^		1450 1450c	[7], pp. 345–346 [17], p. 13
7) moisture sorption isotherm by fixed-point humidities		1450c	[17], p. 16
8) long-term replicate measurements:	11 y 16 y	1450 1450a	[7], p. 357
9) specific heat (differential scanning calorimeter)		1450b	[27]
10) thermogravimetry analysis (binder content)		1450b 1450c	[27] [17], pp. 14–15
11) within-board bulk density		1450c	[17], p. 8

a Photomicrographs of fiber shape, size, and arrangement by scanning electron microscopy.

**Table 3:** Thermal characterization parameters for SRMs 1450–1450d

Designation	Number of measurements	Specimen pairs	ρ (kg·m^−3^)	*T* (K)	Model function form
1450	121	71	110 to 170	255 to 330	*a*_0_, ρ, *T*^3^
1450a	70	33	60 to 140	255 to 330	*a*_0_, ρ, *T*^3^
1450b(I)	51	28	110 to 150	260 to 330	*a*_0_, ρ, *T*, *T*^3^
1450b(II)	114	33	110 to 150	100 to 330	*a*_0_, ρ, *T*, *T*^3^, *e^f^*^(^*^T^*^)^
1450c(I)	15	15	150 to 165	280 to 340	*a*_0_, ρ, *T*
1450c(II)	—	—	150 to 165	280 to 340	*a*_0_, ρ, *T*
1450d	15	15	114 to 124	280 to 340	*T*

**Table 4:** Regression coefficients from SRMs 1450–1450d Certificates

Designation	*a*_0_ (W·m^−1^·K^−1^)	*a*_1_ (W·m^−2^·K^−1^·kg^−1^)	*a*_2_ (W·m^−1^·K^−2^)	*a*_3_ (W·m^−1^·K^−4^)	*a*_4_ (W·m^−1^·K^−1^)
1450	1.7062×10^−2^	3.648×10^−5^	0	4.037×10^−10^	0
1450a	1.930×10^−2^	1.534×10^−5^	0	4.256×10^−10^	0
1450b(I)	1.645×10^−3^	2.2122×10^−5^	9.2087×10^−5^	1.0753×10^−10^	0
1450b(II)	−2.228×10^−3^	2.743×10^−5^	1.063×10^−4^	6.473×10^−11^	1.157×10^−3^
1450c(I)	−7.7663×10^−3^	5.6153×10^−5^	1.0859×10^−4^	0	0
1450c(II)	−7.2661×10^−3^	5.6252×10^−5^	1.0741×10^−4^	0	0
1450d	0	0	1.10489×10^−4^	0	0

**Table 5:** Specified uncertainties from SRMs 1450–1450d Certificates

Designation	Certified quantity	Certification format	Uncertainty (%)	Coverage factor
1450	*R*_0_	Table	2	*k* = 2^a^
1450a	*R*_0_	Table	2	*k* = 2^a^
1450b(I)	*R*_0_	Table	2	*k* = 3^a^
1450b(II)	*R*_0_	Table	2 to 3^b^	*k* = 3^a^
1450c(I)	*R*_0_	Table	1.6	*k* = 2
1450c(II)	*R*_0_	Table	1.6	*k* = 2
1450d	λ, ρ	Equation, λ(*T*)	1	*k* = 2

a Deduced by authors.

b 2 % for *T* from 250 K to 330 K; or, increasing to 3 % at 100 K.

**Table 6:** NBS/NIST guarded-hot-plate apparatus

Plate size (mm)	Geometry	Meter size (mm)	Heat flow direction	Laboratory site	Reference
203.2	Square	101.6	horizontal	Gaithersburg	[39]
304.8	Round	152.4	horizontal	Gaithersburg	[40]
203.2	Round	101.6	vertical	Boulder	[41]
1016	Round	406.4	vertical	Gaithersburg	[42]

**Table 7:** SRM 1450 and proto-1450 SRM data sets

Data set	Designation	Laboratory	Source	Appendix
1	Lot 1959	Gaithersburg	[7]^a^	A
2	Lot 1970	Gaithersburg	[7]^a^	B
3	Lot 1970	Boulder	[16]	C
4	1450, Lot 1961	Gaithersburg	[7]^a^	D
5	1450a, Lot 1958	Gaithersburg	[7]^a^	E
6	Lot 1958 (SRM 1450a)	Boulder	[16]	F
7	1450b(II), Lot 1980	Boulder	[16]	G
8	1450b(II), Lot 1981	Boulder	[16]	G
9	1450b(II), Lot 1981	Gaithersburg	[16]	G
10	1450c(II), Lot 1996	Gaithersburg	[17]	H
11	1450d, Lot 2009	Gaithersburg	[18]	I

a Original computer printouts in possession of the first author

**Table 8:** Fit results for data set 2, Model 3

Parameters		Estimate	Std. dev.	*t*
*a*_0_		−5.412×10^−4^	1.25×10^−3^	−0.43
*a*_1_	ρ	2.084×10^−5^	9.08×10^−6^	2.3
*a*_2_	*T*	1.035×10^−4^	1.78×10^−6^	58.1

**Table 9:** Retrospective analysis results

Data set	Model						
	0	1	2	3	4	5	6
	*a*_0_	*T*	*a*_0_,*T*	*a*_0_,ρ,*T*	*a*_0_,ρ,*T,T*^3^	*a*_0_,ρ,*T,e^f^*^(^*^T^*^)^	*a*_0_,ρ,*T,T*^3^, *e^f^*^(^*^T^*^)^

Residual standard deviation (mW·m^−1^·K^−1^)							
1	2.21	0.806	0.865	**0.197**	0.190	0.190	0.191
2	1.92	0.242	**0.209**	0.197	0.193	0.193	0.196
3	7.80	0.874	0.335	0.284	0.175	**0.153**	0.148
4	2.16	0.399	0.397	**0.221**	0.220	0.220	0.221
5	2.42	0.424	0.409	**0.244**	0.239	0.240	0.238
6	7.64	0.933	0.529	**0.261**	0.253	0.241	0.243
7	7.25	0.608	0.403	0.207	0.198	0.147	**0.117**
8	8.98	0.515	**0.261**	0.259	0.261	0.261	0.258
9	2.73	0.261	0.210	**0.131**	0.129	0.128	0.119
10	2.34	0.359	0.336	**0.205**	0.212	0.213	0.194
11	2.45	**0.122**	0.125	0.119	0.104	0.103	0.108

Bayesian information criteria (BIC)							

1	−1024	−1182	−1178	−**1421**	−1426	−1426	−1422
2	−460	−614	−**622**	−624	−623	−623	−619
3	−386	−565	−635	−645	−682	−**692**	−693
4	−1481	−1890	−1888	−**2026**	−2023	−2023	−2018
5	−840	−1084	−1086	−**1155**	−1154	−1154	−1152
6	−582	−838	−899	−**981**	−982	−987	−983
7	−382	−577	−604	−654	−654	−678	−**693**
8	−374	−606	−**655**	−653	−649	−649	−648
9	−399	−559	−571	−**601**	−599	−600	−602
10	−180	−236	−237	−**250**	−248	−247	−249
11	−179	−**269**	−266	−266	−269	−269	−267

**Table 10:** Optimum models for each data set

Data set	Designation	Laboratory	Model	Form
1	Lot 1959	Gaithersburg	3	*a*_0_, ρ, *T*
2	Lot 1970	Gaithersburg	2	*a*_0_, *T*
3	Lot 1970	Boulder	5	*a*_0_, ρ, *T*, *e ^f^*^(^*^T^*^)^
4	1450, Lot 1961	Gaithersburg	3	*a*_0_, ρ, *T*
5	1450a, Lot 1958	Gaithersburg	3	*a*_0_, ρ, *T*
6	Lot 1958 (SRM 1450a)	Boulder	3	*a*_0_, ρ, *T*
7	1450b(II), Lot 1980	Boulder	6	*a*_0_, ρ, *T*, *T*^3^, *e ^f^*^(^*^T^*^)^
8	1450b(II), Lot 1981	Boulder	2	*a*_0_, *T*
9	1450b(II), Lot 1981	Gaithersburg	3	*a*_0_, ρ, *T*
10	1450c(II), Lot 1996	Gaithersburg	3	*a*_0_, ρ, *T*
11	1450d, Lot 2009	Gaithersburg	1	*T*

**Table 11:** Regression coefficients for data sets 1–11

Data set	*a*_0_ (W·m^−1^·K^−1^)	*a*_1_ (W·m^−2^·K^−1^·kg^−1^)	*a*_2_ (W·m^−1^·K^−2^)	*a*_3_ (W·m^−1^·K^−4^)	*a*_4_ (W·m^−1^·K^−1^)
1	−4.455×10^−3^	3.828×10^−5^	1.075×10^−4^	0	0
2	2.046×10^−3^	0	1.035×10^−4^	0	0
3	−3.348×10^−1^	2.720×10^−3^	1.054×10^−4^	0	9.426×10^−4^
4	−4.265×10^−3^	3.645×10^−5^	1.078×10^−4^	0	0
5	−4.132×10^−3^	1.751×10^−5^	1.161×10^−4^	0	0
6	−2.771×10^−3^	4.232×10^−5^	1.028×10^−4^	0	0
7	2.482×10^−2^	2.922×10^−5^	1.129×10^−4^	5.800×10^−11^	1.083×10^−3^
8	1.481×10^−3^	0	1.132×10^−4^	0	0
9	−4.299×10^−3^	1.856×10^−5^	1.232×10^−4^	0	0
10	−7.266×10^−3^	5.625×10^−5^	1.074×10^−4^	0	0
11	0	0	1.105×10^−4^	0	0

**Table 12:** Summary of optimum models for each data set

Data set	Model	From	ρ range^a^ (kg·m^−3^)	Δρ (kg·m^−3^)	*a*_1_ (W·m^2^·K^−1^·kg^−1^)	*t*
11	1	*T*	114–124	10	(1.53×10^−5^)^b^	(1.5)
2	2	*a*_0_, *T*	117–130	13	(2.08×10^−5^)^b^	(2.3)^c^
8	2	*a*_0_, *T*	134–143	13	(4.71×10^−5^)^b^	(1.2)
5	3	*a*_0_, ρ, *T*	70–140	70	1.75×10^−5^	11.2
9	3	*a*_0_, ρ, *T*	112–142	30	1.86×10^−5^	7.2
4	3	*a*_0_, ρ, *T*	118–160	42	3.65×10^−5^	16.3
1	3	*a*_0_, ρ, *T*	100–180	80	3.83×10^−5^	38.2
6	3	*a*_0_, ρ, *T*	105–150	45	4.23×10^−5^	13.5
10	3	*a*_0_, ρ, *T*	150–165	15	5.63×10^−5^	4.8
3	5	*a*_0_, ρ, *T*, *e ^f^* ^(^*^T^*^)^	123.55–123.65	0.1	2.72×10^−3^	5.5
7	6	*a*_0_, ρ, *T*, *T* ^3^,*e ^f^* ^(^*^T^*^)^	120–145	25	2.92×10^−5^	18.1

a Density ranges determined from Fig. 8

b Value included for comparison, not included in selected model

c ρ dependency rejected in final model for other reasons

## Footnotes

1 In 1901, Congress established the National Bureau of Standards (NBS) to support industry, commerce, scientific institutions, and all branches of government. In 1988, as part of the Omnibus Trade and Competitiveness Act, the name was changed to the National Institute of Standards and Technology (NIST) to reflect a broader mission for the agency. For historical accuracy, this paper will use, where appropriate, NBS for events prior to 1988.

3 http://www.nist.gov/el/building_environment/heattrans/nist-srm-1450.cfm

4 In 1976, the U.S. Code of Federal Regulations mandated the establishment of the National Voluntary Laboratory Accreditation Program (NVLAP) to be administered by NBS [10]. The first laboratory accreditation program for thermal insulation was initiated in 1977 in response to a request from three thermal insulation trade associations [11]. The data and analyses for the insulation proficiency testing programs, rounds 1–3, were published in 1983 [12]; rounds 3–10 in 1985 [13]; and, rounds 11–30 in 2010 [14].

5 SRM 1450d Certificate is available at: https://www-s.nist.gov/srmors/view_detail.cfm?srm=1450d. SRM 1450c Certificate is available at: https://www-s.nist.gov/srmors/view_detail.cfm?srm=1450c. Other SRM Certificates are archived at https://www-s.nist.gov/srmors/certArchive.cfm.

## 10. Appendix A

**Table A1:** Data set 1, Lot 1959, Gaithersburg

λ_exp_	ρ	*T_m_*	*L_avg_*

(W·m^−1^·K^−1^)	(kg·m^−3^)	(K)	(mm)
0.03525	120.94	324.63	13.01
0.03168	120.46	293.67	13.08
0.03445	98.99	325.11	14.17
0.03084	98.99	293.71	14.20
0.03487	120.62	324.86	14.01
0.03129	120.62	293.73	14.02
0.02966	120.62	276.13	14.01
0.03574	142.56	325.04	14.32
0.03230	142.56	293.64	14.38
0.03557	142.24	325.24	14.77
0.03216	142.08	293.54	14.80
0.03253	141.28	296.32	14.02
0.03523	115.33	327.93	14.60
0.03169	114.85	296.39	14.67
0.03182	109.25	296.66	13.94
0.02943	108.77	272.63	13.99
0.03561	121.90	327.36	14.32
0.03210	121.58	295.88	14.36
0.03171	103.64	296.86	14.30
0.02913	103.16	272.67	14.37
0.03167	104.28	296.73	14.66
0.02909	103.80	272.58	14.73
0.03642	151.53	327.11	12.30
0.03293	151.37	295.76	12.44
0.03609	161.95	321.98	11.99
0.03685	170.28	325.12	12.13
0.03351	170.12	294.62	12.14
0.03706	165.47	327.00	11.86
0.03350	165.15	296.77	11.90
0.03100	165.15	272.62	11.89
0.03709	162.11	327.36	12.27
0.03354	161.47	296.87	12.32
0.03665	152.82	327.51	12.10
0.03311	152.82	297.15	12.10
0.03559	127.51	327.41	12.39
0.03216	127.35	297.10	12.42
0.03638	148.49	327.78	11.92
0.03292	148.49	296.93	11.91
0.03756	170.12	327.80	12.23
0.03402	170.12	296.99	12.24
0.03690	154.42	327.71	12.14
0.03335	154.42	297.07	12.14
0.03070	154.42	271.81	12.14
0.03419	170.28	297.02	11.94
0.03453	179.73	296.97	11.93
0.03760	170.92	327.86	12.25
0.03400	170.28	297.04	12.29
0.03141	170.60	271.59	12.26
0.03325	156.02	297.11	12.19
0.03616	140.80	328.10	12.31
0.03276	141.12	297.08	12.29
0.03028	141.12	271.94	12.29
0.03649	153.46	324.66	12.18
0.03368	162.91	296.91	11.87
0.03252	146.25	293.32	11.88
0.03598	146.25	324.79	11.89
0.03291	136.32	297.02	12.25
0.03598	136.00	324.93	12.26
0.03311	141.44	297.04	12.07
0.03257	138.56	297.02	11.93
0.03376	156.66	297.49	12.06
0.03720	163.39	327.60	11.86
0.03361	163.71	296.98	11.88
0.03352	163.87	297.24	11.97
0.03675	157.14	325.03	12.19
0.03344	157.30	294.14	12.17
0.03714	164.35	327.56	12.02
0.03371	164.03	297.09	12.03
0.03700	161.31	327.47	12.10
0.03363	161.31	297.05	12.09
0.03459	179.41	297.04	12.21
0.03465	181.65	296.77	11.96
0.03031	181.81	255.39	11.94
0.03684	156.98	327.38	11.89
0.03580	138.40	324.79	12.23
0.03220	137.60	293.32	12.28
0.03280	138.88	297.84	11.79
0.03635	158.74	321.82	11.90
0.03335	158.74	294.16	11.92
0.03402	167.23	296.94	12.26
0.03446	179.89	296.57	12.29
0.03762	180.85	326.11	12.22
0.03365	162.27	297.62	12.04
0.03355	155.22	297.21	12.27

## 11. Appendix B

**Table B1:** Data set 2, Lot 1970, Gaithersburg

λ_exp_	ρ	*T_m_*	*L_avg_*

(W·m^−1^·K^−1^)	(kg·m^−3^)	(K)	(mm)
0.03286	119.02	296.94	26.28
0.03305	127.67	296.74	26.00
0.03293	122.06	297.12	26.21
0.03303	126.39	296.79	26.21
0.03293	127.99	297.09	26.18
0.03608	127.99	327.38	26.19
0.02883	128.15	257.08	26.17
0.03265	121.58	297.27	25.89
0.03256	120.62	297.16	26.21
0.03316	120.94	297.26	26.07
0.03596	126.23	325.33	26.24
0.03315	125.26	297.94	26.14
0.02903	125.42	259.06	26.14
0.03600	117.42	326.74	26.42
0.02890	117.42	259.32	26.45
0.03280	122.22	297.88	26.21
0.03253	121.26	296.94	26.11
0.03240	118.06	297.27	26.11
0.03285	123.02	297.41	25.86
0.03285	123.02	297.41	25.86
0.03253	122.22	297.27	25.99
0.03285	122.86	297.34	26.11
0.03275	130.07	297.45	25.31
0.03598	129.59	326.14	25.41
0.02972	129.59	266.87	25.41
0.03285	119.66	297.41	26.05
0.03203	128.47	291.26	24.93
0.03246	123.18	297.17	26.01
0.03254	121.26	297.28	26.71
0.03306	126.39	297.17	25.97
0.03236	118.86	297.13	25.93
0.03285	124.62	297.06	26.09
0.03572	124.78	326.00	26.07
0.03572	124.78	326.00	26.07
0.02851	124.78	255.28	26.07
0.03265	128.15	297.35	26.23
0.03270	125.91	297.11	26.13

## 12. Appendix C

**Table C1:** Data set 3, Lot 1970, Boulder [16]

λ_exp_	ρ	*T_m_*	Δ*T_avg_*	*L_avg_*

(W·m^−1^·K^−1^)	(kg·m^−3^)	(K)	(K)	(mm)
0.034092	123.65	311.644	25.348	25.88
0.033869	123.65	311.580	25.575	25.88
0.034297	123.65	310.525	25.258	25.88
0.022228	123.65	187.612	124.050	25.88
0.019879	123.65	165.123	78.904	25.88
0.018241	123.65	152.626	54.781	25.88
0.016640	123.65	138.698	26.914	25.88
0.014072	123.65	115.118	24.334	25.88
0.015227	123.65	123.751	26.341	25.88
0.017701	123.65	144.457	27.112	25.88
0.020464	123.65	171.285	25.901	25.88
0.023225	123.65	196.712	25.781	25.88
0.025288	123.65	219.507	40.204	25.88
0.027353	123.65	239.605	40.740	25.88
0.028664	123.65	251.520	12.529	25.88
0.029153	123.65	257.607	21.626	25.88
0.031537	123.65	283.486	37.830	25.88
0.031729	123.65	285.112	26.492	25.88
0.034559	123.65	310.495	25.922	25.88
0.034517	123.65	311.269	25.450	25.88
0.036229	123.55	332.098	22.534	25.90
0.032821	123.55	299.623	24.378	25.90
0.032931	123.55	299.774	24.294	25.90
0.033057	123.55	299.548	24.476	25.90
0.033020	123.55	299.507	24.860	25.90
0.032815	123.55	299.801	24.478	25.90
0.032965	123.55	299.789	24.277	25.90
0.012043	123.55	99.167	23.186	25.90
0.011945	123.55	99.176	23.522	25.90
0.012951	123.55	107.831	26.584	25.90
0.013826	123.55	114.948	41.021	25.90
0.017062	123.55	143.289	77.160	25.90
0.015770	123.55	131.860	64.276	25.90
0.018830	123.55	159.188	36.933	25.90
0.018591	123.55	157.940	38.577	25.90
0.019115	123.55	160.370	34.197	25.90
0.024518	123.55	213.970	70.222	25.90
0.025396	123.55	222.192	41.753	25.90
0.024683	123.55	214.957	26.565	25.90
0.027843	123.55	247.856	22.184	25.90

## 13. Appendix D

**Table D1:** Data set 4, SRM 1450 (Lot 1961), Gaithersburg

λ_exp_	ρ	*T_m_*	*L_avg_*

(W·m^−1^·K^−1^)	(kg·m^−3^)	(K)	(mm)
0.03257	131.67	296.28	24.03
0.03610	131.83	327.65	24.01
0.03236	129.27	296.48	24.09
0.02999	129.27	272.61	24.08
0.03560	124.14	327.23	24.46
0.03223	124.30	297.09	24.43
0.03586	134.56	327.47	23.85
0.03257	134.72	296.77	23.86
0.03007	134.56	272.61	23.87
0.03634	145.29	327.49	24.22
0.03299	145.29	296.82	24.21
0.03024	145.77	272.64	24.17
0.03596	134.39	327.52	24.40
0.03270	134.56	297.06	24.38
0.03227	126.71	297.14	24.17
0.03230	122.22	297.03	24.63
0.03645	148.01	327.83	24.18
0.03347	148.01	297.36	24.19
0.03071	148.01	271.77	24.18
0.03291	151.21	296.12	24.03
0.03340	151.21	295.96	24.04
0.03550	122.38	327.92	24.22
0.03227	122.54	297.72	24.18
0.02966	122.54	272.16	24.19
0.03220	129.27	297.01	24.07
0.03632	150.41	327.89	23.99
0.03322	149.77	296.97	24.07
0.03070	149.77	272.70	24.07
0.03358	149.13	296.56	24.42
0.03021	154.74	268.67	24.21
0.03583	122.86	327.61	24.48
0.03233	122.86	297.16	24.49
0.02975	122.86	272.65	24.48
0.03272	145.77	297.35	24.60
0.03025	145.77	272.31	24.61
0.03131	133.11	285.18	24.59
0.03677	152.02	328.29	24.24
0.03335	152.34	297.08	24.21
0.03681	148.97	328.20	24.52
0.03322	148.97	297.11	24.50
0.03270	144.97	297.04	24.02
0.03254	139.52	297.06	24.17
0.03327	152.18	297.06	23.93
0.03299	149.93	296.98	24.05
0.03217	127.35	297.01	24.18
0.03302	135.04	297.04	23.93
0.03265	133.91	297.07	23.95
0.03254	133.91	297.17	23.91
0.03270	136.16	297.10	23.98
0.03655	149.13	327.54	24.26
0.03193	149.13	283.48	24.23
0.03312	149.45	297.11	24.54
0.03612	149.45	322.41	24.02
0.03120	151.53	277.53	24.12
0.03613	139.52	327.49	24.35
0.03280	139.52	297.04	24.38
0.03579	135.84	327.12	24.52
0.03247	135.84	296.71	24.51
0.02929	135.84	266.42	24.51
0.03603	143.37	327.65	24.52
0.03278	143.37	296.98	24.27
0.03021	143.37	272.39	24.28
0.03229	140.96	294.39	24.39
0.03279	134.72	297.03	24.06
0.03680	143.85	327.71	24.35
0.03322	143.85	296.91	24.35
0.03576	135.84	326.99	24.61
0.03256	135.84	296.91	24.57
0.02943	135.84	266.29	24.56
0.03275	138.08	297.16	24.52
0.03004	138.08	272.22	24.52
0.02839	138.08	255.29	24.51
0.03636	149.93	327.92	24.16
0.03325	149.93	297.09	24.12
0.03045	149.93	271.88	24.09
0.03306	141.28	294.39	24.52
0.03341	145.61	297.03	24.46
0.03306	158.90	296.85	24.22
0.03293	146.41	296.94	24.37
0.03610	133.75	327.51	24.20
0.02835	133.59	255.43	24.24
0.03544	133.91	324.95	24.68
0.03244	133.75	296.94	24.72
0.03397	133.59	311.06	24.73
0.03390	151.37	297.01	24.43
0.03690	148.01	327.61	24.67
0.03342	148.17	297.09	24.64
0.02901	148.01	255.33	24.66
0.03262	128.95	297.08	24.58
0.03324	140.64	297.03	24.47
0.03647	144.33	327.66	24.30
0.03314	144.17	297.06	24.33
0.03044	144.17	273.33	24.32
0.03256	137.76	297.12	24.03
0.03015	137.60	271.94	24.06
0.03299	139.36	297.08	23.92
0.03315	147.53	297.13	24.34
0.03167	150.89	282.90	24.17
0.03022	118.06	283.14	24.59
0.03280	145.13	296.96	24.38
0.03642	150.57	327.75	24.53
0.03332	150.57	296.69	24.30
0.03282	148.01	296.91	24.20
0.03357	147.05	297.04	24.37
0.03276	139.04	297.00	24.44
0.03623	144.33	327.23	23.89
0.03311	145.61	297.71	23.86
0.02884	144.97	257.20	23.80
0.03272	156.98	296.98	24.06
0.03269	143.04	297.14	24.57
0.02831	143.21	255.66	24.59
0.03564	141.92	324.58	24.37
0.03338	147.37	297.33	23.83
0.03229	124.78	297.29	23.82
0.03292	142.88	297.23	24.09
0.03257	126.23	297.50	23.80
0.03305	142.56	297.19	24.69
0.03301	139.84	297.31	23.93
0.03618	140.48	326.52	24.03
0.02844	139.68	256.15	23.99
0.03358	151.69	297.08	24.26

## 14. Appendix E

**Table E1:** Data set 5, SRM 1450a (Lot 1958), Gaithersburg

λ_exp_	ρ	*T_m_*	*L_avg_*

(W·m^−1^·K^−1^)	(kg·m^−3^)	(K)	(mm)
0.03168	72.72	296.09	25.51
0.03541	72.72	326.26	25.47
0.03514	72.88	326.64	25.50
0.03155	72.88	296.13	25.58
0.03182	85.22	296.23	25.47
0.03570	85.22	326.93	25.43
0.02995	85.22	279.83	25.51
0.03566	98.83	327.08	25.67
0.03198	98.83	296.28	25.72
0.03680	134.39	326.83	25.31
0.03292	134.39	295.88	25.29
0.03561	95.79	326.84	25.22
0.03182	95.79	296.13	25.23
0.03551	104.44	326.92	25.21
0.03200	104.28	296.17	25.27
0.03521	94.51	327.06	25.31
0.03171	94.67	296.37	25.29
0.03188	105.08	296.46	25.69
0.03515	88.74	327.03	25.97
0.03182	88.74	296.34	25.97
0.02992	88.74	279.17	25.96
0.03159	94.03	296.22	25.93
0.03169	80.89	296.93	25.43
0.03547	97.55	326.94	25.75
0.03177	97.55	296.12	25.77
0.03198	105.24	296.38	25.62
0.03566	108.44	327.44	25.12
0.03198	107.96	296.14	25.18
0.03027	107.96	278.74	25.19
0.03211	111.17	296.33	25.09
0.03211	113.57	296.38	25.30
0.03014	113.57	279.13	25.31
0.03585	116.93	327.37	25.44
0.03204	116.61	296.20	25.50
0.02939	116.45	270.89	25.55
0.03559	108.28	327.48	25.45
0.03220	108.28	296.34	25.57
0.02982	108.28	274.28	25.54
0.03645	128.79	327.37	25.38
0.02949	128.47	271.30	25.46
0.03600	90.66	327.60	26.09
0.03180	90.66	296.08	26.10
0.03573	90.66	327.77	25.91
0.03180	90.66	296.03	25.91
0.02906	90.66	271.14	25.91
0.03187	95.63	295.17	25.15
0.03155	70.48	296.48	25.76
0.02906	68.88	275.42	25.51
0.03586	114.85	327.91	26.17
0.03240	114.85	296.06	26.15
0.03004	114.85	274.49	26.13
0.03210	99.80	296.44	26.16
0.03257	133.11	296.71	25.93
0.03567	85.06	327.30	25.45
0.03181	84.90	296.47	25.52
0.03632	138.08	327.54	26.17
0.03273	138.08	296.44	26.17
0.03022	138.08	272.43	26.16
0.03193	126.07	296.73	26.52
0.02740	126.07	254.14	26.52
0.03537	125.91	327.44	26.57
0.03528	98.19	327.68	25.57
0.03171	98.35	296.96	25.53
0.02904	98.35	271.87	25.52
0.03440	79.77	319.51	26.18
0.03276	127.83	296.86	25.53
0.02907	128.15	263.15	25.47
0.03600	127.83	327.33	25.47
0.03664	128.95	327.88	25.27
0.02897	128.47	264.45	25.43

## 15. Appendix F

**Table F1:** Data set 6, Lot 1958 (SRM 1450a), Boulder [16]

λ_exp_	ρ	*T_m_*	Δ*T_avg_*	*L_avg_*

(W·m^−1^·K^−1^)	(kg·m^−3^)	(K)	(K)	(mm)
0.033329	127.07	302.228	24.890	25.900
0.033258	127.07	302.339	24.583	25.900
0.012750	127.07	103.763	16.871	25.900
0.014503	127.07	118.145	24.033	25.900
0.020024	127.07	170.605	22.939	25.900
0.025688	127.07	225.069	23.501	25.900
0.030726	127.07	274.869	21.202	25.900
0.030362	127.07	273.300	24.848	25.900
0.026172	127.07	229.536	38.447	25.900
0.028235	127.07	251.432	10.640	25.900
0.030653	127.07	273.927	22.808	25.900
0.027465	127.07	238.832	10.469	25.900
0.027301	127.07	238.832	10.521	25.900
0.030787	127.07	273.080	25.019	25.900
0.012791	127.07	100.786	20.344	25.900
0.026050	127.07	224.288	22.180	25.900
0.031070	127.07	272.932	24.482	25.900
0.021169	127.07	178.292	38.475	25.900
0.021262	127.07	178.281	38.497	25.900
0.012639	127.07	98.722	15.392	25.900
0.012541	127.07	98.662	15.537	25.900
0.014983	127.07	122.295	24.321	25.900
0.015316	127.07	122.206	24.450	25.900
0.020538	127.07	172.210	25.414	25.900
0.020571	127.07	172.252	25.373	25.900
0.012155	127.07	97.153	13.778	25.900
0.015381	127.07	121.322	25.955	25.900
0.018060	127.07	147.064	25.210	25.900
0.023186	127.07	197.820	24.902	25.900
0.028284	127.07	248.004	25.249	25.900
0.033292	127.07	297.346	23.274	25.900
0.033122	127.07	297.413	23.395	25.900
0.033696	127.11	297.316	23.250	25.892
0.033342	127.11	297.335	23.322	25.892
0.034267	147.23	297.392	23.203	25.892
0.033872	147.23	297.478	23.381	25.892
0.033838	147.23	297.440	23.239	25.892
0.033852	147.23	297.394	23.151	25.892
0.018661	147.53	146.975	25.605	25.839
0.023791	147.43	197.578	24.966	25.856
0.028968	147.33	248.093	25.027	25.873
0.032548	104.90	297.458	23.127	25.892
0.027048	104.97	247.988	25.238	25.873
0.012071	105.17	100.317	15.522	25.825
0.016988	105.11	147.315	24.834	25.839
0.022061	105.04	198.114	24.484	25.856
0.012303	114.78	99.268	10.537	25.824
0.017248	114.71	147.335	24.962	25.839
0.022677	114.64	197.742	25.198	25.856
0.027591	114.56	247.462	18.635	25.873
0.032860	114.48	297.431	23.142	25.892
0.032274	114.48	297.580	22.884	25.892
0.032434	114.48	297.657	22.957	25.892
0.038204	114.39	348.905	24.763	25.912
0.033631	116.85	310.492	24.657	25.367
0.017078	117.11	147.474	25.253	25.310
0.022381	117.03	197.968	25.585	25.326
0.027274	116.97	245.093	20.305	25.340
0.032270	116.87	297.982	23.150	25.362
0.032479	114.43	298.031	23.266	25.902

## 16. Appendix G

**Table G1:** Data sets 7–9 SRM 1450b(II) (Lots 1980 and 1981) [16]

λ_exp_	ρ	*T_m_*	Δ*T_avg_*	*L_avg_*	Subset^a^

(W·m^−1^·K^−1^)	(kg·m^−3^)	(K)	(K)	(mm)	
0.036548	128.67	310.414	24.293	25.367	1
0.036397	121.14	310.475	24.477	25.367	1
0.038174	121.12	325.347	24.959	25.372	1
0.014396	121.46	118.529	25.257	25.301	1
0.015604	121.44	128.685	25.236	25.304	1
0.016936	121.43	138.875	25.147	25.307	1
0.018563	121.41	152.222	31.353	25.312	1
0.013279	121.47	108.488	25.465	25.298	1
0.020412	121.38	169.315	25.330	25.317	1
0.021587	121.37	179.396	25.106	25.320	1
0.019147	121.40	159.119	25.283	25.314	1
0.022760	121.35	189.589	25.249	25.324	1
0.023864	121.33	199.624	25.238	25.327	1
0.024970	121.32	209.703	25.265	25.330	1
0.026063	121.30	219.809	25.188	25.334	1
0.027150	121.28	229.856	25.180	25.337	1
0.028241	121.27	239.919	25.125	25.341	1
0.029313	121.25	249.989	25.091	25.344	1
0.032047	121.21	275.173	25.171	25.354	1
0.034865	121.16	300.275	25.033	25.363	1
0.035592	144.58	300.350	24.959	25.363	1
0.038673	144.53	325.501	24.975	25.372	1
0.013812	144.95	108.427	25.427	25.298	1
0.014864	144.93	118.465	25.346	25.301	1
0.016023	144.92	128.455	25.413	25.304	1
0.017394	144.90	138.832	25.535	25.307	1
0.019293	144.87	152.030	31.813	25.312	1
0.020186	144.86	159.258	25.367	25.314	1
0.021339	144.84	169.350	25.275	25.317	1
0.022481	144.82	179.437	25.127	25.320	1
0.023618	144.81	189.646	25.320	25.324	1
0.024546	144.79	199.648	25.208	25.327	1
0.025668	144.77	209.706	25.201	25.330	1
0.027020	144.75	219.673	25.147	25.334	1
0.027845	144.73	229.849	25.100	25.337	1
0.028829	144.71	239.827	25.026	25.341	1
0.029958	144.69	249.958	25.142	25.344	1
0.032754	144.63	275.074	25.134	25.353	1
0.027723	144.73	229.739	25.213	25.337	1
0.036986	142.47	310.383	24.359	25.367	2
0.036273	133.80	310.533	24.767	25.367	2
0.013643	136.93	106.661	25.667	25.298	2
0.016469	136.89	130.224	28.492	25.305	2
0.018283	136.86	147.607	25.258	25.310	2
0.024048	136.77	197.923	25.303	25.326	2
0.027159	136.72	225.615	24.164	25.336	2
0.029684	136.68	249.077	20.855	25.344	2
0.032343	136.63	274.174	21.456	25.353	2
0.034946	136.58	297.922	22.637	25.362	2
0.036974	136.54	316.269	23.579	25.369	2
0.039348	136.50	335.955	23.736	25.377	2
0.039633	136.50	336.053	23.932	25.377	2
0.039588	136.50	335.935	23.672	25.377	2
0.034962	136.58	297.905	22.547	25.362	2
0.029541	136.68	249.305	20.899	25.344	2
0.029577	136.68	249.301	20.885	25.344	2
0.034849	136.58	297.976	22.670	25.362	2
0.039536	136.50	336.156	24.118	25.377	2
0.036848	136.56	306.891	24.658	25.365	2
0.036513	136.56	306.893	24.662	25.365	2
0.037563	136.54	319.114	48.909	25.370	2
0.037593	136.54	319.119	48.921	25.370	2
0.039411	136.51	331.269	24.301	25.375	2
0.039446	136.51	331.320	24.240	25.375	2
0.038839	136.51	331.347	24.242	25.375	2
0.039256	135.50	331.434	24.433	25.565	2
0.038573	137.55	331.246	24.238	25.184	2
0.013302	136.93	106.249	25.698	25.298	2
0.014984	136.91	118.643	25.070	25.301	2
0.015028	136.91	118.417	25.460	25.301	2
0.014385	136.91	118.308	25.413	25.301	2
0.017440	136.88	139.023	25.192	25.307	2
0.020217	136.83	164.284	25.132	25.315	2
0.022380	136.80	184.476	25.267	25.322	2
0.025070	136.75	209.590	24.947	25.330	2
0.027937	136.71	234.998	25.141	25.339	2
0.029693	136.68	249.239	20.953	25.344	2
0.029472	136.68	249.750	21.901	25.344	2
0.030094	135.66	249.882	22.104	25.335	2
0.034471	122.3	297.005	24.208	25.22	3
0.035546	133.5	302.648	23.937	25.59	3
0.034453	128.6	296.852	24.068	25.31	3
0.038113	128.8	324.852	22.521	25.27	3
0.029552	128.7	254.858	21.816	25.29	3
0.038020	120.6	324.391	22.840	25.39	3
0.034457	120.4	297.238	23.959	25.68	3
0.029734	120.8	256.466	21.315	25.35	3
0.038508	136.7	325.666	21.977	25.53	3
0.034713	136.8	297.394	23.971	25.52	3
0.034332	112.6	297.279	23.485	25.25	3
0.029204	113.3	254.585	21.864	25.26	3
0.038495	141.5	327.088	22.000	25.72	3
0.034853	141.5	297.232	24.352	25.72	3
0.030152	141.5	257.549	21.820	25.73	3
0.037063	137.8	314.415	23.701	25.79	3
0.034003	137.4	290.535	24.416	25.01	3
0.032325	132.9	278.924	20.650	25.69	3
0.035928	133.1	305.305	23.507	25.64	3
0.037090	119.4	318.131	22.998	25.40	3
0.035167	119.4	302.742	24.149	25.40	3
0.031182	133.7	268.516	21.761	25.56	3
0.038337	135.9	326.295	22.592	25.60	3
0.035371	136.9	302.494	23.976	25.51	3
0.038706	141.4	327.142	22.613	25.75	3
0.034994	141.6	297.196	24.280	25.73	3
0.030133	141.4	258.298	22.449	25.74	3
0.034277	112.5	296.979	23.730	25.27	3
0.033697	133.1	289.369	23.591	25.28	3
0.034901	131.3	296.968	23.931	25.79	3
0.035893	131.3	304.192	23.516	25.80	3
0.034776	126.4	296.910	24.354	25.43	3
0.034843	128.4	296.767	23.979	25.30	3
0.031894	128.6	274.943	19.929	25.26	3

a Subset 1 = Boulder, Lot 1980

a Subset 2 = Boulder, Lot 1981

a Subset 3 = Gaithersburg, Lot 1981; Ref. [16] does not identify from which guarded-hot-plate apparatus the data were obtained.

**Table G2:** Outlier data removed from analysis SRM 1450b(II) (Lots 1980 and 1981) [16]

λ_exp_	ρ	*T_m_*	Δ*T_avg_*	*L_avg_*	Subset^a^

(W·m^−1^·K^−1^)	(kg·m^−3^)	(K)	(K)	(mm)	
0.033846	133.1	313.517	22.037	25.280	3

a Subset 3 = Gaithersburg, Lot 1981; Ref. [16] does not identify from which guarded-hot-plate apparatus the data were obtained.

## 17. Appendix H

**Table H1:** Data set 10, SRM 1450c(II) (Lot 1996)

λ_exp_	ρ	*T_m_*	Δ*T_avg_*	*L_avg_*

(W·m^−1^·K^−1^)	(kg·m^−3^)	(K)	(K)	(mm)
0.031610	155.77	280.149	20.003	25.51
0.031845	158.46	280.148	20.002	25.16
0.032171	165.97	280.150	20.000	25.55
0.033138	156.06	295.149	20.002	25.42
0.032891	157.14	295.145	20.009	25.39
0.033574	163.22	295.151	19.998	25.15
0.034545	151.53	310.149	20.001	25.88
0.035484	160.08	310.148	20.004	25.29
0.035148	162.58	310.150	20.001	25.27
0.036104	149.38	325.153	19.994	25.09
0.036925	159.18	325.151	19.999	25.07
0.036615	161.46	325.155	19.990	25.42
0.037885	154.83	340.149	20.004	25.39
0.038090	156.53	340.149	20.003	25.01
0.038396	164.91	340.149	20.001	25.35

## 18. Appendix I

**Table I1:** Data set 11, SRM 1450d (Lot 2009) [18]

λ_exp_	ρ	*T_m_*	Δ*T_avg_*	*L_avg_*

(W·m^−1^·K^−1^)	(kg·m^−3^)	(K)	(K)	(mm)
0.031123	113.51	279.998	25.003	25.93
0.030961	118.50	280.001	24.996	25.80
0.030898	121.25	279.998	25.004	25.56
0.032450	114.35	295.001	24.997	26.02
0.032491	119.01	294.998	25.003	25.82
0.032614	121.45	295.001	24.997	25.80
0.034089	115.79	310.001	25.000	26.13
0.034283	118.68	310.001	24.999	25.76
0.034223	122.40	310.001	25.000	25.76
0.035723	115.67	324.999	25.003	25.85
0.035968	118.85	324.999	25.002	26.00
0.035988	123.24	325.001	25.002	25.73
0.037524	115.84	339.999	25.004	25.98
0.037605	119.14	340.000	25.001	25.78
0.037817	123.80	339.999	25.001	25.85

## 19. Appendix J – Addendum on NBS Fibrous Glass Board and SRMs 1450–1450d

In the course of reviewing this manuscript, it was suggested that a historical overview of this particular SRM be prepared addressing the impact on external measurement programs. This addendum provides supplementary information on the early history of the NBS measurement program for fibrous glass board, selection of the original source material, conversion of the measurement service to become part of the NIST SRM program, and the resulting impact.

### J1. Early History

In 1951, Gilbo [60] described a series of guarded-hot-plate measurements using corkboard “standards” that had been sent to the U.S. Bureau of Standards in 1947 and 1948 for measurement. The standard specimens were utilized to investigate the performance of existing thermal conductivity equipment and new hot plate designs. The completed guarded-hot-plate apparatus was described by Zabawsky [61] in 1957. The operation of the apparatus was investigated by comparisons with NBS calibration specimens of fiberboard, corkboard, fibrous-glass blanket and fibrous-glass board. The agreement of their hot-plate apparatus and the NBS measurements was within ±1.3 % [61] over a temperature range of 15.6 °C to 37.8 °C (60 °F to 100 °F).

In 1959, the National Bureau of Standards reported the establishment of a new activity for thermal conductivity reference specimens. The original account, published in the “Research Highlights of the National Bureau of Standards for Fiscal Year 1959,” is given [62].

#### Thermal Conductivity Reference Specimens

For several years, various laboratories have submitted to the Bureau specimens of insulating materials for an accurate determination of thermal conductivities. Laboratories use these specimens as references for calibrating thermal conductivity measuring apparatus. Requests for this service have been increasing. In order to satisfy such needs more quickly, and to avoid problems arising from use of unsuitable materials, two materials having satisfactory characteristics of homogeneity and stability (glass-fiber board and gum rubber) were selected and stocked. Reference specimens can be prepared from this stock and the thermal conductivity measured at the specified temperatures. Such services are available under the cost fee schedule to governmental, industrial, and university laboratories.

The measurement service was described in more detail for customers as “Determination for calibration purposes of the thermal conductivity of a selected pair of specimens, by means of guarded hot plate apparatus (conforming to ASTM C177) for mean temperatures between 0 and 130 °F^6^ (ordinarily 0, 30, 75, and 130 °F), per determination at one mean temperature.” Converting these mean temperatures to kelvin yields 255 K, 272 K, 297 K, and 328 K, respectively, which correlate well with the discrete levels of temperatures displayed in Figs. 8b, 8d, 8h, and 8j.

The calibration program covered a wide range of thermal conductivities applicable to the operating limits of the guarded hot plate apparatus, from 0.034 W·m^−1^·K^−1^ (0.24 Btu·in.·h^−1^·ft^−2^·°F^−1^) for fibrous glass board to 0.16 W·m^−1^·K^−1^ (1.1 Btu·in.·h^−1^·ft^−2^·°F^−1^) for gum rubber. Also available, were specimens of silicone rubber 0.36 W·m^−1^·K^−1^ (2.5 Btu·in.·h^−1^·ft^−2^·°F^−1^). The specimens were available in sizes of 203 mm to 457 mm by 12.7 mm to 25.4 mm thick (8 to 18 inches square, ½ or 1 inch thick).

The first test record for “NBS Fibrous Glass Board” was documented in the hot-plate laboratory logbook on March 11, 1958; the final measurement under the calibration program, November 29, 1977. In December 1977, T. W. Watson, under the direction of F. J. Powell and M. C. I. Siu, collected and organized the hand recorded thermal conductivity data on NBS fibrous glass board derived from measurements made on the NBS 203.2 mm guarded-hot-plate apparatus (described in Table 5). The collated data were tabulated in a form suitable for statistical analysis (which are reproduced in Appendices A, B, D, and E from the original computer printouts). The statistical analysis was completed in January 1978 by M. C. I. Siu, B. A. Peavy and H. H. Ku using the computer program OMNITAB [63]. The results have been described by Siu [7], as documented in this paper.

### J2. Initial 1958 Lot of NBS Fibrous Glass Material

The first lot of fibrous glass insulating material, lot 1958, was procured from a commercial source. The purchase order, dated January 1958 and in possession of the first author, requests a quantity of 200 square feet of 5 pound per cubic feet density of Aerocor^7^; “with smooth flat parallel faces, in pieces 24 in. × 24 in. × 1 in.” The cost was $125.00. The insulating material “Aerocor” was introduced in 1950 [64] and described as “fiberglass fibers lightly bonded, with a thermosetting resin, into blankets….” The articles states that Aerocor has been “used as thermal and sound insulation in boilers, home freezer units, air conditioners, incubators, and transportation equipment [64].” The *Handbook of Material Trade Names* [65] records the material as a preformed insulation, “glass wool bonded with a resin to predetermined thickness and density; used in roll blanket form as residential, automotive, aircraft, and industrial thermal and acoustical insulation.” As stated by handwritten notes in NBS hot-plate logbook #6, the thermal insulation material was covered under military specification MIL-I-16022B (now cancelled), “Insulation Felt, Thermal, Fibrous Glass, Flexible”. The specification required ranges of properties include the following: average of fibers to be 0.00010 in. to 0.00055 in., with no fiber greater than 0.00060 in. Handwritten notes in the hot-plate logbook #6 state that the “glass size was 0.00019 in.; in range of 0.00018 in. to 0.00025 in. diameter with phenolic binder”.

### J3. Transition to the NIST SRM Program

It is interesting to note that NBS Circular 552, 2^nd^ Edition [66], published in 1957 (one year prior to the establishment of the thermal insulation calibration program in 1958) lists only standard samples of reference materials issued with respect to their chemical composition. The document states that “Information on certain physical standards, individually certified and intended primarily for the calibration of instruments, that were formerly included in the Circular, does not appear in this edition.” Evidently, the distinction between the two programs was whether the measured property of interest was the analysis of a chemical composition or the determination of a physical property. This constraint would seem to have been relaxed in subsequent years, allowing the inclusion of the thermal insulation measurement properties in the SRM program.

Considerable insight on the development of thermal conductivity reference standards was obtained from a limited distribution paper (dated 1962) on “Thermal Conductivity Reference Standards” by H. E. Robinson [67]. Robinson understood two requirements as paramount for realization of a reference material having absolute values of thermal conductivity for broad applications: stability of conductivity and uniformity. He classified thermal conductivity reference materials into one of three categories, as follows.

1. *Materials Insufficiently Uniform to Compel Need for Measuring Each Reference Specimen*: Such materials are utilized, Robinson reasoned, because “there is no better alternative material.” An example is the semi-rigid fibrous-glass insulating board furnished by NBS for checking ASTM C177 hot plate equipment or calibrating comparative apparatus^8^ for measuring thermal conductivity. Although such materials may be excellent in many respects, including stability under well-defined conditions, the disadvantage is that individual measurements are uneconomical.
2. *Stocked Materials of Proven Uniformity – Batch Samples*: Robinson defined this category as materials shown to be sufficiently uniform in thermal conductivity within a batch or lot so that measurement made on a relatively few specimens are sufficient to characterize the conductivity of the entire batch. Considerable exploratory work, however, was necessary to assure that the lot was satisfactory for this purpose. If the lot was large enough in number and the demand sufficiently high, the cost per specimen could be quite moderate.
3. *Materials of High Purity*: For this category, Robinson states that chemical elements are expected to have unique thermal conductivities, as well as other properties, which may be defined as their limiting values as impurities are reduced. Thermal conductivity reference materials of this kind are advantageous because once the conductivity of the material is adequately determined, reference specimens could be obtained from any source capable of providing the specified purity.

In retrospect, categories one and two in Robinson’s outline above became the path toward a thermal insulation standard reference material. The NBS measurement program was followed by procurement of individual batches or lots of semi-rigid fibrous-glass insulating materials. Subsequent NBS researchers, at the request of ASTM Sub-Committee C16.30, analyzed the thermal conductivity measurement data from the batches in order to determine if a particular batch was, in fact, sufficiently uniform for development as a standard reference material. These materials have become what are now known as the 1450 series of NIST SRMs.

### J4. Impact on Measurement Programs

As described in Sec. 3, Standard Reference Material 1450 was originally issued to the public in the summer of 1978 (Fig. 1). Transaction data, documented by the NIST Office of Reference Materials (ORM) for SRMs 1450–1450d, are summarized in Table J1, which provides totals for the number of SRM units issued, by designation, and includes international procurements. Historical transactions totals for SRMs 1450 and 1450a are currently unavailable, although Siu [7] records that over 300 pairs were issued under the preceding calibration program. Regrettably, the total for SRM 1450b is also incomplete. For 1450c, the historical sales rate ranged from 15 to 35 units per year [15]. The average rate for 1450d is currently about 40 units per year. As evident from the international distribution of units (Table J1), the SRM 1450b–1450d artifacts have been utilized and accepted world-wide.

**Table J1:** Number of units issued for SRMs 1450–1450d, Fibrous Glass Board

Designation	Number issued	International distribution^a^
1450	Unavailable^b^	—
1450a	Unavailable^b^	—
1450b	164^c^	CA, ES, GB, IT, JP, KR, NL, PT
1450c	359	AU, CA, DE, DK, ES, JP, KR, TR
1450d	92	AU, CA, CN, IN, KR, LU, SI, TH, ZA

a Countries designated by their two-letter country code [68].

b Over 300 pairs issued as part of calibration program [7].

c Incomplete total due to unavailable records.

The extent to which these thermal insulation reference materials have influenced external measurement programs was examined by conducting a literature review of technical papers, trade journals, and the U.S. Federal Register. A search of the relevant literature databases revealed a large number of technical papers describing measurement programs that have utilized “NBS Fibrous Glass” or “SRMs 1450–1450d.” Table J2 presents an overview of the technical papers that document the utilization of these reference materials from 1967 to the present. The applications represented are grouped in one of four categories – instrument calibration, instrument verification, inter-laboratory comparison, or other miscellaneous properties measured or reported by researchers. Additional information on a particular application is provided, where applicable, in the footnotes for Table J2.

**Table J2:** Researchers utilizing NBS Fibrous Glass Board or SRMs 1450–1450d

Designation	Instrument celibration	Instrument verification	Inter-laboratory comparison	Miscellaneous
NBS Fibrous Glass Board	[69–70]^b^	[71]^c^–[72]^e^	—	—
1450	[73–77]^a^ [78–80]^b^	[81]^f^	—	[82]
1450a	—	[83]^g^	—	—
1450b	[84]^a^ [85–90]^b^	[91–93]^d^	—	[94–95]^h^ [96–98]^i^
1450c	[99–102]^b^	[103–105]^c^ [106]^j^	[107]	[108]
1450d	—	—	—	—

a Heat flux sensor

b Heat flow meter apparatus (ASTM C 518)

c Guarded hot plate apparatus (ASTM C 177)

d Unguarded thin-heater apparatus (ASTM C 1114)

e Bi-guarded hot plate/heat flow meter apparatus

f Calibrated hot box

g Guarded thermal hot plate

h Radiative properties

i Coupled conduction and radiation model

j Transient plane source method

Table J2 indicates that the majority of the technical papers involve the calibration of heat flux transducers either directly or as part of the heat-flow-meter apparatus. A large number of papers involve the verification of measurement results from absolute thermal conductivity instruments including: the guarded hot plate (and variations of the method), hot box, and transient plane source. Standard Reference Material 1450b has been utilized for the measurement of radiative properties and resulting models. Standard Reference Material 1450c was used as part of a comparison between guarded-hot-plate facilities at NIST and the National Research Council Canada.

The papers summarized in Table J2 cover a timeframe of 46 years from 1967 to 2013. Because of the advocacy of the ASTM Sub-Committee C16.30 on Thermal Measurements, it would be expected that many of the papers utilizing SRMs 1450–1450b were from U.S. industry and government organizations. Many of these technical papers were associated with measurement issues for insulating materials and energy conservation efforts in buildings during the 1970s, 1980s, and early 1990s. In general, the papers deal with measuring heat flows through walls, roofs or new insulation products. As noted in Table J2, the applications for SRM 1450c reflect much more usage by the international testing community for a wide range of applications: from fire-resistant textiles [99–100] to nanoporous silica cryogels [104] to the thermal properties of structural components of the BepiColumbo probe [105]. The breadth and number (38) of these documented technical investigations utilizing SRMs 1450–1450d were unexpected. In a broader context, NIST SRMs 1450–1450d have been summarized as part of a review of reference materials for thermophysical properties [108–109].

The literature review also revealed that the Federal Register [82] listed SRM 1450 as a requisite reference program for NVLAP Code/ASTM Test Methods C 177 [1] and C 518 [2]. Laboratories participating in the accreditation program were recommended to maintain a “uniform batch of test specimens for more frequent checks of its performance [82]” (i.e. check standards) selected from SRM 1450. The NVLAP methodology for proficiency testing was/is to provide participating laboratories proficiency specimens having properties not known in advance. For example, in Round 6, June 1981 [13], NVLAP utilized a nominal 25.4 mm thick, 64 kg·m^−3^ foil-faced fibrous-glass board for test methods C 177 and C 518. This particular material was fabricated from a separate lot of material than those described in this paper.

It should be mentioned that a comprehensive international comparison of guarded-hot-plate and heat-flow-meter apparatus utilized a high-density fibrous-glass board (bulk density about 164 kg·m^−3^ and thickness about 25.4 mm) for comparison measurements. The original test plan and materials are described by Powell and Bales [110]. Materials were circulated to groups of countries by geographic location and common types of apparatus. It is important to emphasize that the lot of high-density material, although quite similar to the SRM 1450 series (particularly 1450c), was an entirely separate lot than those described in this paper. The results for this material for the guarded-hot-plate data were summarized by Smith [111] utilizing the functional form represented by Model 4 in the text. The least-squares values (divided by 1000 for consistency with units given in Table 11) found for the coefficients were [111]:

*a*_0_ = 9.587×10^−3^ W·m^−1^·K^−1^
*a*_1_ = 2.65×10^−5^ W·m^2^·K^−1^·kg^−1^
*a*_2_ = 4.57×10^−5^ W·m^−1^·K^−2^
*a*_3_ = 2.552×10^−10^ W·m^−1^·K^−4^

which are, for the most part, in reasonably good agreement with the results of Table 11 (Data set 7). The temperature coefficient, *a*_2_, is about one-half the values in Table 11.

## 20. Glossary

*a_i_*: regression coefficients for multilinear thermal conductivity models
*A*: meter area of the guarded-hot-plate (m^2^)
ANOVA: analysis of variance
ASTM: American Society for Testing and Materials (now ASTM International)
BIC: Bayesian Information Criteria
CIPM: International Committee for Weights and Measures
*e*: exponential function
*f*: function
GUM: “*Guide to the Expression of Uncertainty in Measurement*”
*i*: index
ISO: International Organization of Standardization
*k*: coverage factor multiplier for the expanded uncertainty usually in the range of 2 to 3
*L*: (in-situ) thickness of guarded-hot-plate test specimen (m)
*L_avg_*: (in-situ) average thickness of specimen pair (m)
*L_i_*: overall linear dimensions of specimen panel (m)
*m*: mass of specimen (kg)
MRA: Mutual Recognition Arrangement
*n*: sample size
NIST: National Institute of Standards and Technology
NVLAP: National Voluntary Laboratory Accreditation Program
ORM: Office of Reference Materials (formerly Standard Reference Materials Program)
*p*: number of model parameters in BIC statistic (Eq.(18))
*p_a_*: ambient chamber pressure (kPa)
*Q*: heat flow rate through meter area of guarded-hot-plate test specimen (W)
*Q_g_*: lateral (i.e. radial) heat flow rate across the guard gap (W)
*Q_e_*: edge heat flow (W)
*R*_0_: certified values of thermal resistance (m^2^·K·W^−1^)
REFPROP: REFerence fluid PROPerties (NIST Standard Reference Database 23)
REMCO: ISO Committee on Reference Materials
RES: residuals from model fit
RESSD: residual standard deviation from model fit
SRM: Standard Reference Material
*t*, *t*-statistic: statistic for testing whether a regression coefficient is significantly different from zero
*T*: (mean) temperature (K)
TGA: thermogravimetric analysis
*T_a_*: ambient air temperature (K)
*T_c_*: (average) cold-plate temperature (K)
*T_h_*: hot-plate temperature (K)
*T_m_*: mean specimen temperature average of two opposite surface temperatures (K)
*u_c_*: combined standard uncertainty, *k* = 1
*u_c,rel_*: relative combined standard uncertainty, *k* = 1 (dimensionless)
*u_i_* standard uncertainty for a particular quantity: *k* = 1
*U_rel_* relative expanded uncertainty: *k* = 2 (dimensionless)
*V*: volume of specimen (m^3^)
*x*(*i*): independent variable
α: ratio of temperature differences
$\hat{\beta }(i)$: regression coefficient estimate for *x*(*i*)
Δρ: range of bulk density for particular SRM lot (kg·m^−3^)
Δ*T*: temperature difference (K)
Δ*T_avg_*: mean temperature difference across specimen pair (K)
λ(ρ*T*): thermal conductivity predicted from multilinear models (W·m^−1^·K^−1^)
λ_exp_: experimental thermal conductivity (W·m^−1^·K^−1^)
ρ: bulk density (kg·m^−3^)

**Additional subscripts**
1: top cold plate or specimen
2: bottom cold plate or specimen

**Additional superscript**
−: denotes sample mean
^: denotes estimate of parameter

## References

[1] (2013). ASTM Standard C177-10, Test Method for Steady-State Heat Flux Measurements and Thermal Transmission Properties by Means of the Guarded-Hot-Plate Apparatus, Annual Book of ASTM Standards.

[2] (2013). ASTM Standard C518-10, Test Method for Steady-State Thermal Transmission Properties by Means of the Heat Flow Meter Apparatus, Annual Book of ASTM Standards.

[3] (2013). ASTM Standard C1363-10, Test Method for Thermal Performance of Building Materials and Envelope Assemblies by Means of a Hot Box Apparatus, Annual Book of ASTM Standards.

[4] Lide DR (2001). A Century of Excellence in Measurements Standards, Technology: A Chronicle of Selected NBS/NIST Publications, 1901–2000.

[5] Zarr RR (2001). A History of Testing Heat Insulators at the National Institute of Standards and Technology. ASHRAE Transactions.

[6] McAllister JD (1982). ASTM Insulation Standards and Energy Conservation. ASTM Standardization News.

[7] Siu MCI, McElroy DL, Tye RP (1980). Fibrous Glass Board as a Standard Reference Material for Thermal Resistance Measurement Systems. Thermal Insulation Performance.

[8] ASTM Subcommittee C16.30 (1974). Reference Materials of Low Thermal Conductivity, Appendix in Heat Transmission Measurements in Thermal Insulations. ASTM STP.

[9] Tye RP, ASTM Subcommittee C16.30 (1978). Reference Materials for Insulation Measurement Comparisons, Thermal Transmission Measurements of Insulation. ASTM STP.

[10] Federal Register (1976). Procedures for a National Voluntary Laboratory Accreditation Program.

[11] Bryson JO (1981). Thermal Insulation Laboratory Accreditation Program. ASHRAE SP.

[12] Kirkpatrick D, Horlick J, Hust JG (1983). Proficiency Testing for Thermal Insulation Materials in the National Voluntary Laboratory Accreditation Program. Thermal Conductivity.

[13] Horlick J, Berger HW (1985). NVLAP and the Thermal Insulation Proficiency Testing Program. Journal of Thermal Insulation.

[14] Horlick J, Knab L, Gaal DS, Gaal PS (2010). Thermal Conductivity Proficiency Testing Results – Nineteen NVLAP Proficiency Testing Rounds from 1986 Through 2004. Thermal Conductivity 30/Thermal Expansion.

[15] Zarr RR, Koenig JR, Ban H (2008). Status of NIST Thermal Insulation Reference Materials. Thermal Conductivity 29/Thermal Expansion.

[16] Hust JG (1985). Standard Reference Materials: Glass Fiberboard SRM for Thermal Resistance.

[17] Zarr RR (1997). Standard Reference Materials: Glass Fiberboard SRM 1450c for Thermal Resistance from 280 K to 340 K.

[18] Zarr RR, Harris AC, Roller JF, Leigh SL (2011). Standard Reference Materials: SRM 1450d, Fibrous-Glass Board, for Thermal Conductivity from 280 K to 340 K.

[19] Grant JA, Shand EB (1958). Fibrous Glass, Section 4 in Glass Engineering Handbook.

[20] Mohr JG, Rowe WP (1978). Fiber Glass.

[21] Loewenstein KL (1993). The Manufacturing Technology of Continuous Glass Fibers.

[22] Varshneya AK (1994). Fundamentals of Inorganic Glasses.

[23] Wallenberger FT, Bingham PA (2010). Fiberglass and Glass Technology.

[24] Yolken HT (1975). The National Standard Reference Materials Program in the USA.

[25] Smith DR, Hust JG, Hust JG (1983). Effective Thermal Conductivity of Glass-Fiber Board and Blanket Standard Reference Materials. Thermal Conductivity.

[26] Zarr RR, Thomas WC, Kiss LI, St-Georges L (2013). Initial Measurement Results of the NIST 500 mm Guarded-Hot-Plate Apparatus Under Automated Temperature and Pressure Control. Thermal Conductivity 31/Thermal Expansion.

[27] Hust JG, Callahan JE, Sullivan SA, Yarbrough D (1988). Specific Heat of Insulations. Thermal Conductivity.

[28] Steger HF (2002). Twenty-five years of international collaboration in reference materials via ISO/REMCO. Accreditation and Quality Assurance.

[29] ISO Guide 30: 1992 (1992). Terms and definitions used in connection with reference materials.

[30] ISO Guide 31: 2000 (2000). Reference materials – Contents of certificates and labels.

[31] ISO Guide 34: 2009 (2009). General requirements for the competence of reference material producers.

[32] ISO Guide 35: 2006 (2006). Reference materials – General and statistical principles for certification.

[33] Taylor BN, Kuyatt CE (1994). Guidelines for Evaluating and Expressing the Uncertainty of NIST Measurement Results.

[34] BIPM (2008). Evaluation of measurement data – Guide to the expression of uncertainty in measurement. JCGM.

[35] May W, Parris R, Beck C, Fassett J, Greenberg R, Guenther F, Kramer G, Wise S, Gills T, Colbert J, Gettings R, MacDonald B (2000). Definitions of Terms and Modes Used at NIST for Value-Assignment of Reference Materials for Chemical Measurements.

[36] Bruce SS (2006). The Quality System for NIST Measurement Services.

[37] NIST QM-I (2013). Version 8, NIST Quality Manual for Measurement Services.

[38] ISO/IEC 17025:2005(E) (2005). General requirement for the competence of testing and calibration laboratories.

[39] Van Dusen MS (1920). The Thermal Conductivity of Heat Insulators, Transactions. American Society of Heating and Ventilating Engineers.

[40] Siu MCI, Bulik C (1981). National Bureau of Standards line-heat-source guarded-hot-plate apparatus. Review of Scientific Instruments.

[41] Smith DR, Hust JG, Van Poolen LJ (1982). A Guarded-Hot-Plate Apparatus for Measuring Effective Thermal Conductivity of Insulations between 80 K and 360 K. NBSIR.

[42] Powell FJ, Rennex BG (1982). NBS Line-Heat-Source Guarded-Hot-Plate for Thick Materials.

[43] Zarr RR, Leber DD, Gaal DS, Gaal PS (2010). Evaluation of Thermal Insulation Materials for NIST SRM 1450d, Fibrous-Glass Board. Thermal Conductivity 30/Thermal Expansion.

[44] ASTM Standard C 1058/C1058M-10 (2012). Standard Practice for Selecting Temperatures for Evaluating and Reporting Thermal Properties of Thermal Insulation, Annual Book of ASTM Standards.

[45] ASTM Standard C 1045-07 (2012). Standard Practice for Calculating Thermal Transmission Properties Under Steady-State Conditions, Annual Book of ASTM Standards.

[46] Rennex B (1983). Error Analysis for the National Bureau of Standards 1016 mm Guarded Hot Plate. Journal of Thermal Insulation.

[47] Veloroutes.org http://veloroutes.org/elevation/.

[48] Vellman PF, Welsch RE (1981). Efficient Computing of Regression Diagnostics. The American Statistician.

[49] (2012). NIST/SEMATECH e-Handbook of Statistical Methods.

[50] (2012). NIST/SEMATECH e-Handbook of Statistical Methods.

[51] (2012). NIST/SEMATECH e-Handbook of Statistical Methods.

[52] (2012). NIST/SEMATECH e-Handbook of Statistical Methods.

[53] (2012). NIST/SEMATECH e-Handbook of Statistical Methods.

[54] Miller AJ (1990). Subset Selection in Regression.

[55] Smith AFM (2006). Model Selection: Bayesian Information Criteria, in Encyclopedia of Statistical Sciences: 8.

[56] Bankvall CG (1974). Mechanisms of Heat Transfer in Permeable Insulation and Their Investigation in a Special Guarded Hot Plate. ASTM STP.

[57] Pelanne CM, Ho CY, Taylor RE (1960). Experiments on the Separation of Heat Transfer Mechanisms in Low-Density Fibrous Insulations. Thermal Conductivity.

[58] Pelanne CM, Hust JG (1983). The Development of Low-Density Glass-Fiber Insulation as Thermal Transmission Reference Standards. Thermal Conductivity.

[59] Lemmon EW, Huber ML, McLinden MO (2007). NIST Reference Fluid Thermodynamic and Transport Properties Database (REFPROP): 8.0. NIST Standard Reference Database.

[60] Gilbo CF (1952). Experiments with a Guarded Hot Plate Thermal Conductivity Set. Thermal Insulating Materials, ASTM STP.

[61] Zabawsky Z (1957). An Improved Guarded Hot Plate Thermal Conductivity Apparatus with Automatic Controls, Thermal Conductivity Measurements and Application of Thermal Insulations. ASTM STP.

[62] NBS (1959). Research Highlights of the National Bureau of Standards: Annual Report.

[63] Peavy ST, Bremer SG, Varner RN, Hogben D (1986). OMNITAB 80: an Interpretive System for Statistical and Numerical Data Analysis.

[64] Van Boskirk RL (1950). Resin Bonded Insulation. Modern Plastics.

[65] Zimmerman OT, Lavine I (1953). Handbook of Material Trade Names: 1953 Ed.

[66] National Bureau of Standards (1957). Standard Samples: A Catalog of Reference Materials Issued by the National Bureau of Standards. National Bureau of Standards Circular.

[67] Robinson HE, Laubitz MJ (1962). Thermal Conductivity Reference Standards, Proceedings of the 2^nd^ Conference on Thermal Conductivity (Limited Distribution).

[68] Hollingsworth M (1967). An Apparatus for Thermal Conductivity At Cryogenic Temperatures Using a Heat Flow Meter, Thermal Conductivity Measurements of Insulating Materials at Cryogenic Temperatures. ASTM STP.

[69] Hollingsworth M, McElroy DL, Tye RP (1980). Experimental Determination of the Thickness Effect in Glass Fiber Building Insulation, Thermal Insulation Performance. ASTM STP.

[70] Kinzer GR, Pelanne CM (1967). A Cryogenic Heat Flow-Meter Apparatus, Thermal Conductivity Measurements of Insulating Materials at Cryogenic Temperatures. ASTM STP.

[71] Degenne M, Klarsfeld S, Barthe M-P, Tye RP (1978). Measurement of the Thermal Resistance of Thick Low-Density Mineral Fiber Insulation, Thermal Transmission Measurements of Insulation. ASTM STP.

[72] Rose WB, McCaa DJ, Graves RS, Wysocki DC (1991). The Effect of Natural Convective Air Flows in Residential Attics on Ceiling Insulation Materials, Insulation Materials: Testing and Applications, 2nd Volume. ASTM STP.

[73] Shipp PH, Bales E, Bomberg M, Courville GE (1985). Heat Flux Sensor Applications for Below-Grade Energy Studies, Building Applications of Heat Flux Transducers. ASTM STP.

[74] Larson DC, Corneliussen RD (1983). Thermal Testing of Roof Systems, Materials and Systems for Energy Conservation in the ‘80s. ASTM STP.

[75] Abkou OA, Murali KS (1994). The effect of air cells and mortar joints on the thermal resistance concrete masonry walls. Energy and Buildings.

[76] Abkou OA, Murali K, Morsi A (1996). Thermal performance evaluation of a prefabricated fiber-reinforced plastic building envelope system. Energy and Buildings.

[77] Bomberg M, Pelanne CM, Newton WS, Ashworth T, Smith DR (1985). Analysis of Uncertainties in Calibration of a Heat Flow Meter Apparatus. Thermal Conductivity.

[78] Bomberg M, Solvason KR, Shirtliffe CJ, Tye RP (1985). Discussion of Heat Flow Meter Apparatus and Transfer Standards Used for Error Analysis, Guarded Hot Plate and Heat Flow Meter Methodology. ASTM STP.

[79] Pelanne CM, Shirtliffe CJ, Tye RP (1985). Development of a Company Wide Heat Flow Meter Calibration Program Based on the National Bureau of Standards Certified Transfer Specimens, Guarded Hot Plate and Heat Flow Meter Methodology. ASTM STP.

[80] Klems JH A Calibrated Hot Box for Testing Window Systems—Construction, Calibration, and Measurements on Prototype High-Performance Windows.

[81] (1978). Federal Register.

[82] Collins RE, Davis CA, Dey CJ, Robinson SJ, Tang J-Z, Turner GM (1993). Measurement of local heat flow in flat evacuated glazing. Int J Heat Mass Transfer.

[83] Graves RS, Yarbrough DW (1993). The Use of an Array of Heat Flux Transducers to Study Thermal Property Variations. Journal of Thermal Insulation.

[84] Hagan JR, Miller RG, McElroy DL, Kimpflen JF (1990). Long-Term R-values and Thermal Testing Requirements for Rigid Insulating Foams, Insulation Materials, Testing, and Applications. ASTM STP.

[85] Graves RS, Yarbrough DW (1992). Effect of Compression on the Material R-value of Fiberglass Batt Insulation. Journal of Thermal Insulation.

[86] Christian JE, Courville GE, Graves RS, Linkous RL, McElroy DL, Weaver FJ, Yarbrough DW, Graves RS, Wysocki DC (1991). Thermal Measurement of In-Situ and Thin-Specimen Aging of Experimental Polyisocyanurate Roof Insulation Foamed With Alternative Blowing Agents, Insulation Materials: Testing and Applications: 2nd Volume. ASTM STP.

[87] Cain B (1996). A Comparison Measurement of the Physical Properties of Round-Robin Materials.

[88] Dyck WR, Cain BJ, Moses C (1997). Determination of the Water Vapor Resistance and Thermal Resistance of Sample Materials Using a Sweating Hot Plate.

[89] Kwon YC, Yarbrough DW (2004). A Comparison of Korean Cellulose Insulation with Cellulose Insulation Manufactured in the United States of America. Journal of Building Physics.

[90] Graves RS, Yarbrough DW, McElroy DL, Ashworth T, Smith DR (1985). Apparent thermal Conductivity Measurements by an Unguarded Technique. Thermal Conductivity.

[91] Yarbrough DW, McElroy DL, Graves RS (1985). Thermal Resistance of Roof Panels and In-situ Calibration of Heat Flux Transducers. Thermal Performance of the Exterior Envelopes of Buildings III.

[92] Yarbrough DW, McElroy DL (1986). Analysis of Transient Measurements Obtained for Thermal Insulations with the ORNL Flat Tester. Journal of Thermal Insulation.

[93] Yajnik S, Roux JA (1987). Determination of Radiative Properties of Fiberglass and Foam Insulations.

[94] Roux JA (2003). Radiative Properties of High and Low Density Fiberglass Insulation in the 4-38.5 km Wavelength Region. Journal of Building Physics.

[95] Tong TW, McElroy DL, Yarbrough DW (1985). Transient Conduction and Radiation Heat Transfer in Porous Thermal Insulations. Journal of Thermal Insulation.

[96] Tong TW, McElroy DL, Yarbrough DW (1986). Analysis of Transient Heat Transfer Measurements on Porous Thermal Insulations. Journal of Thermal Insulation.

[97] Yajnik S, Roux JA, Hasselman DPH, Thomas JR (1989). Apparent Thermal Conductivity of High Density and Low Density Fiberglass Insulations. Thermal Conductivity.

[98] Lawson JR, Walton WD, Bryner NP, Amon FK (2005). Estimates of Thermal Properties for Fire Fighters’ Protective Clothing Materials. NISTIR.

[99] Vettori R (2005). Estimates of Thermal Conductivity for Unconditioned and Conditioned Materials Used in Fire Fighters’ Protective Clothing. NISTIR.

[100] Tseng C-J, Kuo K-T (2002). Thermal properties of phenolic foam insulation. Journal of the Chinese Institute of Engineers.

[101] Abdou AA, Budaiwi IM (2005). Comparison of Thermal Conductivity Measurements of Building Insulation Materials Under Various Operating Temperatures. Journal of Building Physics.

[102] Madhusudana CV (2006). Low Thermal Conductivity Measurements with a GHP Apparatus. International Heat Transfer Conference.

[103] Su LF, Miao L, Taneumra S, Xu G (2012). Low-cost and fast synthesis of nanoporous silica cryogels for thermal insulation applications. Science and Technology of Advanced Materials.

[104] Vidi S, Rausch S, Ebert HP, Löhberg A, Petry D (2013). Effective Thermal Conductivity Measurements on Supporting Structures of the Mercury Probe Bepi Colombo. International Journal of Thermophysics.

[105] Almanza O, Rodríguez-Pérez MA, De Saja JA (2004). Applicability of the Transient Plane Source Method to Measure the Thermal Conductivity of Low-Density Polyethylene Foams. Journal of Polymer Science: Part B: Polymer Physics.

[106] Zarr RR, Kumaran MK, Lagergren ES (1997). NIST/NRC-Canada Interlaboratory Comparison of Guarded Hot Plate Measurements: 1993–1997. NISTIR.

[107] Salmon D (2001). Thermal conductivity of insulations using guarded hot plates, including recent developments and sources of reference materials. Measurement Science and Technology.

[108] Kirby RK, Maglić KD, Cezairliyan A, Peletsky VE (1992). Reference Materials for Thermophysical Properties, in Compendium of Thermophysical Property Measurement Methods. Recommended Techniques and Practices.

[109] Powell FJ, Bales EL, Govan FA, Greason DM, McAllister JD (1983). Design of Round-Robin Tests with Guarded/Calibrated Hot Boxes, Guarded Hot Plates, and Heat Flow Meters. Thermal Insulation, Materials, and Systems for Energy Conservation in the ‘80s, ASTM STP.

[110] Smith DR (1997). Thermal Conductivity of Fibrous Glass Board by Guarded Hot Plates and Heat Flow Meters: An International Round-Robin. International Journal of Thermophysics.

